# Supplementary material for: UK poSt Arthroplasty Follow-up rEcommendations (UK SAFE): what does analysis of linked, routinely collected national datasets tell us about mid–late term revision risk after knee replacement?
Source: BMJ Open. 2022 Mar 9;12(3):e046900. doi: 10.1136/bmjopen-2020-046900 (PMC8915365; doi:10.1136/bmjopen-2020-046900)
Supplement: Supplementary data [file bmjopen-2020-046900supp001.pdf]

Open access Protocol

BMJ Open

Towards UK poSt Arthroplasty Follow-up rEcommendations (UK SAFE): protocol for an evaluation of the requirements for arthroplasty follow-up, and the production of consensus-based recommendations

Carolyn J Czoski Murray,[1] Sarah R Kingsbury,[2,3] Nigel K Arden,[4] Jenny Hewison,[1] Andrew Judge,[5,6] Jamie Matu,[1,2] Jamie O'Shea,[7] Rafael Pinedo-Villanueva,[4] Lindsay K Smith,[8] Chris Smith,[1] Christine M Thomas,[2,3] Robert M West,[1] Judy M Wright,[1] Philip G Conaghan,[2,3] Martin H Stone[3,9]

To cite: Czoski Murray CJ, Kingsbury SR, Arden NK, *et al*. Towards UK poSt Arthroplasty Follow-up rEcommendations (UK SAFE): protocol for an evaluation of the requirements for arthroplasty follow-up, and the production of consensus-based recommendations. *BMJ Open* 2019;**9**:e031351. doi:10.1136/bmjopen-2019-031351

► Prepublication history and additional material for this paper are available online. To view these files, please visit the journal online (http://dx.doi.org/10.1136/bmjopen-2019-031351).

PGC and MHS contributed equally.
CJCM and SRK contributed equally.

Received 29 April 2019
Revised 21 May 2019
Accepted 22 May 2019

Check for updates

© Author(s) (or their employer(s)) 2019. Re-use permitted under CC BY. Published by BMJ.

For numbered affiliations see end of article.

Correspondence to
Professor Philip G Conaghan; p.conaghan@leeds.ac.uk

ABSTRACT

Introduction Hip and knee arthroplasties have revolutionised the management of degenerative joint diseases and, due to an ageing population, are becoming increasingly common. Follow-up of joint prostheses is to identify problems in symptomatic or asymptomatic patients due to infection, osteolysis, bone loss or potential periprosthetic fracture, enabling timely intervention to prevent catastrophic failure at a later date. Early revision is usually more straight-forward surgically and less traumatic for the patient. However, routine long-term follow-up is costly and requires considerable clinical time. Therefore, some centres in the UK have curtailed this aspect of primary hip and knee arthroplasty services, doing so without an evidence base that such disinvestment is clinically or cost-effective.

Methods Given the timeline from joint replacement to revision, conducting a randomised controlled trial (RCT) to determine potential consequences of disinvestment in hip and knee arthroplasty follow-up is not feasible. Furthermore, the low revision rates of modern prostheses, less than 10% at 10 years, would necessitate thousands of patients to adequately power such a study. The huge variation in follow-up practice across the UK also limits the generalisability of an RCT. This study will therefore use a mixed-methods approach to examine the requirements for arthroplasty follow-up and produce evidence-based and consensus-based recommendations as to how, when and on whom follow-up should be conducted. Four interconnected work packages will be completed: (1) a systematic literature review; (2a) analysis of routinely collected National Health Service data from five national data sets to understand when and which patients present for revision surgery; (2b) prospective data regarding how patients currently present for revision surgery; (3) economic modelling to simulate long-term costs and quality-adjusted life years associated with different follow-up care models and (4) a Delphi-consensus process, involving all stakeholders, to develop a policy document which includes a stratification algorithm to determine appropriate follow-up care for an individual patient.

Ethics and Dissemination Favourable ethical opinion has been obtained for WP2a (RO-HES) (220520) and WP2B (220316) from the National Research Ethics Committee. Following advice from the Confidentiality Advisory Group (17/CAG/0122), data controllers for the data sets used in WP2a (RO-HES) – NHS Digital and The Phoenix Partnership – confirmed that Section 251 support was not required as no identifiable data was flowing into or out of these parties. Application for approval of WP2a (RO-HES) from the Independent Group Advising on the Release of Data (IGARD) at NHS Digital is in progress (DARS-NIC-147997). Section 251 support (17/CAG/0030) and NHS Digital approval (DARS-NIC-172121-G0Z1H-v0.11) have been

Strengths and limitations of this study

- ► Our mixed-methods approach allows us to address a question that would not be feasible to answer with a randomised controlled trial.
- ► Our study will capture data from a mixture of teaching hospitals, district general hospitals and hospitals with a special interest in joint replacement and with a geographical spread, increasing the generalisability of our results.
- ► Our economic model will be populated with routinely collected National Health Service (NHS) data of patients attending primary and hospital care in the UK, ensuring that our analysis is based on actual patient use of services, outcomes such as health-related quality of life and costs to the NHS.
- ► While our analysis is based on data sources that reflect clinical practice in England only, we believe key cost-effectiveness findings are likely to be informative for decision-making in the whole of the UK.

BMJ Open: first published as 10.1136/bmjopen-2019-031351 on 25 June 2019. Downloaded from http://bmjopen.bmj.com/ on 15 July 2019 by guest. Protected by copyright.

obtained for WP2a (NJR-HES-PROMS). ISAC (11_050MnA2R2) approval has been obtained for WP2a (CPRD-HES).

INTRODUCTION

Arguably, total hip arthroplasty (THA) and total knee arthroplasty (TKA) are the most successful surgical interventions performed in modern times. Due to an ageing population, and an obesity epidemic, hip and knee replacement procedures increase annually, rising from less than 20 000/year in the UK in 1978 to around 200 000/year in 2017.[1] The current follow-up requirements are estimated at 500 000–1 000 000 annual outpatient attendances. With limitless resources, every patient undergoing a joint arthroplasty would incur routine lifetime follow-up. The rationale for follow-up is to ensure timely detection of complications or arthroplasty failure, such as aseptic loosening, osteolysis and potential periprosthetic fracture. The cost of revision for aseptic loosening is 35% lower than that for periprosthetic fractures and has a lower incidence of complications which impact recovery.[2] However, while routine long-term follow-up of joint prostheses may support timely revision for patients with asymptomatic complications, improving long-term health outcomes, it is also costly both clinically and financially.

Orthopaedic services are already one of the poorest performers across the National Health Service (NHS) by failing to meet waiting list targets, with an estimated 8000 orthopaedic NHS breaches each month.[3] With a rapidly ageing population and medical advances that mean less stringent criteria for surgery eligibility,[4] there is no sign that demand will recede in coming years and orthopaedic services will soon reach breaking point. To reduce the burden on orthopaedic services, evidence-based consensus guidelines are required to establish how, when and on whom follow-up should be conducted.

British Hip Society (BHS) and British Orthopaedic Association (BOA) guidelines recommend outpatient follow-up at 1 and 7 years, and every 3 years thereafter for Orthopaedic Data Evaluation Panel 10A (ODEP-10A) implants, with more frequent follow-up for novel implants.[5] However, recent work revealed considerable diversity across the UK in arthroplasty follow-up pathways, in timing, how follow-up is conducted and which health professionals are involved.[6]While some centres followed-up patients beyond 10 years, others did not have an established follow-up policy and in some centres follow-up services have been curtailed or stopped entirely after an early postoperative check.[6] Notably, we do not know whether long-term follow-up is cost-effective or whether disinvestment is safe for patients.

This project aims to determine the consequences of disinvestment in hip and knee arthroplasty follow-up. Given the timeline from joint replacement to revision, with a 7% revision rate for THA and 4% revision rate for TKA at 14 years, conducting a randomised controlled trial to address this question is not feasible. Moreover, the huge variation in follow-up practice across the UK limits the generalisability of the results of an RCT. We will therefore use a mixed-methods approach to comprehensively evaluate the requirements for arthroplasty follow-up and will use this evidence to inform the development of consensus-based recommendations and a policy document which includes a stratification algorithm to determine appropriate follow-up for individual patients. Disinvestment is a complex and often contentious issue. We plan to make use of published recommendations[7] to ensure that the results of this work are understood and considered as a genuine attempt to use the best evidence available to ensure that the NHS gets value for money and that patients remain safe.

METHODS AND ANALYSIS

Study objectives

A. Identify who needs follow-up and when this should occur for primary THA, TKA and unicompartmental knee arthroplasty (UKA) surgery by making use of routinely collected NHS data.
B. Understand the patient journey (in primary and secondary care) to revision surgery by recruiting patients admitted for elective and emergency hip and knee revision surgery.
C. Establish how and when patients are identified for revision, why some patients are missed from regular follow-up and present acutely with fracture around the implant (periprosthetic fracture), by using prospective and retrospective data.
D. Identify the most appropriate and cost-effective follow-up pathway to minimise potential harm to patients by undertaking cost-effectiveness modelling.
E. Provide evidence-based and consensus-based recommendations on how follow-up of primary THA and TKA should be conducted.

Design

This is a mixed-methods study using a variety of data sources consisting of four interconnected work packages (WP): (1) a systematic literature review; (2a) analysis of routinely-collected NHS data to understand when and which patient present for revision surgery; (2b) prospective data regarding how patients currently present for revision surgery collected on around 455 patients prior to elective or emergency revision surgery; (3) economic modelling to simulate long-term costs and quality adjusted life years associated with different follow-up models; (4) a Delphi-consensus process, incorporating all previous WPs and involving all stakeholders, to develop a policy document which includes a stratification algorithm to determine appropriate follow-up for an individual patient.

WP1: systematic review

The aim of the review is to evaluate different models of routine long-term follow-up care after TKA/THA/UKA. This systematic review will establish a robust evidence base

BMJ Open: first published as 10.1136/bmjopen-2019-031351 on 25 June 2019. Downloaded from http://bmjopen.bmj.com/ on 15 July 2019 by guest. Protected by copyright.

Supplemental material BMJ Publishing Group Limited (BMJ) disclaims all liability and responsibility arising from any reliance placed on this supplemental material which has been supplied by the author(s) BMJ Open

for the cost-effectiveness modelling (WP3) and consensus guideline development (WP4).

Registration

This systematic review will be undertaken following Cochrane Collaboration methods[8] and reported in accordance with Preferred Reporting Items for Systematic Review and Meta-analyses guidelines.[9] It has been prospectively registered with PROSPERO (CRD42017053017).

Searches

A comprehensive literature search will be undertaken with the aim of retrieving all relevant literature, published or unpublished, which evaluated the effectiveness of long-term follow-up after primary TKA/THA/UKA. A range of information sources will be searched: BIOSIS, CINAHL, ClinicalTrials.gov, The Cochrane Library, Embase, Health Management Information Consortium, IDEAS (RePEC), Ovid Medline(R), ProQuest Dissertations and Theses, PsycINFO, PubMed and Web of Science. Reference lists of included studies will be reviewed for potentially relevant articles. A sample search strategy is detailed in the online supplementary appendix A. No date or language restrictions will be applied.

Criteria for selection of studies

All study designs will be included which (1) consider the clinical and/or cost effectiveness of routine long-term (>5 years) follow-up care after primary THA, TKA or UKA; (2) describe patient safety issues associated with routine follow-up or (3) consider the acceptability of new care pathways from the perspective of the patient and/or practitioner. Studies will be excluded if they do not report specific patient-related outcome measures or appropriate health utility measures.

Selection of studies

Titles/abstracts of identified studies will be screened for eligibility by one experienced reviewer with a random selection (25%) independently screened by a second. Potential studies will be retrieved in full text and reviewed against the inclusion/exclusion criteria independently by the same two reviewers, with a third reviewer used to settle any disputes.

Data extraction

Data will be extracted by a single reviewer using a standardised proforma capturing (1) purpose and design; (2) methodological characteristics; (3) information relating to quality assessment and (4) outcome data relating to the clinical and cost-effectiveness of routine long-term follow-up care.

Quality assessment

The Cochrane Risk of Bias assessment tool will be used for experimental studies,[10] and the Newcastle-Ottawa scales for cohort and case–control studies.[11] Qualitative literature will be assessed using critical interpretive synthesis.[12] Economic evaluations will be assessed using the Drummond checklist.[13] Studies will be evaluated independently by two reviewers, with a third to settle any disputes. Studies at high risk of bias will not be excluded and conclusions will incorporate observed biases.

Evidence synthesis

The design, methodological characteristics, study quality and main findings will be summarised in narrative and tabular form. We anticipate substantial heterogeneity among included studies precluding the use of meta-analysis techniques.

WP2a: Analysis of routinely collected NHS data

This WP will use routinely collected NHS data to determine when revision happens and to identify patients most likely to require revision in order to target when and on whom follow-up should occur.

Data sources

Data from five national datasets will be used: (1) Clinical Practice Research Database (CPRD),[14] (2) ResearchOne (RO),[15] (3) Hospital Episode Statistics (HES),[16] (4) National Joint Registry (NJR)[17] and (5) patient reported outcome measures (PROMs).[18]

Three linked data sets will be constructed for analysis: (a) CPRD–HES–PROMS, which preexists at the University of Oxford, (b) RO–HES will be constructed and analysed at the University of Leeds. Linkage will be undertaken by NHS Digital on the basis of pseudonyms generated from NHS numbers by the data providers. (c) NJR–HES–PROMS will be constructed and analysed at the University of Oxford. Linkages will be undertaken by NHS Digital, using an agreed set of common patient identifiers, including NHS number. Data sets (a) and (b) provide a primary care view (eg, prior diagnoses, prescribing) and include different, representative patient populations for cross-validation; data set (c) provides a secondary care view (eg, surgeon, procedure details).

Data analysis

The primary outcome of the analysis will be mid-late term revision (>5 years post-primary surgery), defined as the removal, exchange or addition of any of the components of arthroplasty. Exposures will include secondary care predictors, including patient level characteristics recorded in NJR and HES (eg, age, body mass index (BMI)), surgical and operative factors and symptoms of pain, function and health-related quality of life preoperatively and 6 months post-surgery from PROMS, and primary care predictors, including patient demographics, comorbidities and use of drugs which can affect fracture risk. Survival analysis will be used to model time to revision.[19 20] The smoothed Nelson-Aalen cumulative hazard rate will be examined to identify any peak in the mid-long term risk of revision. Cox proportional hazards regression modelling will be used to identify preoperative, perioperative and postoperative predictors of mid-late term revision, for example, age, BMI, comorbidities, implant type, surgeon skill and postoperative problems. Competing risk

BMJ Open: first published as 10.1136/bmjopen-2019-031351 on 25 June 2019. Downloaded from http://bmjopen.bmj.com/ on 15 July 2019 by guest. Protected by copyright.

regression will be used, since mortality can be regarded as a competing risk for revision surgery.[21 22] To account for clustering within the data (such as patients nested within hospitals), a multilevel survival model will be fitted by extending the survival regression model to include a frailty term with a Gaussian distribution.[23]

WP2b part 1: multicentre observational prospective cohort

Prospective data collection from patients undergoing revision surgery.

Objectives:

- Identify all recent (previous 12 months) medical appointments and advice sessions related to the index joint in primary and secondary care.
- Establish if the patient has been seen by orthopaedic health professionals from 12 months after primary surgery until this hospital admission, that is, was the revision directed by routine follow-up.

Design

A multicentre, observational, single visit, prospective cohort study of patients admitted for revision hip or knee surgery.

Population

Patients presenting for elective and emergency revision surgery of a primary THA, TKA or UKA and who are able and willing to provide written informed consent will be included in the study. Patients will be excluded if they have had previous revision surgery; metal-on-metal primary joint replacement or hip hemiarthroplasty. Participants will be recruited from a sample of hospitals selected to provide geographical spread and representation of teaching hospitals, district general hospitals and hospitals with a special interest in joint replacement

Data collection

A participant case report form (CRF) will capture details of follow-up after primary surgery and pathway to current revision surgery, including symptom state. An investigator CRF will extract data from medical notes including demographics (age, gender, diagnosis leading to primary surgery, medical history), general practitioner and hospital appointments, details of primary and revision surgery (including implant type, complications, length of stay). The participant CRF will be piloted with the Leeds Biomedical Research Centre Patient and Public Involvement (PPI) group and the investigator CRF with two research nurses to ascertain the comprehension, usability and completeness of data subsequently extracted.

Sample size

We will use stratified sampling to recruit centres of varying size and anticipate that the average number of patients per centre will be 45 (based on NJR records and information from prospective centres). We initially anticipated the recruitment of 25 centres. With a recruitment rate of 60%, this gave 27 recruited patients from 25 centres (n=675). We do not know the intraclass correlation coefficient (ICC) for our primary outcome ('Was the revision a result of routine follow-up?'), but we anticipate it to be in the region of 0.01–0.05. To be conservative, we use ICC=0.05. This gives a design factor of 2.3 and hence an effective sample size of 293 after accounting for clustering within centre. The enrolment of 35 centres reduced the design factor to 1.6 and the total sample size required to 455. From previous research,[6] we estimate that the rate of our primary outcome is 20% so that the effective number of events will be 58. Hence, we will have sufficient power for our logistic regression to robustly estimate the coefficients of up to five potential risk factors derived from our brief patient survey.[24]

Analysis

The primary outcome will be 'revision identified through routine follow-up', and this will be modelled through a multilevel logistic regression model, with a centre-level random intercept of particular interest. The size of the centre-level effect will be assessed as the proportion of variance explained and will also be assessed through a likelihood ratio test. Up to five factors from the patient questionnaire will be explored as fixed effects at the patient level. This will adjust for case mix. Factors that are found to be both clinically and statistically significant could potentially contribute to a stratified approach to follow-up.

WP2b part 2: qualitative study

Building on previous work highlighting the changes in follow-up practice,[6] this WP aims to explore the rationale and motivating factors behind these changes, the facilitators and the evidence considered when implementing new pathways, including no follow-up.

Sampling

A sample of n=20–30 orthopaedic practitioners and/or unit managers will be recruited. Purposive sampling via sampling matrix will recruit participants with different experiences of a range of follow-up pathways while reflecting NHS trust type, geographical area (urban, rural); socioeconomic area (low/high socioeconomic status) and diverse ethnicity. Some selection criteria are likely to be nested (eg, hospital type, geographical area) and care will be taken to ensure that all viewpoints are represented.

Data collection

Semistructured, telephone interviews following a topic guide refined from the literature review and expert opinion (clinician coapplicants/advisors and PPI members). The researcher will probe pertinent initial responses and expand on issues raised. Interviews will be recorded and transcribed verbatim.

Data analysis

The guiding approach will be framework analysis.[25] Data analysis will comprise five stages: (1) data familiarisation;

BMJ Open: first published as 10.1136/bmjopen-2019-031351 on 25 June 2019. Downloaded from http://bmjopen.bmj.com/ on 15 July 2019 by guest. Protected by copyright.

(2) identifying the thematic framework; (3) indexing; (4) charting and (5) mapping and interpreting. The process of familiarisation enables the researcher to identify emerging themes or issues in the data. Little is known about why NHS trusts have chosen to either withdraw follow-up care or change the way it is delivered. The evidence generated from the literature review and input from our clinical coapplicants will be used to help identify and refine the thematic framework. Themes are flexible and can be modified in the light of new data, and a process of constant comparison will be undertaken across themes and cases.

WP3

As previous work conducted by members of our team has identified considerable heterogeneity in current follow-up pathways,[6] our cost-effectiveness analysis will compare the relative costs and quality-adjusted life years associated with having follow-up compared with not having follow-up. A third hypothetical scenario of a virtual follow-up will be considered.

Comparators

Both the findings from our systematic review and the prospective cohort will inform the criteria to be used to identify patients as having or not having follow-up. The 7-year reference point for a follow-up currently suggested by BHS and BOA guidelines is likely to be incorporated. Patients having an orthopaedic outpatient appointment around the reference point(s) following a primary arthroplasty will be used to group patients in the CPRD–HES–PROMS data set into the follow-up and no follow-up groups. Joint-specific revision procedures will be identified by OPCS-4 codes as reported in the Admitted Patient Care data set within HES, with corresponding linked records to primary care and PROMS.

Model structure

To identify the most appropriate modelling approach for the question and data at hand, we will conduct a series of preliminary analysis to determine if a cohort-level or patient-level decision analytic model should be employed. Previous models examining the long-term cost-effectiveness of hip and knee replacements have used cohort Markov models.[26 27] Analyses will include associations between patients' characteristics and revision rates, health utilities and costs and whether the risk for revision depends on the time patients stay unrevised after their primary. Regardless of the chosen model type, the key health state or event will be revision arthroplasty, with death and complications also considered. The model will be designed to cover patients' lifetime and analysed from an NHS and Personal Social Services perspective, with discounting of costs and outcomes as per current guide to the methods of technology appraisal.[28]

Model inputs

WP2 data sets will be used to quantify primary and hospital healthcare resource use for comparator groups of follow-up care models through estimation of NHS costs and health-related quality of life (HRQoL). The economic model will simulate long-term costs and quality adjusted life years (QALYs) associated with each care model. Primary care costs will include consultations, and hospital costs will be derived by grouping hospital episodes into Health Resource Groups, a set of casemix groupings utilising similar levels of healthcare resources. Panel data regression analysis[29–31] will be used to estimate hospital costs conditional on patient characteristics and comorbidities. QALYs and transition probabilities will be derived from the linked data sets and published literature as needed. The hypothetical costs of virtual follow-up will be based on similar virtual clinic alternatives previously studied and NHS X-ray-associated costs.

Analysis

Cost-effectiveness analyses will be performed separately for relevant patient subgroups based on gender, age and other potential covariates for which data may be available. As with all economic models, a number of assumptions will be made, and their plausibility and potential impact discussed, relating to model structure and input parameters for transition probabilities, health utilities and costs, including the cost of periprosthetic fractures if no reference is found for these in the literature. Sensitivity analyses will be conducted to explore the uncertainty associated with key assumptions and model parameters and the implications of using different estimates discussed.

WP4: Delphi-consensus process

This WP will use the collective evidence from WP1–3 to inform a consensus process to determine appropriate follow-up care pathways for hip and knee arthroplasty.

Evidence gathered from WP1–3 will feed into a consensus panel workshop. We intend to use methods employed by the National Institute for Health and Care Excellence (NICE) in both the technology assessment committees and Guideline Development Groups. The expert stakeholders invited to attend will have a special interest in patient follow-up after hip or knee replacement surgery. Participants will include patients, orthopaedic surgeons, arthroplasty practitioners, NHS managers and commissioners, manufacturers and representatives of the major orthopaedic bodies (including BOA, BHS and BASK). The purpose of this exercise is to consider the evidence and obtain agreement for future care pathways, supported by the evidence of their effectiveness and cost-effectiveness, to be recommended and adopted across the NHS. Following the NICE consensus model all participants will receive summaries of the main research findings in advance. There will be presentations from the work-stream leaders to outline the evidence for consideration.

Robert *et al*[7] demonstrate that decommissioning is often about more than the 'evidence' and that withdrawal of previously available services is often seen as being driven by the wrong kind of evidence, based on cost data and

BMJ Open: first published as 10.1136/bmjopen-2019-031351 on 25 June 2019. Downloaded from http://bmjopen.bmj.com/ on 15 July 2019 by guest. Protected by copyright.

Supplemental material
BMJ Publishing Group Limited (BMJ) disclaims all liability and responsibility arising from any reliance placed on this supplemental material which has been supplied by the author(s)
BMJ Open

political priorities and not on what patients and service users value.[7] It is a complex issue, perhaps as contentious as NICE decisions when they do not fund an effective intervention because it exceeds the threshold. However, NICE investment decisions are made with the explicit understanding that, with no increase in the budget, there must be some displacement of other healthcare technologies.[32] We plan to make use of the recommendations for engagement and the use of evidence outlined in Robert *et al* to ensure the results of this work are understood and considered as a genuine attempt to use the best evidence available to ensure that the NHS gets value for money and that patients remain safe.

Patient and public involvement

Members of the NIHR Leeds BRC, Oxford and Bristol PPI groups are involved in UK SAFE. The PPI co-applicant is a member of the study steering committee and contributes across all WPs. Two independent PPI advisors sit on the Independent Advisory Group. Specific areas where lay involvement will be pivotal include the interpretation of results of the systematic review, the expert panel discussion and consensus process, study oversight (steering group), preparation of patient material and study results and contribution to reports and newsletters for patients and NHS staff.

ETHICS AND DISSEMINATION

All studies will be conducted in accordance with the principles of Good Clinical Practice, and the UK Policy Framework for Health and Social Care Research, 2018. Favourable ethical opinion has been obtained for WP2a (RO-HES) (220520) and WP2B (220316) from the National Research Ethics Committee. Following advice from the Confidentiality Advisory Group (17/CAG/0122), data controllers for the data sets used in WP2a (RO–HES)—NHS Digital and The Phoenix Partnership—confirmed that Section 251 support was not required as no identifiable data was flowing into or out of these parties. Application for approval of WP2a (RO–HES) from the Independent Group Advising on the Release of Data (IGARD) at NHS Digital is in progress (DARS-NIC-147997). Section 251 support (17/CAG/0030) and NHS Digital approval (DARS-NIC-172121-G0Z1H-v0.11) have been obtained for WP2a (NJR-HES-PROMS). ISAC (11_050MnA2R2) approval has been obtained for WP2a (CPRD–HES).

At the end of the project, outputs will be disseminated nationally in the form of an executive summary statement of the agreed pathway/s through appropriate NHS Networks, NICE, the NHS England Elective Orthopaedics Sub-committee, the NHS Institute for Innovation and Improvement and professional societies, including BHS, BOA, BASK, Arthroplasty Care Practitioners Association and the NJR. Dissemination will be key to developing a culture of 'finding the best way of doing something and doing it everywhere' to significantly reduce wastage of clinical resources and optimise NHS spend. We will put forward the consensus statement to each society's AGM for adoption as a resolution. Internationally, dissemination platforms are in place through the International Society of Arthroplasty Registers (ISAR) and the European Federation of National Associations of Orthopaedics and Traumatology. A lay summary of the project will be produced for study participants. Findings will also be presented at relevant orthopaedic and methodological conferences, such as the BOA and the Exploiting Existing Data for Health Research conference. The chief investigator and co-applicants will be named as authors on main publications, and an appropriate first author agreed through discussion. Other key individuals will be included as authors or contributors as appropriate, at the discretion of the Senior Management Group. Any disputes relating to authorship will be resolved by the Steering Committee.

The Chair and Independent members of the Steering Committee will be acknowledged, but will not qualify for full authorship, in order to maintain their independence. Individual collaborators must not publish data concerning their participants' which are directly relevant to the questions posed in the study until the main results of the study have been published.

CONCLUSION

This research will deliver the first research-supported, best-for-patient, joint-specific, cost-effective recommendations for follow-up pathways, providing a gold standard for clinical excellence and follow-up advice for patients, surgeons, purchasers and the NHS as a whole. Value is not limited to the UK, but has substantial global impact potential.

The impact of this work will be to reduce the burden on patients and the NHS in terms of outpatient visits and clinical tests that do not add benefit, while optimising detection of potential problems. From an NHS perspective, this work will provide managers with economic and clinical information on arthroplasty follow-up to inform service planning and delivery, and the role of arthroplasty practitioners in this service, with the potential to reduce geographical disparity through NHS trusts modelling their service provision on a national evidence-based guideline; provide orthopaedic surgeons with guidance on follow-up, including patient and economic considerations of factors involved; produce arthroplasty follow-up guidelines for adoption by the relevant specialist societies and information for their members. From a patient perspective, this work will help to inform patients about follow-up practice, empower them to make choices about future healthcare relating to their joint arthroplasty and provide reassurance that their follow-up pathway is appropriate

The outputs of this project, in terms of evidence-based support for timing of follow-up and identification of the most cost-effective follow-up model, fit directly within the

BMJ Open: first published as 10.1136/bmjopen-2019-031351 on 25 June 2019. Downloaded from http://bmjopen.bmj.com/ on 15 July 2019 by guest. Protected by copyright.

Czoski Murray CJ, *et al. BMJ Open* 2019;**9**:e031351. doi:10.1136/bmjopen-2019-031351

Smith LK, *et al. BMJ Open* 2022; 12:e046900. doi: 10.1136/bmjopen-2020-046900

NHS framework for improving outcomes from elective procedures. Rationalising current diversity of follow-up practices should enable substantial savings for the NHS. We envisage outputs to be readily applicable to the wider NHS, not only hip and knee but also other joint replacements. With the committed support of key national and international organisations already in place, we anticipate that these guidelines will be positively received and that implementation will be widespread.

Author affiliations
[1]Leeds Institute of Health Sciences, University of Leeds, Leeds, UK
[2]Leeds Institute of Rheumatic and Musculoskeletal Medicine, University of Leeds, Leeds, UK
[3]NIHR Leeds Biomedical Research Centre, Leeds, UK
[4]Nuffield Department of Orthopaedics Rheumatology and Musculoskeletal Sciences, University of Oxford, Oxford, UK
[5]Musculoskeletal Research Unit, Bristol Medical School, University of Bristol, Southmead Hospital, Bristol, UK
[6]MRC Lifecourse Epidemiology Unit, Southampton, UK
[7]Manor Park Surgery, Leeds, UK
[8]Trauma and Orthopaedics, Weston Area Health NHS Trust, Weston-super-Mare, UK
[9]Department of Orthopaedic Surgery, Leeds Teaching Hospitals NHS Trust, Leeds, Leeds, UK

Contributors All authors contributed to the planning and design of this study, drafted the article, revised the article and approved the final manuscript.

Funding This article presents independent research funded by the National Institute for Health Research (NIHR) Health Services and Delivery Research Programme (14/70/146) and by the NIHR Leeds Biomedical Research Centre (BRC).

Disclaimer The views expressed are those of the author(s) and not necessarily those of the NIHR or the Department of Health and Social Care.

Competing interests None declared.

Patient consent for publication Not required.

Provenance and peer review Not commissioned; peer reviewed for ethical and funding approval prior to submission.

Open access This is an open access article distributed in accordance with the Creative Commons Attribution 4.0 Unported (CC BY 4.0) license, which permits others to copy, redistribute, remix, transform and build upon this work for any purpose, provided the original work is properly cited, a link to the licence is given, and indication of whether changes were made. See: https://creativecommons.org/licenses/by/4.0/.

REFERENCES

1. National Joint Registry for England, Wales. Northern Ireland and the Isle of Man: 15th Annual report. 2018 http://www.njrreports.org.uk/
2. Vanhegan IS, Malik AK, Jayakumar P, *et al*. A financial analysis of revision hip arthroplasty: the economic burden in relation to the national tariff. *J Bone Joint Surg Br* 2012;94:619–23.
3. Briggs T. *Getting it right first time. Improving the quality of orthopaedic care within the National Health Service in England*, 2012.
4. Singh JA. Epidemiology of knee and hip arthroplasty: a systematic review. *Open Orthop J* 2011;5:80–5.
5. British Orthopaedic Association. *Primary total hip replacement: a guide to good practice*, 2012.
6. Smith LK. A survey of the current state of hip arthroplasty surveillance in the United Kingdom. *Musculoskeletal Care* 2014;12:232–8.
7. Robert G, Harlock J, Williams I. Disentangling rhetoric and reality: an international Delphi study of factors and processes that facilitate the successful implementation of decisions to decommission healthcare services. *Implementation Science* 2014;9:123.
8. Higgins JPT. *Cochrane Handbook for Systematic Reviews of Interventions Version 5.1.0*: The Cochrane Collaboration, 2011. Available from. http://handbook.cochrane.org
9. Liberati A, Altman DG, Tetzlaff J, *et al*. The PRISMA statement for reporting systematic reviews and meta-analyses of studies that evaluate health care interventions: explanation and elaboration. *PLoS Med* 2009;6:e1000100.
10. Higgins JP, Altman DG, Sterne JA. Chapter 8: Assessing risk of bias in included studies in Cochrane handbook for systematic reviews of interventions Version 5.1.0. 2011 http://handbook.cochrane.org
11. Wells G, Shea B, O'Connell D, *et al*. The Newcastle-Ottawa Scale (NOS) for assessing the quality of nonrandomised studies in meta-analyses. 2014 http://www.ohri.ca/programs/clinical_epidemiology/oxford.asp;
12. Dixon-Woods M, Cavers D, Agarwal S, *et al*. Conducting a critical interpretive synthesis of the literature on access to healthcare by vulnerable groups. *BMC Med Res Methodol* 2006;6:35.
13. Drummond MF, Schulpher MJ, Claxton K, *et al*. *Methods for the Economic Evaluation of Health Care Programmes*. 4th edn. Oxford: Oxford University Press, 2015.
14. Clinical Practice Research Datalink. http://www.cprd.com
15. ResearchOne. http://www.researchone.org
16. Hospital Episode Statistics (HES). http://www.hscic.gov.uk/hes
17. Welcome from the National Joint Registry for England, Wales, Northern Ireland and the Isle of Man. http://www.njrcentre.org.uk
18. http://www.hscic.gov.uk/hesproms
19. Pinedo-Villanueva R, Turner D, Raftery JP, *et al*. Outcomes after total hip replacement. *Osteoarthritis Cartilage* 2014;22:S214.
20. Pinedo-Villanueva R, Turner D, Raftery JP, *et al*. Primary care costs attributable to osteoarthritis before total hip replacement. *Osteoarthritis Cartilage* 2014;22:S210–1.
21. Jason PF, Gray RJ. A proportional hazards model for the subdistribution of a competing risk. *Journal of the American Statistical Association* 1999;94:496–509.
22. Prieto-Alhambra D, Pagès-Castellà A, Wallace G, *et al*. Predictors of fracture while on treatment with oral bisphosphonates: a population-based cohort study. *J Bone Miner Res* 2014;29:268–74.
23. Hirsch K, Wienke A. Software for semiparametric shared gamma and log-normal frailty models: An overview. *Comput Methods Programs Biomed* 2012;107:582–97.
24. Peduzzi P, Concato J, Kemper E, *et al*. A simulation study of the number of events per variable in logistic regression analysis. *J Clin Epidemiol* 1996;49:1373–9.
25. Wallace G, Judge A, Prieto-Alhambra D, *et al*. The effect of body mass index on the risk of post-operative complications during the 6 months following total hip replacement or total knee replacement surgery. *Osteoarthritis Cartilage* 2014;22:918–27.
26. Burn E, Liddle AD, Hamilton TW, *et al*. Cost-effectiveness of unicompartmental compared with total knee replacement: a population-based study using data from the National Joint Registry for England and Wales. *BMJ Open* 2018;8:e020977.
27. Fitzpatrick R, Shortall E, Sculpher M, *et al*. Primary total hip replacement surgery: a systematic review of outcomes and modelling of cost-effectiveness associated with different prostheses. *Health Technol Assess* 1998;2:1–64.
28. Guide to the methods of technology appraisal 2013. https://www.nice.org.uk/process/pmg9/chapter/foreword
29. Basu A, Rathouz PJ. Estimating marginal and incremental effects on health outcomes using flexible link and variance function models. *Biostatistics* 2005;6:93–109.
30. Clarke P, Leal J, Kelman C, *et al*. Estimating the cost of complications of diabetes in Australia using administrative health-care data. *Value Health* 2008;11:199–206.
31. Mullahy J. Much ado about two: reconsidering retransformation and the two-part model in health econometrics. *J Health Econ* 1998;17:247–81.
32. NICE technology appraisal guidance. https://www.nice.org.uk/about/what-we-do/our-programmes/nice-guidance/nice-technology-appraisal-guidance

BMJ Open: first published as 10.1136/bmjopen-2019-031351 on 25 June 2019. Downloaded from http://bmjopen.bmj.com/ on 15 July 2019 by guest. Protected by copyright.
