# Supplementary material for: UK poSt Arthroplasty Follow-up rEcommendations (UK SAFE): what does analysis of linked, routinely collected national datasets tell us about mid–late term revision risk after knee replacement?
Source: BMJ Open. 2022 Mar 9;12(3):e046900. doi: 10.1136/bmjopen-2020-046900 (PMC8915365; doi:10.1136/bmjopen-2020-046900)
Supplement: Supplementary data [file bmjopen-2020-046900supp003.pdf]

# Supplementary file III: Additional results

**Table A.** Descriptive statistics for the CPRD-HES linked datasets

| | Knee Replacement |
|---|---|
| Year of primary | |
| 1995-1999 | 995 (5.7%) |
| 2000-2004 | 4486 (25.8%) |
| 2005-2009 | 8415 (48.4%) |
| 2010-2011 | 3482 (20.0%) |
| Age at primary | 69.4 (SD 9.2) |
| Sex | |
| Female | 9963 (57.3%) |
| Male | 7415 (42.7%) |
| Body mass index | |
| Underweight | 48 (0.4%) |
| Normal | 2204 (16.2%) |
| Overweight | 5239 (38.6%) |
| Obese Class I (Moderately obese) | 3777 (27.8%) |
| Obese Class II and higher | 2306 (17.0%) |
| Index of Multiple Deprivation (IMD) quintiles | |
| Least deprived | 3890 (22.4%) |
| 2 | 4145 (23.9%) |
| 3 | 3918 (22.6%) |
| 4 | 3078 (17.7%) |
| Most deprived | 2328 (13.4%) |
| Region | |
| East Midlands | 706 (4.1%) |
| East of England | 2003 (11.5%) |
| London | 1464 (8.4%) |
| North East | 434 (2.5%) |
| North West | 2551 (14.7%) |
| South Central | 2605 (15.0%) |
| South East Coast | 2144 (12.3%) |
| South West | 2386 (13.7%) |
| West Midlands | 2320 (13.4%) |
| Yorkshire & The Humber | 765 (4.4%) |
| Smoker | |
| Ex-smoker | 5122 (34.6%) |
| Non-smoker | 8310 (56.2%) |
| Current | 1368 (9.2%) |
| Alcohol | |

| | |
|---|---|
| Ex-smoker | 322 (2.7%) |
| No | 2242 (18.8%) |
| Yes | 9371 (78.5%) |
| Recorded diagnosis of hip OA | 6841 (39.4%) |
| Hip Fracture prior primary surgery | 119 (0.7%) |
| Fracture in pelvis, proximal/humerus, wrist/forearm, spine or rib | 537 (3.1%) |
| Comorbidities | |
| asthma | 1713 (9.9%) |
| malabsorption | 42 (0.2%) |
| inflammatory bowel disease | 128 (0.7%) |
| hypertension | 6106 (35.1%) |
| hyperlipidaemia | 2223 (12.8%) |
| ischaemic heart disease | 1685 (9.7%) |
| myocardial infarction | 345 (2.0%) |
| stroke/cerebrovascular disease | 585 (3.4%) |
| chronic pulmonary disease | 498 (2.9%) |
| chronic kidney failure | 1277 (7.4%) |
| cancer | 1446 (8.3%) |
| diabetes | 1774 (10.2%) |
| Drugs which can affect fracture risk prior primary surge | |
| Calcium and vitamin D supplements | 1377 (7.9%) |
| Bisphosphonates | 1161 (6.7%) |
| Selective oestrogen receptor modulators | 33 (0.2%) |
| Oral glucocorticosteroid therapy | 3521 (20.3%) |
| Drugs prior primary surgery | |
| Proton pump inhibitors | 7586 (43.7%) |
| Anti-arrhythmics | 1700 (9.8%) |
| Anticonvulsants | 865 (5.0%) |
| Antidepressants | 5875 (33.8%) |
| Anti-Parkinson drugs | 305 (1.8%) |
| Statins | 5697 (32.8%) |
| Thiazide diuretics | 8498 (48.9%) |
| Anxiolitics | 3406 (19.6%) |
| Painkillers/anti-inflammatory drugs | |
| NSAIDs | 15406 (88.7%) |
| NSAID cox | 3155 (18.2%) |
| Paracetamol | 14438 (83.1%) |
| Partial Opiates | 13334 (76.7%) |
| Total Opiates | 6459 (37.2%) |
| Steroid iFuse Implant System® Minimally Invasive Arthrodesis (iMIA) | 5401 (31.1%) |
| DDDs (daily defined dose) 1-year prior surgery | |

| | |
|---|---|
| Calcium and vitamin D supplements | |
| No dose | 16001 (92.1%) |
| <120 DDD | 281 (1.6%) |
| >=120 to 340 DDD | 503 (2.9%) |
| >340 DDD | 222 (1.3%) |
| Dose missing | 371 (2.1%) |
| Bisphosphonates | |
| No dose | 16217 (93.3%) |
| <140 DDD | 229 (1.3%) |
| >=140 to 340 DDD | 374 (2.2%) |
| >340 DDD | 260 (1.5%) |
| Dose missing | 298 (1.7%) |
| Selective oestrogen receptor modulators | |
| No dose | 17345 (99.8%) |
| <280 DDD | 8 (0.1%) |
| >=280 to 390 DDD | 8 (0.1%) |
| >390 DDD | 0 (0%) |
| Dose missing | 17 (0.1%) |
| Oral glucocorticosteroid therapy | |
| No dose | 13857 (79.7%) |
| <30 DDD | 493 (2.8%) |
| >=30 to 280 DDD | 458 (2.6%) |
| >280 DDD | 316 (1.8%) |
| Dose missing | 2254 (13.0%) |
| Proton pump inhibitors (no dose) | |
| No dose | 9792 (56.4%) |
| <85 DDD | 1376 (7.9%) |
| >=85 to 365 DDD | 2847 (16.4%) |
| >365 DDD | 995 (5.7%) |
| Dose missing | 2368 (13.6%) |
| Anti-arrhythmics (no dose) | |
| No dose | 15678 (90.2%) |
| <170 DDD | 159 (0.9%) |
| >=170 to 365 DDD | 241 (1.4%) |
| >365 DDD | 158 (0.9%) |
| Dose missing | 1142 (6.6%) |
| Anticonvulsants | |
| No dose | 16513 (95.0%) |
| <85 DDD | 132 (0.8%) |
| >=85 to 365 DDD | 212 (1.2%) |
| >365 DDD | 111 (0.6%) |
| Dose missing | 410 (2.4%) |
| Antidepressants | |

| | |
|---|---|
| No dose | 11503 (66.2%) |
| <85 DDD | 786 (4.5%) |
| >=85 to 365 DDD | 1418 (8.2%) |
| >365 DDD | 565 (3.3%) |
| Dose missing | 3106 (17.9%) |
| Anti-Parkinson drugs | |
| No dose | 17073 (98.2%) |
| <200 DDD | 36 (0.2%) |
| >=200 to 600 DDD | 90 (0.5%) |
| >600 DDD | 41 (0.2%) |
| Dose missing | 138 (0.8%) |
| Statins | |
| No dose | 11681 (67.2%) |
| <280 DDD | 1248 (7.2%) |
| >=280 to 370 DDD | 2522 (14.5%) |
| >370 DDD | 1383 (8.0%) |
| Dose missing | 544 (3.1%) |
| Thiazide diuretics | |
| No dose | 8880 (51.1%) |
| <225 DDD | 1678 (9.7%) |
| >=225 to 390 DDD | 2826 (16.3%) |
| >390 DDD | 1565 (9.0%) |
| Dose missing | 2429 (14.0%) |
| Anxiolitics | |
| No dose | 13972 (80.4%) |
| <30 DDD | 367 (2.1%) |
| >=30 to 350 DDD | 531 (3.1%) |
| >350 DDD | 344 (2.0%) |
| Dose missing | 2164 (12.5%) |
| NSAIDs | |
| No dose | 1972 (11.4%) |
| <60 DDD | 2428 (14.0%) |
| >=60 to 300 DDD | 4602 (26.5%) |
| >300 DDD | 2352 (13.5%) |
| Dose missing | 6024 (34.7%) |
| NSAID cox | |
| No dose | 14223 (81.8%) |
| <60 DDD | 355 (2.0%) |
| >=60 to 280 DDD | 553 (3.2%) |
| >280 DDD | 267 (1.5%) |
| Dose missing | 1980 (11.4%) |
| Paracetamol | |
| No dose | 2940 (16.9%) |

| | |
|---|---|
| <40 DDD | 2796 (16.1%) |
| >=40 to 200 DDD | 5521 (31.8%) |
| >200 DDD | 2425 (14.0%) |
| Dose missing | 3696 (21.3%) |
| Opioids mix | |
| No dose | 4044 (23.3%) |
| <30 DDD | 2074 (11.9%) |
| >=30 to 180 DDD | 4002 (23.0%) |
| >180 DDD | 1976 (11.4%) |
| Dose missing | 5282 (30.4%) |
| Opioids total | |
| No dose | 10919 (62.8%) |
| <200 DDD | 995 (5.7%) |
| >=200 to 600 DDD | 1916 (11.0%) |
| >600 DDD | 871 (5.0%) |
| Dose missing | 2677 (15.4%) |
| Steroid IMIA | |
| No dose | 11977 (68.9%) |
| <55 DDD | 1292 (7.4%) |
| >=55 DDD | 597 (3.4%) |
| Dose missing | 3512 (20.2%) |

**Table B.** Descriptive statistics for the NJR-HES-PROMs linked dataset

| | Knee Replacement |
|---|---|
| Year of primary | |
| 2008 | 33504 (17.8%) |
| 2009 | 45928 (42.1%) |
| 2010 | 52460 (70.0%) |
| 2011 | 56617 (100.0%) |
| Age at primary knee replacement | 69.9 (SD 9.3) |
| Sex | |
| Female | 106812 (56.7%) |
| Male | 81697 (43.3%) |
| Body mass index | 30.9 (SD 5.5) |
| Index of Multiple Deprivation (IMD) quintiles | |
| Least deprived | 40247 (21.6%) |
| 2 | 42721 (22.9%) |
| 3 | 35839 (19.2%) |
| 4 | 34691 (18.6%) |
| Most deprived | 33119 (17.8%) |
| Rurality, at primary | |
| urban >=10,000 | 140202 (74.5%) |
| town and fringe | 22366 (11.9%) |
| village/isolated | 25618 (13.6%) |
| ethnicity | |
| white | 161079 (92.9%) |
| non-white | 12237 (7.1%) |
| Number comorbidities at primary (none) | |
| None | 141570 (75.1%) |
| Mild | 38083 (20.2%) |
| Moderate | 6875 (3.7%) |
| Severe | 1981 (1.1%) |
| ASA grade | |
| P1 - Fit and healthy | 20087 (10.7%) |
| P2 - Mild disease not incapacitating | 138997 (73.7%) |
| P3 - P5 | 29425 (15.6%) |
| Minimally invasive (no) | |
| No | 176683 (93.7%) |
| Yes | 11826 (6.3%) |
| Surgical volume per consultant | |
| <=10 operations | 5312 (2.8%) |
| 11-50 | 58102 (30.8%) |
| 51-75 | 42101 (22.3%) |
| 76-100 | 33558 (17.8%) |

| | |
|---|---|
| 101-150 | 32381 (17.2%) |
| >150 | 17055 (9.1%) |
| Surgeon experience | |
| <8 training years | 44650 (23.7%) |
| Consultant (≥8 training years) | 143859 (76.3%) |
| Surgical approach (knee) | |
| Lateral parapatellar | 1756 (0.9%) |
| Medial parapatellar | 175112 (92.9%) |
| Mid-Vastus | 5710 (3.0%) |
| Sub-Vastus | 2300 (1.2%) |
| Other | 3631 (1.9%) |
| Primary graft femur | |
| No | 186951 (99.2%) |
| Yes | 1558 (0.8%) |
| Implant fixation | |
| Cementless | 9429 (5.0%) |
| cemented | 177031 (94.0%) |
| Hybrid | 1947 (1.0%) |
| Primary graft tibia | |
| No | 187679 (99.6%) |
| Yes | 830 (0.4%) |
| Type of primary implant | |
| UKR | 13266 (7.0%) |
| TKR | 175243 (93.0%) |
| Type of mechanical thromboprophylaxis | |
| None | 16746 (8.9%) |
| Any | 171763 (91.1%) |
| Type of chemical thromboprophylaxis | |
| None | 13239 (7.0%) |
| Aspirin only | 16356 (8.7%) |
| LMWH (+/-Other) | 134648 (71.4%) |
| Other (no LMWH) | 24266 (12.9%) |
| Unit type | |
| Public hospital | 153780 (81.6%) |
| Independent sector - hospital | 24716 (13.1%) |
| Independent sector - treatment centre | 10013 (5.3%) |
| OKS, baseline score | 18.0 (SD 7.8) |
| EQ-5D Anxiety Depression | |
| I am not anxious or depressed | 65123 (62.4%) |
| I am moderately anxious or depressed | 32676 (31.3%) |
| I am extremely anxious or depressed | 3710 (3.6%) |

**Table C.** Descriptive statistics comparing patients in the full CPRD dataset to those with linked HES data for the CPRD-HES linked datasets

| **Year of primary (2010-2011)** | CPRD (n=37,906) | CPRD-HES (n=17,378) |
|---|---|---|
| | 3118 (8.2%) | 995 (5.7%) |
| | 10011 (26.4%) | 4486 (25.8%) |
| | 17920 (47.3%) | 8415 (48.4%) |
| | 6857 (18.1%) | 3482 (20.0%) |
| **Age at primary (continuous variable)** | | |
| | 69.6 (SD 9.7) | 69.4 (SD 9.2) |
| **Sex (Woman)** | | |
| | 16328 (43.1%) | 7415 (42.7%) |
| **Body mass index** | | |
| | 120 (0.4%) | 48 (0.4%) |
| | 5201 (18.0%) | 2204 (16.2%) |
| | 11427 (39.6%) | 5239 (38.6%) |
| | 7739 (26.8%) | 3777 (27.8%) |
| | 4364 (15.1%) | 2306 (17.0%) |
| **Index of Multiple Deprivation (IMD) quintiles, at primary** | | |
| | 7254 (24.5%) | 3890 (22.4%) |
| | 7111 (24.0%) | 4145 (23.9%) |
| | 6548 (22.1%) | 3918 (22.6%) |
| | 5031 (17.0%) | 3078 (17.7%) |
| | 3725 (12.6%) | 2328 (13.4%) |
| **Region** | | |
| | 2244 (5.9%) | 706 (4.1%) |
| | 4547 (12.0%) | 2003 (11.5%) |
| | 3436 (9.1%) | 1464 (8.4%) |
| | 1033 (2.7%) | 434 (2.5%) |
| | 5066 (13.4%) | 2551 (14.7%) |
| | 5537 (14.6%) | 2605 (15.0%) |
| | 4894 (12.9%) | 2144 (12.3%) |
| | 4738 (12.5%) | 2386 (13.7%) |
| | 4589 (12.1%) | 2320 (13.4%) |
| | 1822 (4.8%) | 765 (4.4%) |
| **Smoker** | | |
| | 10576 (33.4%) | 5122 (34.6%) |
| | 18106 (57.2%) | 8310 (56.2%) |
| | 2971 (9.4%) | 1368 (9.2%) |
| **Drink alcohol** | | |
| | 634 (2.5%) | 322 (2.7%) |
| | 4657 (18.2%) | 2242 (18.8%) |
| | 20367 (79.4%) | 9371 (78.5%) |
