# Supplementary material for: UK poSt Arthroplasty Follow-up rEcommendations (UK SAFE): what does analysis of linked, routinely collected national datasets tell us about mid–late term revision risk after knee replacement?
Source: BMJ Open. 2022 Mar 9;12(3):e046900. doi: 10.1136/bmjopen-2020-046900 (PMC8915365; doi:10.1136/bmjopen-2020-046900)
Supplement: Author's manuscript [file bmjopen-2020-046900.draft_revisions.pdf]

BMJ Open is committed to open peer review. As part of this commitment we make the peer review history of every article we publish publicly available.

When an article is published we post the peer reviewers' comments and the authors' responses online. We also post the versions of the paper that were used during peer review. These are the versions that the peer review comments apply to.

The versions of the paper that follow are the versions that were submitted during the peer review process. They are not the versions of record or the final published versions. They should not be cited or distributed as the published version of this manuscript.

BMJ Open is an open access journal and the full, final, typeset and author-corrected version of record of the manuscript is available on our site with no access controls, subscription charges or pay-per-view fees (http://bmjopen.bmj.com).

If you have any questions on BMJ Open's open peer review process please email info.bmjopen@bmj.com

UK poSt Arthroplasty Follow-up rEcommendations (UK SAFE): What does analysis of routine NHS data tell us about mid-late term revision risk after knee replacement?

Journal:	*BMJ Open*
Manuscript ID	bmjopen-2020-046900
Article Type:	Original research
Date Submitted by the Author:	17-Nov-2020
Complete List of Authors:	Smith, Lindsay; University of the West of England, Health & Applied Sciences<br>Garriga, Cesar; University of Oxford Nuffield Department of Orthopaedics Rheumatology and Musculoskeletal Sciences<br>Kingsbury, Sarah; Leeds Biomedical Research Centre; University of Leeds Leeds Institute of Rheumatic and Musculoskeletal Medicine<br>Pinedo-Villanueva, Rafael; University of Oxford Nuffield Department of Orthopaedics Rheumatology and Musculoskeletal Sciences<br>Delmestri, Antonella; University of Oxford Nuffield Department of Orthopaedics Rheumatology and Musculoskeletal Sciences<br>Arden, Nigel; University of Oxford, Nuffield Department of Orthopaedics, Rheumatology and Musculoskeletal Sciences (NDORMS); University of Southampton, MRC Lifecourse Epidemiology Unit<br>Stone, Martin; Leeds Teaching Hospitals NHS Trust<br>Conaghan, Philip; University of Leeds Leeds Institute of Rheumatic and Musculoskeletal Medicine<br>Judge, Andrew; University of Bristol, Translational Health Sciences, Bristol Medical School
Keywords:	SURGERY, Adult orthopaedics < ORTHOPAEDIC & TRAUMA SURGERY, Knee < ORTHOPAEDIC & TRAUMA SURGERY, Musculoskeletal disorders < ORTHOPAEDIC & TRAUMA SURGERY

SCHOLARONE™
Manuscripts

I, the Submitting Author has the right to grant and does grant on behalf of all authors of the Work (as defined in the below author licence), an exclusive licence and/or a non-exclusive licence for contributions from authors who are: i) UK Crown employees; ii) where BMJ has agreed a CC-BY licence shall apply, and/or iii) in accordance with the terms applicable for US Federal Government officers or employees acting as part of their official duties; on a worldwide, perpetual, irrevocable, royalty-free basis to BMJ Publishing Group Ltd ("BMJ") its licensees and where the relevant Journal is co-owned by BMJ to the co-owners of the Journal, to publish the Work in this journal and any other BMJ products and to exploit all rights, as set out in our licence.

The Submitting Author accepts and understands that any supply made under these terms is made by BMJ to the Submitting Author unless you are acting as an employee on behalf of your employer or a postgraduate student of an affiliated institution which is paying any applicable article publishing charge ("APC") for Open Access articles. Where the Submitting Author wishes to make the Work available on an Open Access basis (and intends to pay the relevant APC), the terms of reuse of such Open Access shall be governed by a Creative Commons licence – details of these licences and which Creative Commons licence will apply to this Work are set out in our licence referred to above.

Other than as permitted in any relevant BMJ Author's Self Archiving Policies, I confirm this Work has not been accepted for publication elsewhere, is not being considered for publication elsewhere and does not duplicate material already published. I confirm all authors consent to publication of this Work and authorise the granting of this licence.

For peer review only

UK poSt Arthroplasty Follow-up rEcommendations (UK SAFE): What does analysis of routine NHS data tell us about mid-late term revision risk after knee replacement?

Authors:

Smith, L.K.; Garriga, C.; Kingsbury, S.R.; Pinedo-Villanueva, R.; Delmestri, A.; Arden, N.; Stone, M.; Conaghan, P.G; Judge, A.;

Corresponding author:

Lindsay K. Smith

Postal address: University of the West of England, Faculty of Health & Applied Sciences, Rm 2G40 Glenside Campus, Blackberry Hill, Bristol BS16 1DD, UK

Email: Lindsay.smith2@nhs.net

Mobile no: 07979 007 625

Fax no: not available.

Author affiliations:

Cesar Garriga: Nuffield Department of Orthopaedics, Rheumatology and Musculoskeletal Sciences, Nuffield Orthopaedic Centre, University of Oxford, UK

Sarah Kingsbury: Leeds Institute of Rheumatic and Musculoskeletal Medicine, University of Leeds, and NIHR Leeds Biomedical Research Centre, Leeds, UK

Rafael Pinedo-Villanueva: Nuffield Department of Orthopaedics, Rheumatology and Musculoskeletal Sciences, Nuffield Orthopaedic Centre, University of Oxford, UK

Antonella Delmestri: Nuffield Department of Orthopaedics, Rheumatology and Musculoskeletal Sciences, Nuffield Orthopaedic Centre, University of Oxford, UK

Nigel Arden: Nuffield Department of Orthopaedics, Rheumatology and Musculoskeletal Sciences, Nuffield Orthopaedic Centre, University of Oxford, UK

Martin Stone: Leeds Teaching Hospitals NHS Trust, Leeds UK and NIHR Leeds Biomedical Research Centre, Leeds, UK

Philip Conaghan: Leeds Institute of Rheumatic and Musculoskeletal Medicine, University of Leeds, and NIHR Leeds Biomedical Research Centre, Leeds, UK

Andrew Judge: Translational Health Sciences, Bristol Medical School, University of Bristol, UK

Word count: 3909

Keywords: knee replacement, mid-late revision, follow-up, Cox regression

For peer review only

Abstract (293 words)

Objective. To identify patients at risk of mid-late term revision of knee replacement (KR) to inform targeted follow-up.

Design: Analysis of linked national datasets from primary and secondary care (Clinical Practice Research Datalink [CPRD-GOLD]; National Joint Registry [NJR]; English Hospital Episode Statistics [HES]; Patient Reported Outcome Measures [PROMs]).

Participants: Primary elective KRs aged ≥18.

Event of interest: Revision surgery ≥5 years (mid-late term) after primary KR.

Statistical methods: Cox regression modelling to ascertain risk factors of mid-late term revision. Hazard ratios (HR) and 95% confidence intervals (CI) assessed association of sociodemographic factors, comorbidities, medication, surgical variables and PROMs with mid-late term revision.

Results: NJR-HES-PROMs data was available from 2008-2011 on 188,509 KR. CPRD GOLD-HES data covered 1995-2011 on 17,378 KR. Patients had minimum 5-years post primary surgery to end 2016. Age and gender distribution were similar across datasets; mean age 70 years, 57% female. In NJR there were 8,607 (4.6%) revisions, median time-to-revision after primary surgery 1.8 years (range 0-8.8), with 1,055 (0.6%) mid-late term revisions; in CPRD GOLD, 877 (5.1%) revisions, median time-to-revision 4.2 years (range 0.02-18.3), with 352 (2.0%) mid-late term revisions.

Reduced risk of revision after 5 years was associated with older age (HR:0.95; 95% CI: 0.95-0.96), obesity (0.78; 0.64- 0.96), living in deprived areas (0.76; 0.62-0.93), non-white ethnicity (0.63; 0.47-0.84), less pain and functional limitation (0.29; 0.23-0.36) or moderate anxiety/depression (0.80; 0.70-0.92) at primary surgery.

Increased risk of revision after 5 years was associated with male gender (1.32; 1.04-1.67), and when anticonvulsants (gabapentin, pregabalin) (1.58; 1.01-2.47) or opioids (1.36; 1.08-1.71) were required prior to primary surgery.

No implant factors were identified.

Conclusion: The risk of mid-late term KR revision is very low. Increased risk of revision is associated with patient case-mix factors and there is evidence of sociodemographic inequality.

Article summary

- This study is part of a wider programme of work to identify potential patient groups for follow-up after hip and knee replacement and used large national routine datasets from primary and secondary care
- The linkage of datasets allowed us to explore the impact of multiple risk factors on the mid-late term risk of revision of knee replacement
- This is one of the first studies to identify predictors of mid-late term revision risk for knee replacement from real-world data and contributes to the discussion on follow-up.

For peer review only - http://bmjopen.bmj.com/site/about/guidelines.xhtml

INTRODUCTION

Primary knee replacement (KR) surgery is a common elective orthopaedic procedure for the treatment of knee pain due to end stage osteoarthritis (OA). There is good evidence showing that KR is highly clinically effective, reducing symptoms of pain and functional limitations for the vast majority of patients[1-3] and is also cost effective[4,5]. Over 100,000 operations are carried out each year in the UK[6]. The lifetime risk of receiving knee arthroplasty in the UK is estimated to be 10.8% for women and 8.1% for men[7]. These numbers are projected to increase with an ageing and increasingly obese population, placing a growing public health burden on the NHS in respect of funding and capacity[8].

There is significant pressure on hospital trusts to reduce the amount of follow-up appointments due to expanding waiting lists, cancellation of elective surgery and increasing numbers of patients needing primary joint replacement. Although previous British Orthopaedic Association guidelines recommended outpatient follow-up at 1 and 7 years, and every 3 years thereafter, recent guidelines for primary joint replacement in the UK recommend further research on follow-up due to a lack of evidence[9,10]. There is variation across the country in how hospitals organise follow-up services and many units stopped follow-up after an early post-operative check[11]. Evidence is required on the impact that disinvestment in follow-up services may have on patient safety. There is a need to ensure early detection of patients with failing implants and target follow-up accordingly. In March 2014, the James Lind Alliance and NIHR Priority Setting Partnership for Hip and Knee Replacement for Osteoarthritis identified that defining the ideal postoperative follow-up period and the best long-term care model for people with OA and knee replacement was amongst its top ten research priorities, highlighting the importance of appropriate follow-up to ensure the health of patients.

The objective of this study was to use nationally available datasets to identify which groups of patients with KR may require follow-up based on their mid-late term revision risk (5 or more years post primary surgery). This work forms part of a larger programme of work, UK SAFE, that was designed to address the research question: Is it safe to disinvest in mid-late term follow-up of hip and knee replacement?[12] The UK SAFE programme of work took place between 01/12/2016 and 30/11/2020.

For peer review only

For peer review only - http://bmjopen.bmj.com/site/about/guidelines.xhtml

METHODS

Study design

This was a nation-wide retrospective cohort study in which national data from primary care (Clinical Practice Research Datalink) and secondary care (National Joint Registry, Hospital Episode Statistics and Patient-Reported Outcome Measures) were linked to identify predictors of mid-late term revision of KR.

Sources of data

(1) CPRD GOLD-HES: The Clinical Practice Research Datalink (CPRD) GOLD comprises the entire computerised medical records of a sample of patients attending general practitioners (GPs) in the UK[13]. It contains information on over 14 million patients registered at over 700 general practices in the UK that are representative of the population in terms of demographics such as age and sex[14]. GPs in the UK play a key role in the delivery of healthcare by providing primary care and referral to specialist hospital services, and each GP practice records this medical information for individual patients. The CPRD is administered by the Medicines and Healthcare products Regulatory Agency (MHRA). CPRD GOLD records contain all clinical and referral events in both primary and secondary care in addition to comprehensive demographic information, prescription data, and hospital admissions. Data is stored using Read codes for diseases that are cross-referenced to the International Classification of Diseases (ICD-10). Read codes are used as the standard clinical terminology system within UK primary care. Only practices that pass quality control are used as part of CPRD GOLD. CPRD ensures patient confidentiality by providing anonymised healthcare records.

CPRD GOLD data were linked to data for all-cause mortality, provided by the Office for National Statistics (ONS)[15]. CPRD GOLD data were also linked to the Index of Multiple Deprivation (IMD) and to the Hospital Episode Statistics (HES) database (described later). CPRD already provide access to HES data for England that is held under the CPRD data Linkage Scheme, available for around 60% of patients in the CPRD GOLD database. Previous research by the CPRD team has shown that linked practices/patients are representative of the CPRD GOLD population as a whole[14].

(2) NJR-HES-PROMs: Starting in 2003, the National Joint Registry (NJR) collected information on all hip and knee replacements performed each year in both public and private hospitals in England, Wales, Northern Ireland and the Isle of Man[16]. Data are entered into the NJR using forms completed at the time of surgery, and revision operations are linked to primaries using unique patient identifiers. Data recorded in the NJR includes prosthesis and

For peer review only - http://bmjopen.bmj.com/site/about/guidelines.xhtml

operative information (prosthesis type, approach, thromboprophylaxis); patient information (age, gender, body mass index (BMI), American Society of Anaesthesiologists (ASA) grade); surgeon and unit information (including caseloads, public/private status).

The HES database holds information on all patients admitted to National Health Service (NHS) hospitals in England, including diagnostic ICD codes providing information about a patient's illness or condition and NHS national clinical procedural codes (OPCS4) for surgery. It covers a smaller geographical area than the NJR and does not include privately funded operations. However, HES provides additional information for every patient (including detailed comorbidity information and deprivation indices) and about every procedure (including length of stay and need for blood transfusion or critical care). Additional records contain details of readmissions, reoperations, and revisions not recorded in the NJR database.

Since April 2009, Patient-Reported Outcome Measure data (PROMs) have been collected on all knee replacements performed in public hospitals in England[17]. A health related quality of life questionnaire (the EuroQol five domain (EQ-5D-3L)[18]) and a joint-specific outcome score (the Oxford Knee Score (OKS)[19]) are collected pre-operatively and at 6 months after surgery, along with patient-reported measures of preoperative disability and postoperative satisfaction.

For this analysis we used NJR records linked to data from the HES and PROMs databases on all KR operations.

Participants

Anonymised records were extracted for all patients over 18 years of age receiving primary knee replacement surgery. For CPRD GOLD-HES data, the time span covered the years 1995 to 2017; for NJR-HES-PROMS data, it covered the years 2009 to 2017. Patients were included if they had primary total knee replacement or uni-compartmental knee replacement. We excluded patients that had revision surgery and total joint replacement of unspecified fixation. The following exclusions were made to remove potential case-mix issues: other injuries due to trauma, such as transport accidents and falls; non-elective admissions; a diagnosis other than primary knee OA.

Primary outcome

The primary outcome was defined as mid-late term revision (defined as more than 5 years post primary surgery). Revision is defined as the removal, exchange or addition of any of the

For peer review only - http://bmjopen.bmj.com/site/about/guidelines.xhtml

components of arthroplasty. In the NJR-HES-PROMS linked datasets, operative details are completed using the NJR dataset, rather than the OPCS4 coding used by the HES dataset. The NJR collects operative data using two forms, one for primary operations, and the other for revision operations. In both cases, all component labels from the surgery are attached to the form and it is from these that the component details are collected. Revision operations are linked to primaries using unique patient identifiers and so, two operations on the same knee would be linked using this system. The combination of the separate coding at source and the secondary linkage gives confidence that primary and revision operations are correctly identified. In the CPRD GOLD dataset, subjects with a revision surgery procedure are identified using the Read codes, and for those with HES-linked data OPCS4 codes can be used.

Predictors

Secondary Care Predictors: The patient level characteristics available in NJR and HES include: age, gender, BMI, area deprivation, rurality, ethnicity, Charlson co-morbidity index[20] (calculated from HES using ICD10 codes), ASA grade. Data from the NJR provide additional information on surgical and operative factors: whether or not a minimally invasive technique was used; annual surgeon volume/case load, operative time, grade of operating surgeon, surgical approach, patient position, implant fixation, type of mechanical or chemical thromboprophylaxis, unit type (public, private, independent sector treatment centre). Data from the PROMs database provides additional information on symptoms of pain, function and health related quality of life pre-operatively and at 6-months post-surgery. Pain and function are measured using the OKS. The EQ-5D-3L consists of five questions (assessing mobility, self-care, ability to conduct usual activities, degree of pain/discomfort, and degree of anxiety/depression), ranging from 1 (best state) to 3 (worst state). EQ-5D-3L can be expressed as an overall index (graded from -0.594 to 1), or as ordinal responses for each category

Primary Care Predictors: The CPRD GOLD database includes information on: age, gender, BMI, joint replaced (hip/knee), year of joint replacement operation, recorded diagnosis of OA (yes/no), fracture pre-surgery (yes/no), calcium and vitamin D supplements, use of bisphosphonates, use of selective oestrogen receptor modulators, oral glucocorticosteroid therapy, smoking status and alcohol intake recorded closest to the date of the primary surgery, , region of UK, co-morbid conditions registered by the physician from the following list (asthma, malabsorptive syndromes, inflammatory bowel disease, hypertension, hyperlipidaemia, ischaemic heart disease, stroke, atioa (COPD), chronic kidney failure, neoplasms, diabetes), use of drugs which can affect fracture risk (proton pump inhibitors,

For peer review only - http://bmjopen.bmj.com/site/about/guidelines.xhtml

anti-arrhythmics, anticonvulsants, antidepressants, anti-Parkinson drugs, statins, thiazide diuretics, anxiolytics).

Sample size

We included all patients receiving planned elective primary surgery for knee OA. For the NJR-HES-PROMs data, this covered the years 2009 to 2016 (as our requested linked HES data was from 2008 onwards and earlier years of data were not available to us). For the CPRD GOLD-HES this spanned the years 1995 to 2016. For both datasets we excluded patients receiving a primary knee replacement after 2011 to ensure all patients had at least 5-years follow-up, as we were not interested in revisions occurring in the early period up to 5-years after the primary replacement surgery. The sample was created from all available data that satisfied these criteria.

Statistical analysis methods

Survival analysis was used to model time to revision. To identify patients most likely to require revision, proportional hazards regression modelling was used to identify pre, peri- and post-operative predictors of mid-late term revision. The date of the first incidence of a subject's knee replacement was used as the start time. The event of interest in all time-to-event models was the first recorded revision operation. Linearity of continuous predictors was assessed using fractional polynomial regression modelling. Proportionality assumptions were checked using Schoenfeld residuals. Missing data was handled by using multiple imputation methods using the ICE (Imputation by Chained Equations) procedure[21]. Standard errors were calculated using Rubin's Rules. We include all predictor variables in the multiple imputation process, together with the outcome variable (Nelson Aalen estimate of survival time and whether or not the patient had the outcome) as this carried information about missing values of the predictors.

For the CPRD GOLD-HES primary care we generated 10 imputed datasets for KR. Data was imputed for the variables BMI, deprivation index, smoking and drinking risk factors. For secondary care NJR-HES-PROMS dataset we generated a single imputed dataset for KR. Variables imputed were BMI, deprivation index, rurality, ethnicity, OKS baseline scores and EQ-5D-3L item for anxiety and depression. We ran univariate Cox regression models. Risk factors with a P value<0.20 were selected for a multivariable model. Backward selection of variables was used to identify variables to keep in the final model risk factors with at least one category with a P value<0.05. For the CPRD GOLD-HES primary care dataset we present two final models, one with medication use as yes/no variables, and the other model with daily defined doses (DDDs) calculated from one year prior to the primary surgery and

For peer review only - http://bmjopen.bmj.com/site/about/guidelines.xhtml

divided in tertiles.

Patient and Public Involvement

Members of the National Institute for Health Research (NIHR) Leeds Biomedical Research Centre and Bristol patient and public involvement groups (PPI)were involved in developing the UK SAFE research question and work programme based on experiences of arthroplasty and preferences for care. The steering committee includes a PPI co-applicant who has contributed to interpretation of the results and will be involved in production of the final report that is disseminated to the public, patients and NHS staff.

RESULTS

Participants

For the CPRD GOLD-HES dataset, 64,071 sets of data were available, and Table 1 shows the steps toward 17,378 participants. Construction of the NJR-HES-PROMs dataset commenced with 841,212 records in the NJR and 108,509 participants after exclusions (Table 2).

Table 1. Diagram showing selection of patients for inclusion in study: Primary care data.

**Included**		**Excluded**
Patients with primary knee replacement in CPRD GOLD (64,071)		
	→	Outcome (knee revision) and index event (knee replacement) not collected in Northern Ireland (1402; 2.2%), Scotland (5397; 8.4%), Wales (6,982; 10.9%)
	→	Outcome (knee revision) and index event (knee replacement) not available (27, 454; 42.9%)
Patients with primary knee replacement in CPRD GOLD linked to HES and used in survival analysis (22,836)		
	→	Primary surgery after 2011 (allowing for 5 years of follow up) (5,458; 23.9%)
Patient with primary knee replacement used in the survival analysis (17,378)		

Key: CPRD - Clinical Practice Research Datalink; HES – Hospital Episode Statistics

For peer review only - http://bmjopen.bmj.com/site/about/guidelines.xhtml

Table 2. Diagram showing selection of patients for inclusion in study: Secondary care data

**Included**		**Excluded**
Patients with primary knee replacement in National Joint Registry (841,212)		
	→	Primary surgery before 2008 (no data available in HES) (169,776; 20.2%)
	→	Primary surgery after 2011 (allowing for 5 years of follow-up) (414,832; 49.4%)
	→	Without primary surgery date (1,037; 0.1%)
	→	A diagnosis other than primary knee osteoarthritis (2,940; 0.4%)
	→	Non-elective surgeries (535; 0.06%)
	→	Without information on type of admission (63,416; 7.5%)
Patient with primary knee replacement used in the survival analysis (108, 509)		

Summary statistics for patients in the CPRD GOLD-HES and the NJR-HES-PROMs linked datasets are provided (Supplementary file I: Tables A & B). The CPRD GOLD-HES linked data covered a longer time period between 1995 to 2011; the NJR-HES-PROMs data were available 2009 to 2011. Both datasets allowed a minimum of 5-years follow-up to end 2016. The age and gender distribution of patients was similar across both datasets, with a mean age of 70 at time of knee replacement and 57% female. An extensive range of patient case-mix, surgical, operative factors and primary care prescribing data was available for analysis.

The CPRD GOLD-HES dataset had a longer time-to-revision. There were 877 (5.1%) revisions, with median time-to-revision of 4.2 years (range 0.02 to 18.3 years) and 352 (2.0%) were mid-late term revisions.

In the NJR-HES-PROMs data, there were 8,607 (4.6%) knee replacement revisions with a median time-to-revision of 1.8 years (range 0 to 8.8 years); this included 1,055 (0.6%) mid-late term revisions.

Predictors of mid-late term revision

<u>Patient demographics</u>

Older age at the time of primary KR was associated with a lower risk of mid-late revision (Tables 3, 4). The effect of age was linear and the association was strong where, for a 1-

For peer review only - http://bmjopen.bmj.com/site/about/guidelines.xhtml

For peer review only

year increase in age at surgery, the risk of outcome reduced by 5%, and this finding was consistent across the CPRD GOLD-HES and NJR-HES-PROMs datasets. The effect of gender was that males had an increased risk of mid-late revision compared to females. This was only observed in the CPRD GOLD-HES data, where males had a 32% increased risk of revision, while the effect size was weaker and non-significant in the NJR-HES-PROMs dataset.

The effect of obesity on outcome was demonstrated in the NJR-HES-PROMs dataset, where compared to those of normal BMI, underweight patients were at increased risk of revision, and obese patients at reduced risk of mid-late revision. The effect of IMD deprivation in the NJR-HES-PROMs dataset showed that patients in the most deprived areas were less likely to receive mid-late term revision; there was no such association with obesity or deprivation observed in the CPRD GOLD-HES dataset. An association with ethnicity was observed only in the NJR-HES-PROMs dataset, where patients of non-white ethnicity were less likely to be revised mid-late term.

Implant factors (NJR-HES-PROMs dataset)

None of the implant related factors were associated with an increased mid-late revision risk.

Pre-operative PROMs (Table 4)

There was a clear linear trend with the pre-operative OKS, where patients with the least pain and functional limitations at the time of surgery were substantially more likely to require mid-late term revision. Patients with pre-operative anxiety/depression were found to be less likely to receive a mid-late revision operation.

Primary care co-morbidities and medication use (Table 3)

Through the CPRD GOLD-HES dataset, we were able to investigate co-morbidities recorded prior to surgery and medication use. There was no effect of pre-operative co-morbidity for KR. With medication use, oral glucocorticoid steroid therapy was associated with a lower risk of revision whereas use of antiarrhythmics and anticonvulsants placed patients at a higher risk.

For the pain medication use, an increased revision risk was observed in those patients requiring opiates. When examining effects of medication use in more detail, by looking at DDDs calculated from the 1 year prior to the primary surgery and divided into tertiles, the effect of opioids was only significant in the highest DDD tertile of >600 DDD.

For peer review only - http://bmjopen.bmj.com/site/about/guidelines.xhtml

Table 3. Cox regression model identifying risk factors for revision after 5 years of primary total knee and unicompartmental replacement. Primary care data.

**Risk factors (reference category)**	**Patients undergoing KR (N=17,378)**		**Patients undergoing KR with missing dose for bisphosphonates & opioids excluded (N=14,470)**
	**Crude analysis Hazard ratio [95% CI]; P-value**	**Adjusted analysis (Drug yes/no) Hazard ratio [95% CI]; P-value**	**Adjusted analysis (Drug DDD) Hazard ratio [95% CI]; P-value**
**Year of primary KR** (2010-2011)			
1995-1999	4.63 [1.98 to 10.81]; **P<0.01**	5.39 [2.28 to 12.75]; **P<0.01**	8.10 [2.52 to 25.98]; **P<0.01**
2000-2004	3.24 [1.42 to 7.41]; **P=0.01**	3.65 [1.59 to 8.40]; **P<0.01**	5.49 [1.73 to 17.37]; **P<0.01**
2005-2009	2.36 [1.04 to 5.36]; **P=0.04**	2.42 [1.06 to 5.52]; **P=0.04**	3.45 [1.10 to 10.86]; **P=0.03**
**Age at primary KR** (continuous variable)	0.93 [0.92 to 0.94]; **P<0.01**	0.93 [0.92 to 0.94]; **P<0.01**	0.93 [0.92 to 0.94]; **P<0.01**
**Sex** (Woman)			
Man	1.26 [1.02 to 1.55]; **P=0.03**	1.24 [1.00 to 1.53]; P=0.06	1.32 [1.04 to 1.67]; P=**0.02**
**Body mass index** (normal)			
Underweight			
Overweight	1.02 [0.71 to 1.45]; P=0.93	0.97 [0.67 to 1.42]; P=0.89	0.98 [0.65 to 1.46]; P=0.91
Obese Class I (Moderately obese)	1.25 [0.86 to 1.80]; P=0.24	1.06 [0.71 to 1.57]; P=0.79	1.09 [0.69 to 1.70]; P=0.72
Obese Class II and higher	1.35 [0.91 to 2.00]; P=0.14	1.03 [0.65 to 1.63]; P=0.90	0.97 [0.58 to 1.63]; P=0.90
**Region** (East Midlands)			
East of England	0.83 [0.49 to 1.41]; P=0.49	0.95 [0.56 to 1.61]; P=0.84	
London	0.81 [0.46 to 1.43]; P=0.47	0.96 [0.54 to 1.71]; P=0.90	
North East	0.28 [0.08 to 0.95]; **P=0.04**	0.27 [0.08 to 0.91]; **P=0.04**	

For peer review only - http://bmjopen.bmj.com/site/about/guidelines.xhtml
For peer review only

North West	0.88 [0.53 to 1.47]; P=0.63	0.93 [0.56 to 1.55]; P=0.78	
South Central	0.81 [0.48 to 1.36]; P=0.42	0.93 [0.55 to 1.57]; P=0.79	
South East Coast	1.08 [0.64 to 1.82]; P=0.77	1.37 [0.82 to 2.29]; P=0.23	
South West	0.86 [0.51 to 1.44]; P=0.56	1.01 [0.60 to 1.70]; P=0.97	
West Midlands	0.74 [0.44 to 1.26]; P=0.26	0.79 [0.46 to 1.33]; P=0.37	
North East	0.87 [0.46 to 1.65]; P=0.68	0.88 [0.47 to 1.66]; P=0.70	
**Drugs prior to primary KR**			
Oral glucocorticosteroid therapy	0.75 [0.56 to 1.02]; P=0.07	0.72 [0.53 to 0.99]; **P=0.04**	
**Drugs which can affect fracture risk prior to primary KR**			
Anti-arrhythmics	1.35 [0.97 to 1.87]; P=0.08	1.41 [1.00 to 1.98]; **P=0.05**	
Anticonvulsants	1.72 [1.11 to 2.68]; **P=0.02**	1.58 [1.01 to 2.47]; **P=0.04**	
**Painkillers/anti-inflammatory drugs**			
Total opiates	1.40 [1.13 to 1.73]; **P<0.01**	1.36 [1.08 to 1.71]; **P=0.01**	
**DDDs 1 year prior to primary KR**			
**Bisphosphonates** (no dose)			
<140 DDD	0.25 [0.03 to 1.79]; P=0.17		0.40 [0.06 to 2.91]; P=0.37
>=140 to 340 DDD	1.47 [0.73 to 2.96]; P=0.28		2.44 [1.12 to 5.36]; **P=0.03**
>340 DDD	0.55 [0.14 to 2.21]; P=0.40		1.08 [0.26 to 4.54]; P=0.92
Dose missing	1.23 [0.51 to 2.95]; P=0.65		
**Opioids total** (no dose)			
<85 DDD	1.45 [0.95 to 2.21]; P=0.09		1.33 [0.86 to 2.06]; P=0.20

For peer review only

>=85 to 365 DDD	1.36 [0.97 to 1.90]; P=0.07	1.27 [0.90 to 1.79]; P=0.17
>365 DDD	1.85 [1.20 to 2.85]; **P=0.01**	1.67 [1.08 to 2.59]; **P=0.02**
Dose missing	1.28 [0.95 to 1.72]; P=0.10	

KR, total and unicompartmental knee replacement; DDD, daily defined dose; CI, confidence interval. Hazard ratio represents number of times to have a revision after 5 years compared with the reference group. A value >1 indicates that the group has higher risk for revision.

Variables included in the final regression model are those with at least one category with a P-value<0.05 for the 10 imputed datasets in a backward selection.

Note: Body mass index and sex were force-entered into all models. "Total opiates" includes benzomorphan derivatives, diphenylpropylamine derivatives, morphinan derivatives, natural opium alkaloids, oripavine derivatives, phenylpiperidine derivatives, and other opioids.

Table 4. Cox regression model identifying risk factors of revision after 5 years of primary total knee and unicompartmental replacement. Hospital data.

	**Patients undergoing KR (N=188,509)**	
**Risk factors (reference category)**	**Crude analysis Hazard ratio [95% CI]; P-value**	**Adjusted analysis (Drug yes/no) Hazard ratio [95% CI]; P-value**
**Year of primary KR** (2008)		
2009	0.91 [0.78 to 1.06]; P=0.23	0.89 [0.76 to 1.04]; P=0.13
2010	0.82 [0.68 to 0.98]; **P=0.03**	0.80 [0.67 to 0.97]; **P=0.02**
2011	0.83 [0.64 to 1.07]; P=0.15	0.81 [0.63 to 1.05]; P=0.11
**Age at primary KR** (continuous variable)	0.94 [0.9-0.9]; **P<0.01**	0.95 [0.9-1.0]; **P<0.01**
**Sex** (Women)		
Men	1.08 [1.0-1.2]; P=0.23	1.11 [1.0-1.3]; P=0.11
**Body mass index** (normal)		
Underweight	1.96 [0.96 to 4.01]; P=0.07	2.29 [1.12 to 4.68]; **P=0.02**
Overweight	1.04 [0.85 to 1.28]; P=0.68	0.93 [0.76 to 1.14]; P=0.49

For peer review only - http://bmjopen.bmj.com/site/about/guidelines.xhtml

Obese Class I (Moderately obese)	1.02 [0.83 to 1.25]; P=0.87	0.78 [0.64 to 0.96]; **P=0.02**
Obese Class II and higher	1.20 [0.96 to 1.49]; P=0.10	0.77 [0.61 to 0.96]; **P=0.02**
**IMD (quintiles), at primary KR** (less deprived 20%)		
Less deprived 20-40%	0.87 [0.72 to 1.05]; P=0.14	0.84 [0.70 to 1.01]; P=0.06
Less deprived 40-60%	0.91 [0.75 to 1.10]; P=0.32	0.81 [0.67 to 0.98]; **P=0.03**
More deprived 20-40%	0.94 [0.78 to 1.14]; P=0.55	0.83 [0.68 to 1.00]; P=0.06
Most deprived 20%	0.87 [0.71 to 1.06]; P=0.17	0.76 [0.62 to 0.93]; **P=0.01**
**Ethnicity** (white)		
Non-white	0.68 [0.5-0.9]; **P=0.01**	0.63 [0.5-0.8]; **P<0.01**
**OKS, baseline score** (0 to 10 points)		
(11 to 14 points)	0.82 [0.7-1.0]; **P=0.03**	0.78 [0.7-0.9]; **P=0.01**
(15 to 19 points)	0.69 [0.6-0.8]; **P<0.01**	0.64 [0.5-0.8]; **P<0.01**
(20 to 24 points)	0.51 [0.4-0.6]; **P<0.01**	0.44 [0.4-0.5]; **P<0.01**
(25 to 48 points)	0.37 [0.3-0.5]; **P<0.01**	0.29 [0.2-0.4]; **P<0.01**
**EQ-5D-3L Anxiety Depression, 3 months or closer to primary KR** (I am not anxious or depressed)		
I am moderately anxious or depressed	1.02 [0.9-1.2]; P=0.78	0.80 [0.7-0.9]; **P<0.01**
I am extremely anxious or depressed	1.26 [0.9-1.7]; P=0.14	0.78 [0.6-1.1]; P=0.11

Key: KR, total and unicompartmental knee replacement; IMD, Index of Multiple Deprivation; CI, confidence interval; OKS, Oxford Knee Score.

Hazard ratio represents number of times to have a revision after 5 years compared with the reference group. A value >1 indicates that the group has higher risk for revision.

Variables included in the final regression model are those with at least one category with a P-value<0.05 for a single imputed dataset in a backward selection.

Note: Body mass index and sex were force-entered into all models.

For peer review only

For peer review only - http://bmjopen.bmj.com/site/about/guidelines.xhtml

DISCUSSION

The risk of a mid-late revision operation (≥5-years) after primary knee replacement surgery is very low. Within our CPRD GOLD-HES primary care dataset, we had up to 20-years patient follow-up from the start point of 5-years after the primary operation and even then, the mid-late revision rate was only 2.0%. In this study, it was the patient case-mix factors that were associated with mid-late term revision surgery. Patients at increased risk were those who were younger, male gender, not obese, living in affluent areas, of white ethnicity, not anxious or depressed, and with very little pain and functional limitation at the time of primary surgery.

Strengths of this study include the use of large national routine datasets where the NJR data are mandatory and have near complete coverage, and the CPRD GOLD data is nationally representative in respect of UK population demographic characteristics. Large sample sizes afforded us the ability to identify predictors of a rare long-term outcome such as revision surgery. A limitation of the NJR-HES-PROMs linked data was limited long-term follow-up due to including data from 2009 onwards but only primary operations up to 2011 to allow for revision rates after 5-years. This was to allow us to explore the impact of pre-operative PROMs data which has only been collected since 2009. Strengths of NJR data are detailed surgical and hospital factors available in the data. A limitation is that there have been changes in anaesthesia and surgical techniques over time that may no longer reflect current orthopaedic practice. A strength of our CPRD GOLD dataset was over 20-years of follow-up, and the ability to capture a wide range of primary and hospital factors There were missing data for some of the variables in our data and this required us to use imputation to account for this in our analyses.

One of the aims of our study was to provide an evidence base for any group of patients in need of routine follow-up after KR. Our findings were consistent with a previous study using data from the CPRD GOLD in which the authors demonstrated an instantaneous risk of revision (risk of revision following a given period of implant survival) by age and gender subgroups[22]. The smoothed hazard plots consistently showed higher revision risks for men and younger patients at all timepoints. These graphs also showed that the trends in time to revision surgery were similar across all age bands, except for the most elderly patient groups in whom follow-up is limited by life expectancy. Males and younger patients were at a consistently higher revision risk over the whole follow-up, and these factors did not influence timing of when revision occurred. In our previous work, we have shown that younger age, males and obesity are risk factors for revision hip and knee replacement[23]. Our finding in respect of age is consistent with this existing literature, as is the effect of males, showing

For peer review only - http://bmjopen.bmj.com/site/about/guidelines.xhtml
For peer review only

that these effects are also seen in mid-late term. For obesity, the opposite effect was seen in the present study where this now had a protective effect on risk of mid-late revision, although the cause of this effect was not clear. With regard to deprivation, it has previously been shown that those in the most deprived areas are less likely to receive revision knee replacement surgery,[24] and this is disappointingly consistent with what we observed and may reflect inequalities in access to revision surgery. However, the effect of deprivation and that of obesity were only present in the secondary care dataset, which requires further investigation.

There have been previous studies looking at the effects of medication use on revision risk, particularly for medications associated with bone and fracture risk. It has been suggested that post-operative statin use reduces revision risk for hip replacement[25]. The effects seen here in our study showed that, in crude unadjusted analyses, statins reduced the risk for knee replacement revision, but this was attenuated in the full regression model, which may be explained by the association of statin use with obesity. Bisphosphonate use has also been suggested to reduce revision risk,[26] but we saw an opposite effect for high DDD users. They had increased revision risk, which may be associated with the reason for revision as Danish studies have shown that, although bisphosphonates reduced overall all-cause revision, the risk of revision for infection was increased[27].

The findings of this study suggest that patients receiving a mid-late revision surgery are a healthier, affluent group of patients of white ethnicity. It is unclear to what extent this represents need for revision surgery as this group may be more active, healthier, with lifestyle effects; or, is this a reflection of the known measurement error in using revision surgery as an outcome measure for the success of surgery? This patient group may simply be better able to navigate the care pathway (as for the primary operation), or reflect biases in patient-surgeon decision making, and may not be representative of those requiring revision surgery. There will always be patients in pain and functional difficulty that do not seek help from their GP or surgeon. It is of major interest to better understand why patient demographic characteristics seem to play a role in knee revision surgery.

The findings in respect of pre-operative pain medication use is of interest, given the only other risk factors of interest were healthy patient case-mix selection effects that are unlikely to be informative for extended follow-up. Use of oral glucocorticoid steroid therapy may be a surrogate marker for chronic health conditions and was associated with a lower risk of revision in our data. This may reflect reduced functional goals or expectations in this patient group, with less likelihood of proceeding to revision surgery, or a reluctance to proceed with

For peer review only - http://bmjopen.bmj.com/site/about/guidelines.xhtml

surgery due to an increased risk of infection. Anticonvulsants (gabapentin, pregabalin) and opioid use pre-operatively were associated with an increased mid-late revision risk. Although opioids may be recommended for controlling pain due to osteoarthritis before primary surgery,[28] they may also be indicative of chronic pain and/or opioid related comorbidities, and 2/3rds of patients have been shown to continue to use opioids post-surgery[29]. This group of patients often experience a mixed picture of pain and may have high levels of dissatisfaction after surgery, leading them to seek further surgical solutions for persistent pain[30,31]. Use of anticonvulsants prior to primary surgery is suggestive of existing neuropathic pain or multisite joint pain. Post-surgery, this group of patients may experience sensitisation subsequent to chronic pain and/or additional neuropathic components, leading to more severe symptoms which places them at greater risk of revision. Further work would be required to investigate whether patients with neuropathic or chronic pain after primary KR would benefit from closer monitoring and follow-up, particularly if they are then at further increased risk of mid-late revision.

In this study, there was an opportunity to examine unique datasets for predictors of mid-late term revision risk for KR surgery. We have reported the results for KR in this study, but it is of interest that in the wider programme of work (UK SAFE[12]), the predictors of revision were different for hips and knees with age being the main consistent finding. The patient factors we identified as predictive of mid-late term revision risk after KR may reflect inequalities in access to revision surgery, or there may be other factors not captured within this study; this requires further investigation. In addition, the effect we observed of pre-operative pain medication (opioids and anticonvulsants) on increased risk of mid-late term revision of KR needs further work to determine if this group of patients require targeted follow-up. The findings from this study have implications for future provision of follow-up services for patients with a KR.

For peer review only

Acknowledgements

We gratefully acknowledge interpretation offered by Mr. Nicholas Howells (North Bristol NHS Trust). We would like to thank the patients and staff of all the hospitals in England and Wales who have contributed data to the National Joint Registry (NJR); and the Healthcare Quality Improvement Partnership (HQIP), the NJR Steering Committee and staff at the NJR Centre for facilitating this work. The authors have conformed to the NJR's standard protocol for data access and publication.

Competing interests statement

LKS reports grants from National Institute for Health Research during the conduct of the study. AJ reports grants from National Institute for Health Research and has received consultancy fees from Freshfields Bruckhaus Derringer and Anthera Pharmaceuticals LTD unrelated to this work. AJ was supported by the NIHR Biomedical Research Centre at University Hospitals Bristol and Weston NHS Foundation Trust and the University of Bristol. NA reports grants from Merck, personal fees from Pfizer/Lilly, unrelated to the submitted work. SRK, MS and PGC were supported in part by the NIHR Leeds Biomedical Research Centre.

Funding statement

This article presents independent research funded by the National Institute for Health Research (NIHR) Health Services and Delivery Research Programme (14/70/146) and by the NIHR Leeds Biomedical Research Centre (BRC). SRK, MS and PGC were supported in part by the NIHR Leeds Biomedical Research Centre. AJ was supported by the NIHR Biomedical Research Centre at University Hospitals Bristol and Weston NHS Foundation Trust and the University of Bristol.

Disclaimer

The views expressed represent those of the authors and do not necessarily reflect those of the NIHR, the NHS, the Department of Health and Social Care, the National Joint Registry Steering Committee or the Healthcare Quality Improvement Partnership (HQIP) who do not vouch for how the information is presented. HES data copyright © 2016, re-used with the permission of the Health & Social Care Information Centre. All rights reserved.

Ethical approval

The CPRD Group has obtained ethical approval from a National Research Ethics Service Committee (NRES) for all purely observational research using anonymised CPRD data; namely, studies which do not include patient involvement. The study has been approved by

For peer review only

ISAC (Independent Scientific Advisory Committee) for MHRA Database Research (protocol number 11_050AMnA2RA2).

For the NJR, before Personal Data and Sensitive Personal Data is recorded, express written patient consent is provided. The NJR records patient consent as either 'Yes', 'No', or 'Not Recorded'. With support under Section 251 of the NHS Act 2006, the Ethics and Confidentiality Committee (ECC), (now the Health Research Authority Confidentiality Advisory Group) allows the NJR to collect patient data where consent is indicated as 'Not Recorded'. Section 251 support (17/CAG/0030) and NHS Digital approval (DARS-NIC-172121-G0Z1H-v0.11) were obtained for the NJR-HES-PROMS dataset analysis.

Data sharing statement

Access to data is available from the National Joint Registry for England and Wales, Northern Ireland and the Isle of Man, but restrictions apply to the availability of these data, which were used under license for the current study, and so are not publicly available. Data access applications can be made to the National Joint Registry Research Committee. Access to linked HES and PROMs data is available through data applications to NHS Digital.

Original protocol

Czoski Murray CJ, *et al*. *BMJ Open* 2019;9:e031351. doi:10.1136/bmjopen-2019-031351(provided as Supplementary File I).

Author's contributions

LKS, SRK, NA, MS, PGC, AJ in conception of work, approval of final version and accountability.

CG, AD, AJ analysis of data

AJ, SRK, MS, LKS, R P-V, PGC in interpretation of results

AJ, LKS drafted and revised final manuscript

For peer review only

References

1. Ethgen O, Bruyere O, Richy F, et al. Health-Related Quality of Life in Total Hip and Total Knee Arthroplasty. A Qualitative and Systematic Review of the Literature. *Journal of Bone and Joint Surgery* 2004;86(5):963-74.
2. Harris WH, Sledge CB. Total Hip and Total Knee Replacement (1). *New England Journal of Medicine* 1990;323(11):725-31. doi: 10.1056/nejm199009133231106
3. Harris WH, Sledge CB. Total Hip and Total Knee Replacement (2). *New England Journal of Medicine* 1990;323(12):801-07. doi: 10.1056/nejm199009203231206
4. Dakin H, Gray A, Fitzpatrick R, et al. Rationing of total knee replacement: a cost-effectiveness analysis on a large trial data set. *BMJ Open* 2012;2(1):e000332. doi: 10.1136/bmjopen-2011-000332 [published Online First: 2012/02/01]
5. Daigle ME, Weinstein AM, Katz JN, et al. The cost-effectiveness of total joint arthroplasty: a systematic review of published literature. *Best Pract Res Clin Rheumatol* 2012;26(5):649-58. doi: 10.1016/j.berh.2012.07.013
6. National Joint Registry. National Joint Registry for England, Wales, Northern Ireland and the Isle of Man. 17th Annual Report., 2020. Available at: https://reports.njrcentre.org.uk/. [Last accessed 23 October 2020].
7. Culliford DJ, Maskell J, Kiran A, et al. The lifetime risk of total hip and knee arthroplasty: results from the UK general practice research database. *Osteoarthritis Cartilage* 2012;20(6):519-24. doi: 10.1016/j.joca.2012.02.636 [published Online First: 2012/03/08]
8. Culliford D, Maskell J, Judge A, et al. Future projections of total hip and knee arthroplasty in the UK: results from the UK Clinical Practice Research Datalink. *Osteoarthritis Cartilage* 2015;23(4):594-600. doi: 10.1016/j.joca.2014.12.022 [published Online First: 2015/01/13]
9. British Orthopaedic Association. 2017 Commissioning guide: Painful osteoarthritis of the knee. Available at: https://www.boa.ac.uk/standards-guidance/commissioning-guides.html. [Last accessed 26 August 2020].
10. National Institute for Health and Care Excellence (NICE). Joint replacement (primary): hip, knee and shoulder. NG157. 04 June 2020. Available at: https://www.nice.org.uk/guidance/ng157/chapter/Recommendations#long-term-care. [Last accessed 26 August 2020].
11. Smith, L.K. Assessment of the current state of hip arthroplasty surveillance in the UK. *Musculoskeletal Care,* 2014; 12 (4) 232-238. https://doi.org/10.1002/msc.1077
12. Czoski Murray, C. et al. Towards UK poSt Arthroplasty Follow-up rEcommendations (UK SAFE). *BMJ Open* 2019;9(6):e031351. doi: 10.1136/bmjopen-2019-031351.
13. Medicines and Healthcare Products Regulatory Agency. Release Notes – CPRD GOLD February 2017 . Available at: https://cprdcw.cprd.com/_docs/Release_Notes_February2017.pdf. [Last accessed 18 October 2020].
14. Padmanabhan S, Carty L, Cameron E, et al. Approach to record linkage of primary care data from Clinical Practice Research Datalink to other health-related patient data: overview and implications. *European Journal of Epidemiology* 2019;34(1):91-99. doi: 10.1007/s10654-018-0442-4
15. Delmestri A, Prieto-Alhambra D. CPRD GOLD and linked ONS mortality records: reconciling guidelines, *International Journal of Medical Informatics* 2020;

For peer review only - http://bmjopen.bmj.com/site/about/guidelines.xhtml

136:104038:1-6. Available at: https://doi.org/10.1016/j.ijmedinf.2019.104038. [Last accessed 23 October 2020].
16. National Joint Registry for England, Wales, Northern Ireland and the Isle of Man. Available at: http://www.njrcentre.org.uk/ [Last accessed 26 August 2020].
17. NHS Digital. Patient Reported Outcome Measures (PROMs). Available at: https://digital.nhs.uk/data-and-information/data-tools-and-services/data-services/patient-reported-outcome-measures-proms [Last accessed 26 August 2020].
18. EuroQol Research Group. EQ-5D. Available at : https://euroqol.org/. [Last accessed 18 October 2020].
19. Dawson J, Fitzpatrick R, Murray D, et al. Questionnaire on the perceptions of patients about total knee replacement. *J Bone Joint Surg Br* 1998;80(1):63-69.
20. Charlson ME, Pompei P, Ales KL, et al. A new method of classifying prognostic comorbidity in longitudinal studies: development and validation. *Journal of chronic diseases* 1987;40(5):373-83. [published Online First: 1987/01/01]
21. Sterne JA, White IR, Carlin JB, et al. Multiple imputation for missing data in epidemiological and clinical research: potential and pitfalls. *Bmj* 2009;338:b2393. doi: 10.1136/bmj.b2393 [published Online First: 2009/07/01]
22. Bayliss LE, Culliford D, Monk AP, et al. The effect of patient age at intervention on risk of implant revision after total replacement of the hip or knee: a population-based cohort study. *Lancet* 2017;389(10077):1424-30. doi: 10.1016/s0140-6736(17)30059-4 [published Online First: 2017/02/18]
23. Culliford D, Maskell J, Judge A, et al. A population-based survival analysis describing the association of body mass index on time to revision for total hip and knee replacements: results from the UK general practice research database. *BMJ Open* 2013;3(11):e003614. doi: 10.1136/bmjopen-2013-003614
24. Dixon T, Shaw M, Ebrahim S, et al. Trends in hip and knee joint replacement: socioeconomic inequalities and projections of need. *Annals of the Rheumatic Diseases* 2004;63(7):825. doi: 10.1136/ard.2003.012724
25. Thillemann TM, Pedersen AB, Mehnert F, et al. The risk of revision after primary total hip arthroplasty among statin users: a nationwide population-based nested case-control study. *J Bone Joint Surg Am* 2010;92(5):1063-72. doi: 10.2106/jbjs.H.01805 [published Online First: 2010/05/05]
26. Prieto-Alhambra D, Javaid MK, Judge A, et al. Association between bisphosphonate use and implant survival after primary total arthroplasty of the knee or hip: population based retrospective cohort study. *BMJ* 2011;343:d7222. doi: 10.1136/bmj.d7222 [published Online First: 2011/12/08]
27. Thillemann TM, Pedersen AB, Mehnert F, Johnsen SP, Soballe K. Postoperative use of bisphosphonates and risk of revision after primary total hip arthroplasty: A nationwide population-based study. *Bone* 2010; 46(4): 946-951. doi.org/10.1016/j.bone.2010.01.377.
28. National Institute for Health and Clinical Excellence. Osteoarthritis: care and management (CG177). 12 February 2014. Available at: https://www.nice.org.uk/guidance/cg177. {Last accessed 20 October 2020].
29. Inacio MCS, Hansen C, Pratt NL, et al. Risk factors for persistent and new chronic opioid use in patients undergoing total hip arthroplasty: a retrospective cohort study. *BMJ Open* 2016;6(4):e010664. doi: 10.1136/bmjopen-2015-010664.

For peer review only - http://bmjopen.bmj.com/site/about/guidelines.xhtml

30. Wylde V, Beswick A, Bruce J, Blom A, Howells N, Gooberman-Hill R. Chronic pain after total knee arthroplasty. *Efort Open Reviews*, 2018. doi.org/10.1302/2058-5241.3.180004.

31. Murray JR, Howells N, Mathews JA, Kalson NS, Porteous AJ. Investigation of the problematic knee replacement. *The Knee*, 2020. https://doi.org/10.1016/j.knee.2020.07.083

For peer review only

For peer review only - http://bmjopen.bmj.com/site/about/guidelines.xhtml

Abbreviations

ASA	American Society of Anaesthesiologists
BMI	Body Mass Index
COPD	Chronic obstructive pulmonary disease
CPRD	Clinical Practice Research Datalink
DDD	Daily defined dose
EQ-5D-3L	EuroQol five domain
GP	General Practitioner
HES	Hospital Episode Statistics
ICD	International Classification of Diseases
ICE	Imputation by Chained Equations
IMD	Index of Multiple Deprivation
KR	Knee replacement
MHRA	Medicines and Healthcare products Regulatory Agency
NHS	National Health Service
NIHR	National Institute for Health Research
NJR	National Joint Registry of England, Wales, Northern Ireland and the Isle of Man
OA	Osteoarthritis
OKS	Oxford Knee Score
ONS	Office for National Statistics
OPCS4	NHS national clinical procedural codes
PPI	Patient and public involvement
PROMs	Patient-reported outcome measure data
UK	United Kingdom

For peer review only - http://bmjopen.bmj.com/site/about/guidelines.xhtml

Supplementary files

Supplementary file I: Additional results

Table A. Descriptive statistics for the CPRD GOLD-HES linked datasets

Table B. Descriptive statistics for the NJR-HES-PROMs linked dataset

Supplementary file II: Original protocol

For peer review only

For peer review only - http://bmjopen.bmj.com/site/about/guidelines.xhtml

Supplementary file

Table A. Descriptive statistics for the CPRD-HES linked datasets

	Knee Replacement
Year of primary	
1995-1999	995 (5.7%)
2000-2004	4486 (25.8%)
2005-2009	8415 (48.4%)
2010-2011	3482 (20.0%)
Age at primary	69.4 (SD 9.2)
Sex	
Female	9963 (57.3%)
Male	7415 (42.7%)
Body mass index	
Underweight	48 (0.4%)
Normal	2204 (16.2%)
Overweight	5239 (38.6%)
Obese Class I (Moderately obese)	3777 (27.8%)
Obese Class II and higher	2306 (17.0%)
Index of Multiple Deprivation (IMD) quintiles	
Least deprived	3890 (22.4%)
2	4145 (23.9%)
3	3918 (22.6%)
4	3078 (17.7%)
Most deprived	2328 (13.4%)
Region	
East Midlands	706 (4.1%)
East of England	2003 (11.5%)
London	1464 (8.4%)
North East	434 (2.5%)
North West	2551 (14.7%)
South Central	2605 (15.0%)
South East Coast	2144 (12.3%)
South West	2386 (13.7%)
West Midlands	2320 (13.4%)
Yorkshire & The Humber	765 (4.4%)
Smoker	
Ex-smoker	5122 (34.6%)
Non-smoker	8310 (56.2%)
Current	1368 (9.2%)

For peer review only

Alcohol	
Ex-smoker	322 (2.7%)
No	2242 (18.8%)
Yes	9371 (78.5%)
Recorded diagnosis of hip OA	6841 (39.4%)
Hip Fracture prior primary surgery	119 (0.7%)
Fracture in pelvis, proximal/humerus, wrist/forearm, spine or rib	537 (3.1%)
Comorbidities	
asthma	1713 (9.9%)
malabsorption	42 (0.2%)
inflammatory bowel disease	128 (0.7%)
hypertension	6106 (35.1%)
hyperlipidaemia	2223 (12.8%)
ischaemic heart disease	1685 (9.7%)
myocardial infarction	345 (2.0%)
stroke/cerebrovascular disease	585 (3.4%)
chronic pulmonary disease	498 (2.9%)
chronic kidney failure	1277 (7.4%)
cancer	1446 (8.3%)
diabetes	1774 (10.2%)
Drugs which can affect fracture risk prior primary surge	
Calcium and vitamin D supplements	1377 (7.9%)
Bisphosphonates	1161 (6.7%)
Selective oestrogen receptor modulators	33 (0.2%)
Oral glucocorticosteroid therapy	3521 (20.3%)
Drugs prior primary surgery	
Proton pump inhibitors	7586 (43.7%)
Anti-arrhythmics	1700 (9.8%)
Anticonvulsants	865 (5.0%)
Antidepressants	5875 (33.8%)
Anti-Parkinson drugs	305 (1.8%)
Statins	5697 (32.8%)
Thiazide diuretics	8498 (48.9%)
Anxiolitics	3406 (19.6%)
Painkillers/anti-inflammatory drugs	
NSAIDs	15406 (88.7%)
NSAID cox	3155 (18.2%)
Paracetamol	14438 (83.1%)
Partial Opiates	13334 (76.7%)
Total Opiates	6459 (37.2%)
Steroid iFuse Implant System® Minimally Invasive Arthrodesis (iMIA)	5401 (31.1%)

For peer review only

DDDs (daily defined dose) 1-year prior surgery	
Calcium and vitamin D supplements	
No dose	16001 (92.1%)
<120 DDD	281 (1.6%)
>=120 to 340 DDD	503 (2.9%)
>340 DDD	222 (1.3%)
Dose missing	371 (2.1%)
Bisphosphonates	
No dose	16217 (93.3%)
<140 DDD	229 (1.3%)
>=140 to 340 DDD	374 (2.2%)
>340 DDD	260 (1.5%)
Dose missing	298 (1.7%)
Selective oestrogen receptor modulators	
No dose	17345 (99.8%)
<280 DDD	8 (0.1%)
>=280 to 390 DDD	8 (0.1%)
>390 DDD	0 (0%)
Dose missing	17 (0.1%)
Oral glucocorticosteroid therapy	
No dose	13857 (79.7%)
<30 DDD	493 (2.8%)
>=30 to 280 DDD	458 (2.6%)
>280 DDD	316 (1.8%)
Dose missing	2254 (13.0%)
Proton pump inhibitors (no dose)	
No dose	9792 (56.4%)
<85 DDD	1376 (7.9%)
>=85 to 365 DDD	2847 (16.4%)
>365 DDD	995 (5.7%)
Dose missing	2368 (13.6%)
Anti-arrhythmics (no dose)	
No dose	15678 (90.2%)
<170 DDD	159 (0.9%)
>=170 to 365 DDD	241 (1.4%)
>365 DDD	158 (0.9%)
Dose missing	1142 (6.6%)
Anticonvulsants	
No dose	16513 (95.0%)
<85 DDD	132 (0.8%)
>=85 to 365 DDD	212 (1.2%)
>365 DDD	111 (0.6%)
Dose missing	410 (2.4%)

For peer review only

Antidepressants	
No dose	11503 (66.2%)
<85 DDD	786 (4.5%)
>=85 to 365 DDD	1418 (8.2%)
>365 DDD	565 (3.3%)
Dose missing	3106 (17.9%)
Anti-Parkinson drugs	
No dose	17073 (98.2%)
<200 DDD	36 (0.2%)
>=200 to 600 DDD	90 (0.5%)
>600 DDD	41 (0.2%)
Dose missing	138 (0.8%)
Statins	
No dose	11681 (67.2%)
<280 DDD	1248 (7.2%)
>=280 to 370 DDD	2522 (14.5%)
>370 DDD	1383 (8.0%)
Dose missing	544 (3.1%)
Thiazide diuretics	
No dose	8880 (51.1%)
<225 DDD	1678 (9.7%)
>=225 to 390 DDD	2826 (16.3%)
>390 DDD	1565 (9.0%)
Dose missing	2429 (14.0%)
Anxiolitics	
No dose	13972 (80.4%)
<30 DDD	367 (2.1%)
>=30 to 350 DDD	531 (3.1%)
>350 DDD	344 (2.0%)
Dose missing	2164 (12.5%)
NSAIDs	
No dose	1972 (11.4%)
<60 DDD	2428 (14.0%)
>=60 to 300 DDD	4602 (26.5%)
>300 DDD	2352 (13.5%)
Dose missing	6024 (34.7%)
NSAID cox	
No dose	14223 (81.8%)
<60 DDD	355 (2.0%)
>=60 to 280 DDD	553 (3.2%)
>280 DDD	267 (1.5%)
Dose missing	1980 (11.4%)
Paracetamol	

For peer review only

No dose	2940 (16.9%)
<40 DDD	2796 (16.1%)
>=40 to 200 DDD	5521 (31.8%)
>200 DDD	2425 (14.0%)
Dose missing	3696 (21.3%)
Opioids mix	
No dose	4044 (23.3%)
<30 DDD	2074 (11.9%)
>=30 to 180 DDD	4002 (23.0%)
>180 DDD	1976 (11.4%)
Dose missing	5282 (30.4%)
Opioids total	
No dose	10919 (62.8%)
<200 DDD	995 (5.7%)
>=200 to 600 DDD	1916 (11.0%)
>600 DDD	871 (5.0%)
Dose missing	2677 (15.4%)
Steroid IMIA	
No dose	11977 (68.9%)
<55 DDD	1292 (7.4%)
>=55 DDD	597 (3.4%)
Dose missing	3512 (20.2%)

For peer review only

Table B. Descriptive statistics for the NJR-HES-PROMs linked dataset

		Knee Replacement
Year of primary		
	2008	33504 (17.8%)
	2009	45928 (42.1%)
	2010	52460 (70.0%)
	2011	56617 (100.0%)
Age at primary knee replacement		69.9 (SD 9.3)
Sex		
Female		106812 (56.7%)
Male		81697 (43.3%)
Body mass index		30.9 (SD 5.5)
Index of Multiple Deprivation (IMD) quintiles		
Least deprived		40247 (21.6%)
2		42721 (22.9%)
3		35839 (19.2%)
4		34691 (18.6%)
Most deprived		33119 (17.8%)
Rurality, at primary		
urban >=10,000		140202 (74.5%)
town and fringe		22366 (11.9%)
village/isolated		25618 (13.6%)
ethnicity		
white		161079 (92.9%)
non-white		12237 (7.1%)
Number comorbidities at primary (none)		
None		141570 (75.1%)
Mild		38083 (20.2%)
Moderate		6875 (3.7%)
Severe		1981 (1.1%)
ASA grade		
P1 - Fit and healthy		20087 (10.7%)
P2 - Mild disease not incapacitating		138997 (73.7%)
P3 - P5		29425 (15.6%)
Minimally invasive (no)		
No		176683 (93.7%)
Yes		11826 (6.3%)
Surgical volume per consultant		
<=10 operations		5312 (2.8%)
11-50		58102 (30.8%)
51-75		42101 (22.3%)
76-100		33558 (17.8%)

For peer review only

101-150	32381 (17.2%)
>150	17055 (9.1%)
Surgeon experience	
<8 training years	44650 (23.7%)
Consultant (≥8 training years)	143859 (76.3%)
Surgical approach (knee)	
Lateral parapatellar	1756 (0.9%)
Medial parapatellar	175112 (92.9%)
Mid-Vastus	5710 (3.0%)
Sub-Vastus	2300 (1.2%)
Other	3631 (1.9%)
Primary graft femur	
No	186951 (99.2%)
Yes	1558 (0.8%)
Implant fixation	
Cementless	9429 (5.0%)
cemented	177031 (94.0%)
Hybrid	1947 (1.0%)
Primary graft tibia	
No	187679 (99.6%)
Yes	830 (0.4%)
Type of primary implant	
UKR	13266 (7.0%)
TKR	175243 (93.0%)
Type of mechanical thromboprophylaxis	
None	16746 (8.9%)
Any	171763 (91.1%)
Type of chemical thromboprophylaxis	
None	13239 (7.0%)
Aspirin only	16356 (8.7%)
LMWH (+/-Other)	134648 (71.4%)
Other (no LMWH)	24266 (12.9%)
Unit type	
Public hospital	153780 (81.6%)
Independent sector - hospital	24716 (13.1%)
Independent sector - treatment centre	10013 (5.3%)
OKS, baseline score	18.0 (SD 7.8)
EQ-5D Anxiety Depression	
I am not anxious or depressed	65123 (62.4%)
I am moderately anxious or depressed	32676 (31.3%)
I am extremely anxious or depressed	3710 (3.6%)

For peer review only

Open access Protocol

BMJ Open

Towards UK poSt Arthroplasty Follow-up rEcommendations (UK SAFE): protocol for an evaluation of the requirements for arthroplasty follow-up, and the production of consensus-based recommendations

Carolyn J Czoski Murray,[1] Sarah R Kingsbury,[2,3] Nigel K Arden,[4] Jenny Hewison,[1] Andrew Judge,[5,6] Jamie Matu,[1,2] Jamie O'Shea,[7] Rafael Pinedo-Villanueva,[4] Lindsay K Smith,[8] Chris Smith,[1] Christine M Thomas,[2,3] Robert M West,[1] Judy M Wright,[1] Philip G Conaghan,[2,3] Martin H Stone[3,9]

To cite: Czoski Murray CJ, Kingsbury SR, Arden NK, *et al*. Towards UK poSt Arthroplasty Follow-up rEcommendations (UK SAFE): protocol for an evaluation of the requirements for arthroplasty follow-up, and the production of consensus-based recommendations. *BMJ Open* 2019;**9**:e031351. doi:10.1136/bmjopen-2019-031351

► Prepublication history and additional material for this paper are available online. To view these files, please visit the journal online (http://dx.doi.org/10.1136/bmjopen-2019-031351).

PGC and MHS contributed equally.
CJCM and SRK contributed equally.

Received 29 April 2019
Revised 21 May 2019
Accepted 22 May 2019

Check for updates

© Author(s) (or their employer(s)) 2019. Re-use permitted under CC BY. Published by BMJ.

For numbered affiliations see end of article.

Correspondence to
Professor Philip G Conaghan;
p.conaghan@leeds.ac.uk

ABSTRACT

Introduction Hip and knee arthroplasties have revolutionised the management of degenerative joint diseases and, due to an ageing population, are becoming increasingly common. Follow-up of joint prostheses is to identify problems in symptomatic or asymptomatic patients due to infection, osteolysis, bone loss or potential periprosthetic fracture, enabling timely intervention to prevent catastrophic failure at a later date. Early revision is usually more straight-forward surgically and less traumatic for the patient. However, routine long-term follow-up is costly and requires considerable clinical time. Therefore, some centres in the UK have curtailed this aspect of primary hip and knee arthroplasty services, doing so without an evidence base that such disinvestment is clinically or cost-effective.

Methods Given the timeline from joint replacement to revision, conducting a randomised controlled trial (RCT) to determine potential consequences of disinvestment in hip and knee arthroplasty follow-up is not feasible. Furthermore, the low revision rates of modern prostheses, less than 10% at 10 years, would necessitate thousands of patients to adequately power such a study. The huge variation in follow-up practice across the UK also limits the generalisability of an RCT. This study will therefore use a mixed-methods approach to examine the requirements for arthroplasty follow-up and produce evidence-based and consensus-based recommendations as to how, when and on whom follow-up should be conducted. Four interconnected work packages will be completed: (1) a systematic literature review; (2a) analysis of routinely collected National Health Service data from five national data sets to understand when and which patients present for revision surgery; (2b) prospective data regarding how patients currently present for revision surgery; (3) economic modelling to simulate long-term costs and quality-adjusted life years associated with different follow-up care models and (4) a Delphi-consensus process, involving all stakeholders, to develop a policy document which includes a stratification algorithm to determine appropriate follow-up care for an individual patient.

Ethics and Dissemination Favourable ethical opinion has been obtained for WP2a (RO-HES) (220520) and WP2B (220316) from the National Research Ethics Committee. Following advice from the Confidentiality Advisory Group (17/CAG/0122), data controllers for the data sets used in WP2a (RO-HES) – NHS Digital and The Phoenix Partnership – confirmed that Section 251 support was not required as no identifiable data was flowing into or out of these parties. Application for approval of WP2a (RO-HES) from the Independent Group Advising on the Release of Data (IGARD) at NHS Digital is in progress (DARS-NIC-147997). Section 251 support (17/CAG/0030) and NHS Digital approval (DARS-NIC-172121-G0Z1H-v0.11) have been

Strengths and limitations of this study

- ► Our mixed-methods approach allows us to address a question that would not be feasible to answer with a randomised controlled trial.
- ► Our study will capture data from a mixture of teaching hospitals, district general hospitals and hospitals with a special interest in joint replacement and with a geographical spread, increasing the generalisability of our results.
- ► Our economic model will be populated with routinely collected National Health Service (NHS) data of patients attending primary and hospital care in the UK, ensuring that our analysis is based on actual patient use of services, outcomes such as health-related quality of life and costs to the NHS.
- ► While our analysis is based on data sources that reflect clinical practice in England only, we believe key cost-effectiveness findings are likely to be informative for decision-making in the whole of the UK.

BMJ Open: first published as 10.1136/bmjopen-2019-031351 on 25 June 2019. Downloaded from http://bmjopen.bmj.com/ on 15 July 2019 by guest. Protected by copyright.

For peer review only - http://bmjopen.bmj.com/site/about/guidelines.xhtml

obtained for WP2a (NJR-HES-PROMS). ISAC (11_050MnA2R2) approval has been obtained for WP2a (CPRD-HES).

INTRODUCTION

Arguably, total hip arthroplasty (THA) and total knee arthroplasty (TKA) are the most successful surgical interventions performed in modern times. Due to an ageing population, and an obesity epidemic, hip and knee replacement procedures increase annually, rising from less than 20 000/year in the UK in 1978 to around 200 000/year in 2017.[1] The current follow-up requirements are estimated at 500 000–1 000 000 annual outpatient attendances. With limitless resources, every patient undergoing a joint arthroplasty would incur routine lifetime follow-up. The rationale for follow-up is to ensure timely detection of complications or arthroplasty failure, such as aseptic loosening, osteolysis and potential periprosthetic fracture. The cost of revision for aseptic loosening is 35% lower than that for periprosthetic fractures and has a lower incidence of complications which impact recovery.[2] However, while routine long-term follow-up of joint prostheses may support timely revision for patients with asymptomatic complications, improving long-term health outcomes, it is also costly both clinically and financially.

Orthopaedic services are already one of the poorest performers across the National Health Service (NHS) by failing to meet waiting list targets, with an estimated 8000 orthopaedic NHS breaches each month.[3] With a rapidly ageing population and medical advances that mean less stringent criteria for surgery eligibility,[4] there is no sign that demand will recede in coming years and orthopaedic services will soon reach breaking point. To reduce the burden on orthopaedic services, evidence-based consensus guidelines are required to establish how, when and on whom follow-up should be conducted.

British Hip Society (BHS) and British Orthopaedic Association (BOA) guidelines recommend outpatient follow-up at 1 and 7 years, and every 3 years thereafter for Orthopaedic Data Evaluation Panel 10A (ODEP-10A) implants, with more frequent follow-up for novel implants.[5] However, recent work revealed considerable diversity across the UK in arthroplasty follow-up pathways, in timing, how follow-up is conducted and which health professionals are involved.[6] While some centres followed-up patients beyond 10 years, others did not have an established follow-up policy and in some centres follow-up services have been curtailed or stopped entirely after an early postoperative check.[6] Notably, we do not know whether long-term follow-up is cost-effective or whether disinvestment is safe for patients.

This project aims to determine the consequences of disinvestment in hip and knee arthroplasty follow-up. Given the timeline from joint replacement to revision, with a 7% revision rate for THA and 4% revision rate for TKA at 14 years, conducting a randomised controlled trial to address this question is not feasible. Moreover, the huge variation in follow-up practice across the UK limits the generalisability of the results of an RCT. We will therefore use a mixed-methods approach to comprehensively evaluate the requirements for arthroplasty follow-up and will use this evidence to inform the development of consensus-based recommendations and a policy document which includes a stratification algorithm to determine appropriate follow-up for individual patients. Disinvestment is a complex and often contentious issue. We plan to make use of published recommendations[7] to ensure that the results of this work are understood and considered as a genuine attempt to use the best evidence available to ensure that the NHS gets value for money and that patients remain safe.

METHODS AND ANALYSIS

Study objectives

A. Identify who needs follow-up and when this should occur for primary THA, TKA and unicompartmental knee arthroplasty (UKA) surgery by making use of routinely collected NHS data.
B. Understand the patient journey (in primary and secondary care) to revision surgery by recruiting patients admitted for elective and emergency hip and knee revision surgery.
C. Establish how and when patients are identified for revision, why some patients are missed from regular follow-up and present acutely with fracture around the implant (periprosthetic fracture), by using prospective and retrospective data.
D. Identify the most appropriate and cost-effective follow-up pathway to minimise potential harm to patients by undertaking cost-effectiveness modelling.
E. Provide evidence-based and consensus-based recommendations on how follow-up of primary THA and TKA should be conducted.

Design

This is a mixed-methods study using a variety of data sources consisting of four interconnected work packages (WP): (1) a systematic literature review; (2a) analysis of routinely-collected NHS data to understand when and which patient present for revision surgery; (2b) prospective data regarding how patients currently present for revision surgery collected on around 455 patients prior to elective or emergency revision surgery; (3) economic modelling to simulate long-term costs and quality adjusted life years associated with different follow-up models; (4) a Delphi-consensus process, incorporating all previous WPs and involving all stakeholders, to develop a policy document which includes a stratification algorithm to determine appropriate follow-up for an individual patient.

WP1: systematic review

The aim of the review is to evaluate different models of routine long-term follow-up care after TKA/THA/UKA. This systematic review will establish a robust evidence base

BMJ Open: first published as 10.1136/bmjopen-2019-031351 on 25 June 2019. Downloaded from http://bmjopen.bmj.com/ on 15 July 2019 by guest. Protected by copyright.
For peer review only

For peer review only - http://bmjopen.bmj.com/site/about/guidelines.xhtml

for the cost-effectiveness modelling (WP3) and consensus guideline development (WP4).

Registration

This systematic review will be undertaken following Cochrane Collaboration methods[8] and reported in accordance with Preferred Reporting Items for Systematic Review and Meta-analyses guidelines.[9] It has been prospectively registered with PROSPERO (CRD42017053017).

Searches

A comprehensive literature search will be undertaken with the aim of retrieving all relevant literature, published or unpublished, which evaluated the effectiveness of long-term follow-up after primary TKA/THA/UKA. A range of information sources will be searched: BIOSIS, CINAHL, ClinicalTrials.gov, The Cochrane Library, Embase, Health Management Information Consortium, IDEAS (RePEC), Ovid Medline(R), ProQuest Dissertations and Theses, PsycINFO, PubMed and Web of Science. Reference lists of included studies will be reviewed for potentially relevant articles. A sample search strategy is detailed in the online supplementary appendix A. No date or language restrictions will be applied.

Criteria for selection of studies

All study designs will be included which (1) consider the clinical and/or cost effectiveness of routine long-term (>5 years) follow-up care after primary THA, TKA or UKA; (2) describe patient safety issues associated with routine follow-up or (3) consider the acceptability of new care pathways from the perspective of the patient and/or practitioner. Studies will be excluded if they do not report specific patient-related outcome measures or appropriate health utility measures.

Selection of studies

Titles/abstracts of identified studies will be screened for eligibility by one experienced reviewer with a random selection (25%) independently screened by a second. Potential studies will be retrieved in full text and reviewed against the inclusion/exclusion criteria independently by the same two reviewers, with a third reviewer used to settle any disputes.

Data extraction

Data will be extracted by a single reviewer using a standardised proforma capturing (1) purpose and design; (2) methodological characteristics; (3) information relating to quality assessment and (4) outcome data relating to the clinical and cost-effectiveness of routine long-term follow-up care.

Quality assessment

The Cochrane Risk of Bias assessment tool will be used for experimental studies,[10] and the Newcastle-Ottawa scales for cohort and case–control studies.[11] Qualitative literature will be assessed using critical interpretive synthesis.[12] Economic evaluations will be assessed using the Drummond checklist.[13] Studies will be evaluated independently by two reviewers, with a third to settle any disputes. Studies at high risk of bias will not be excluded and conclusions will incorporate observed biases.

Evidence synthesis

The design, methodological characteristics, study quality and main findings will be summarised in narrative and tabular form. We anticipate substantial heterogeneity among included studies precluding the use of meta-analysis techniques.

WP2a: Analysis of routinely collected NHS data

This WP will use routinely collected NHS data to determine when revision happens and to identify patients most likely to require revision in order to target when and on whom follow-up should occur.

Data sources

Data from five national datasets will be used: (1) Clinical Practice Research Database (CPRD),[14] (2) ResearchOne (RO),[15] (3) Hospital Episode Statistics (HES),[16] (4) National Joint Registry (NJR)[17] and (5) patient reported outcome measures (PROMs).[18]

Three linked data sets will be constructed for analysis: (a) CPRD–HES–PROMS, which preexists at the University of Oxford, (b) RO–HES will be constructed and analysed at the University of Leeds. Linkage will be undertaken by NHS Digital on the basis of pseudonyms generated from NHS numbers by the data providers. (c) NJR–HES–PROMS will be constructed and analysed at the University of Oxford. Linkages will be undertaken by NHS Digital, using an agreed set of common patient identifiers, including NHS number. Data sets (a) and (b) provide a primary care view (eg, prior diagnoses, prescribing) and include different, representative patient populations for cross-validation; data set (c) provides a secondary care view (eg, surgeon, procedure details).

Data analysis

The primary outcome of the analysis will be mid-late term revision (>5 years post-primary surgery), defined as the removal, exchange or addition of any of the components of arthroplasty. Exposures will include secondary care predictors, including patient level characteristics recorded in NJR and HES (eg, age, body mass index (BMI)), surgical and operative factors and symptoms of pain, function and health-related quality of life preoperatively and 6 months post-surgery from PROMS, and primary care predictors, including patient demographics, comorbidities and use of drugs which can affect fracture risk. Survival analysis will be used to model time to revision.[19 20] The smoothed Nelson-Aalen cumulative hazard rate will be examined to identify any peak in the mid-long term risk of revision. Cox proportional hazards regression modelling will be used to identify preoperative, perioperative and postoperative predictors of mid-late term revision, for example, age, BMI, comorbidities, implant type, surgeon skill and postoperative problems. Competing risk

For peer review only

BMJ Open: first published as 10.1136/bmjopen-2019-031351 on 25 June 2019. Downloaded from http://bmjopen.bmj.com/ on 15 July 2019 by guest. Protected by copyright.

regression will be used, since mortality can be regarded as a competing risk for revision surgery.[21 22] To account for clustering within the data (such as patients nested within hospitals), a multilevel survival model will be fitted by extending the survival regression model to include a frailty term with a Gaussian distribution.[23]

WP2b part 1: multicentre observational prospective cohort

Prospective data collection from patients undergoing revision surgery.

Objectives:

- Identify all recent (previous 12 months) medical appointments and advice sessions related to the index joint in primary and secondary care.
- Establish if the patient has been seen by orthopaedic health professionals from 12 months after primary surgery until this hospital admission, that is, was the revision directed by routine follow-up.

Design

A multicentre, observational, single visit, prospective cohort study of patients admitted for revision hip or knee surgery.

Population

Patients presenting for elective and emergency revision surgery of a primary THA, TKA or UKA and who are able and willing to provide written informed consent will be included in the study. Patients will be excluded if they have had previous revision surgery; metal-on-metal primary joint replacement or hip hemiarthroplasty. Participants will be recruited from a sample of hospitals selected to provide geographical spread and representation of teaching hospitals, district general hospitals and hospitals with a special interest in joint replacement

Data collection

A participant case report form (CRF) will capture details of follow-up after primary surgery and pathway to current revision surgery, including symptom state. An investigator CRF will extract data from medical notes including demographics (age, gender, diagnosis leading to primary surgery, medical history), general practitioner and hospital appointments, details of primary and revision surgery (including implant type, complications, length of stay). The participant CRF will be piloted with the Leeds Biomedical Research Centre Patient and Public Involvement (PPI) group and the investigator CRF with two research nurses to ascertain the comprehension, usability and completeness of data subsequently extracted.

Sample size

We will use stratified sampling to recruit centres of varying size and anticipate that the average number of patients per centre will be 45 (based on NJR records and information from prospective centres). We initially anticipated the recruitment of 25 centres. With a recruitment rate of 60%, this gave 27 recruited patients from 25 centres (n=675). We do not know the intraclass correlation coefficient (ICC) for our primary outcome ('Was the revision a result of routine follow-up?'), but we anticipate it to be in the region of 0.01–0.05. To be conservative, we use ICC=0.05. This gives a design factor of 2.3 and hence an effective sample size of 293 after accounting for clustering within centre. The enrolment of 35 centres reduced the design factor to 1.6 and the total sample size required to 455. From previous research,[6] we estimate that the rate of our primary outcome is 20% so that the effective number of events will be 58. Hence, we will have sufficient power for our logistic regression to robustly estimate the coefficients of up to five potential risk factors derived from our brief patient survey.[24]

Analysis

The primary outcome will be 'revision identified through routine follow-up', and this will be modelled through a multilevel logistic regression model, with a centre-level random intercept of particular interest. The size of the centre-level effect will be assessed as the proportion of variance explained and will also be assessed through a likelihood ratio test. Up to five factors from the patient questionnaire will be explored as fixed effects at the patient level. This will adjust for case mix. Factors that are found to be both clinically and statistically significant could potentially contribute to a stratified approach to follow-up.

WP2b part 2: qualitative study

Building on previous work highlighting the changes in follow-up practice,[6] this WP aims to explore the rationale and motivating factors behind these changes, the facilitators and the evidence considered when implementing new pathways, including no follow-up.

Sampling

A sample of n=20–30 orthopaedic practitioners and/or unit managers will be recruited. Purposive sampling via sampling matrix will recruit participants with different experiences of a range of follow-up pathways while reflecting NHS trust type, geographical area (urban, rural); socioeconomic area (low/high socioeconomic status) and diverse ethnicity. Some selection criteria are likely to be nested (eg, hospital type, geographical area) and care will be taken to ensure that all viewpoints are represented.

Data collection

Semistructured, telephone interviews following a topic guide refined from the literature review and expert opinion (clinician coapplicants/advisors and PPI members). The researcher will probe pertinent initial responses and expand on issues raised. Interviews will be recorded and transcribed verbatim.

Data analysis

The guiding approach will be framework analysis.[25] Data analysis will comprise five stages: (1) data familiarisation;

BMJ Open: first published as 10.1136/bmjopen-2019-031351 on 25 June 2019. Downloaded from http://bmjopen.bmj.com/ on 15 July 2019 by guest. Protected by copyright.

For peer review only - http://bmjopen.bmj.com/site/about/guidelines.xhtml

(2) identifying the thematic framework; (3) indexing; (4) charting and (5) mapping and interpreting. The process of familiarisation enables the researcher to identify emerging themes or issues in the data. Little is known about why NHS trusts have chosen to either withdraw follow-up care or change the way it is delivered. The evidence generated from the literature review and input from our clinical coapplicants will be used to help identify and refine the thematic framework. Themes are flexible and can be modified in the light of new data, and a process of constant comparison will be undertaken across themes and cases.

WP3

As previous work conducted by members of our team has identified considerable heterogeneity in current follow-up pathways,[6] our cost-effectiveness analysis will compare the relative costs and quality-adjusted life years associated with having follow-up compared with not having follow-up. A third hypothetical scenario of a virtual follow-up will be considered.

Comparators

Both the findings from our systematic review and the prospective cohort will inform the criteria to be used to identify patients as having or not having follow-up. The 7-year reference point for a follow-up currently suggested by BHS and BOA guidelines is likely to be incorporated. Patients having an orthopaedic outpatient appointment around the reference point(s) following a primary arthroplasty will be used to group patients in the CPRD–HES–PROMS data set into the follow-up and no follow-up groups. Joint-specific revision procedures will be identified by OPCS-4 codes as reported in the Admitted Patient Care data set within HES, with corresponding linked records to primary care and PROMS.

Model structure

To identify the most appropriate modelling approach for the question and data at hand, we will conduct a series of preliminary analysis to determine if a cohort-level or patient-level decision analytic model should be employed. Previous models examining the long-term cost-effectiveness of hip and knee replacements have used cohort Markov models.[26 27] Analyses will include associations between patients' characteristics and revision rates, health utilities and costs and whether the risk for revision depends on the time patients stay unrevised after their primary. Regardless of the chosen model type, the key health state or event will be revision arthroplasty, with death and complications also considered. The model will be designed to cover patients' lifetime and analysed from an NHS and Personal Social Services perspective, with discounting of costs and outcomes as per current guide to the methods of technology appraisal.[28]

Model inputs

WP2 data sets will be used to quantify primary and hospital healthcare resource use for comparator groups of follow-up care models through estimation of NHS costs and health-related quality of life (HRQoL). The economic model will simulate long-term costs and quality adjusted life years (QALYs) associated with each care model. Primary care costs will include consultations, and hospital costs will be derived by grouping hospital episodes into Health Resource Groups, a set of casemix groupings utilising similar levels of healthcare resources. Panel data regression analysis[29–31] will be used to estimate hospital costs conditional on patient characteristics and comorbidities. QALYs and transition probabilities will be derived from the linked data sets and published literature as needed. The hypothetical costs of virtual follow-up will be based on similar virtual clinic alternatives previously studied and NHS X-ray-associated costs.

Analysis

Cost-effectiveness analyses will be performed separately for relevant patient subgroups based on gender, age and other potential covariates for which data may be available. As with all economic models, a number of assumptions will be made, and their plausibility and potential impact discussed, relating to model structure and input parameters for transition probabilities, health utilities and costs, including the cost of periprosthetic fractures if no reference is found for these in the literature. Sensitivity analyses will be conducted to explore the uncertainty associated with key assumptions and model parameters and the implications of using different estimates discussed.

WP4: Delphi-consensus process

This WP will use the collective evidence from WP1–3 to inform a consensus process to determine appropriate follow-up care pathways for hip and knee arthroplasty.

Evidence gathered from WP1–3 will feed into a consensus panel workshop. We intend to use methods employed by the National Institute for Health and Care Excellence (NICE) in both the technology assessment committees and Guideline Development Groups. The expert stakeholders invited to attend will have a special interest in patient follow-up after hip or knee replacement surgery. Participants will include patients, orthopaedic surgeons, arthroplasty practitioners, NHS managers and commissioners, manufacturers and representatives of the major orthopaedic bodies (including BOA, BHS and BASK). The purpose of this exercise is to consider the evidence and obtain agreement for future care pathways, supported by the evidence of their effectiveness and cost-effectiveness, to be recommended and adopted across the NHS. Following the NICE consensus model all participants will receive summaries of the main research findings in advance. There will be presentations from the work-stream leaders to outline the evidence for consideration.

Robert *et al*[7] demonstrate that decommissioning is often about more than the 'evidence' and that withdrawal of previously available services is often seen as being driven by the wrong kind of evidence, based on cost data and

BMJ Open: first published as 10.1136/bmjopen-2019-031351 on 25 June 2019. Downloaded from http://bmjopen.bmj.com/ on 15 July 2019 by guest. Protected by copyright.

For peer review only

For peer review only - http://bmjopen.bmj.com/site/about/guidelines.xhtml

political priorities and not on what patients and service users value.[7] It is a complex issue, perhaps as contentious as NICE decisions when they do not fund an effective intervention because it exceeds the threshold. However, NICE investment decisions are made with the explicit understanding that, with no increase in the budget, there must be some displacement of other healthcare technologies.[32] We plan to make use of the recommendations for engagement and the use of evidence outlined in Robert *et al* to ensure the results of this work are understood and considered as a genuine attempt to use the best evidence available to ensure that the NHS gets value for money and that patients remain safe.

Patient and public involvement

Members of the NIHR Leeds BRC, Oxford and Bristol PPI groups are involved in UK SAFE. The PPI co-applicant is a member of the study steering committee and contributes across all WPs. Two independent PPI advisors sit on the Independent Advisory Group. Specific areas where lay involvement will be pivotal include the interpretation of results of the systematic review, the expert panel discussion and consensus process, study oversight (steering group), preparation of patient material and study results and contribution to reports and newsletters for patients and NHS staff.

ETHICS AND DISSEMINATION

All studies will be conducted in accordance with the principles of Good Clinical Practice, and the UK Policy Framework for Health and Social Care Research, 2018. Favourable ethical opinion has been obtained for WP2a (RO-HES) (220520) and WP2B (220316) from the National Research Ethics Committee. Following advice from the Confidentiality Advisory Group (17/CAG/0122), data controllers for the data sets used in WP2a (RO–HES)—NHS Digital and The Phoenix Partnership—confirmed that Section 251 support was not required as no identifiable data was flowing into or out of these parties. Application for approval of WP2a (RO–HES) from the Independent Group Advising on the Release of Data (IGARD) at NHS Digital is in progress (DARS-NIC-147997). Section 251 support (17/CAG/0030) and NHS Digital approval (DARS-NIC-172121-G0Z1H-v0.11) have been obtained for WP2a (NJR-HES-PROMS). ISAC (11_050MnA2R2) approval has been obtained for WP2a (CPRD–HES).

At the end of the project, outputs will be disseminated nationally in the form of an executive summary statement of the agreed pathway/s through appropriate NHS Networks, NICE, the NHS England Elective Orthopaedics Sub-committee, the NHS Institute for Innovation and Improvement and professional societies, including BHS, BOA, BASK, Arthroplasty Care Practitioners Association and the NJR. Dissemination will be key to developing a culture of 'finding the best way of doing something and doing it everywhere' to significantly reduce wastage of clinical resources and optimise NHS spend. We will put forward the consensus statement to each society's AGM for adoption as a resolution. Internationally, dissemination platforms are in place through the International Society of Arthroplasty Registers (ISAR) and the European Federation of National Associations of Orthopaedics and Traumatology. A lay summary of the project will be produced for study participants. Findings will also be presented at relevant orthopaedic and methodological conferences, such as the BOA and the Exploiting Existing Data for Health Research conference. The chief investigator and co-applicants will be named as authors on main publications, and an appropriate first author agreed through discussion. Other key individuals will be included as authors or contributors as appropriate, at the discretion of the Senior Management Group. Any disputes relating to authorship will be resolved by the Steering Committee.

The Chair and Independent members of the Steering Committee will be acknowledged, but will not qualify for full authorship, in order to maintain their independence. Individual collaborators must not publish data concerning their participants' which are directly relevant to the questions posed in the study until the main results of the study have been published.

CONCLUSION

This research will deliver the first research-supported, best-for-patient, joint-specific, cost-effective recommendations for follow-up pathways, providing a gold standard for clinical excellence and follow-up advice for patients, surgeons, purchasers and the NHS as a whole. Value is not limited to the UK, but has substantial global impact potential.

The impact of this work will be to reduce the burden on patients and the NHS in terms of outpatient visits and clinical tests that do not add benefit, while optimising detection of potential problems. From an NHS perspective, this work will provide managers with economic and clinical information on arthroplasty follow-up to inform service planning and delivery, and the role of arthroplasty practitioners in this service, with the potential to reduce geographical disparity through NHS trusts modelling their service provision on a national evidence-based guideline; provide orthopaedic surgeons with guidance on follow-up, including patient and economic considerations of factors involved; produce arthroplasty follow-up guidelines for adoption by the relevant specialist societies and information for their members. From a patient perspective, this work will help to inform patients about follow-up practice, empower them to make choices about future healthcare relating to their joint arthroplasty and provide reassurance that their follow-up pathway is appropriate

The outputs of this project, in terms of evidence-based support for timing of follow-up and identification of the most cost-effective follow-up model, fit directly within the

BMJ Open: first published as 10.1136/bmjopen-2019-031351 on 25 June 2019. Downloaded from http://bmjopen.bmj.com/ on 15 July 2019 by guest. Protected by copyright.

For peer review only - http://bmjopen.bmj.com/site/about/guidelines.xhtml

NHS framework for improving outcomes from elective procedures. Rationalising current diversity of follow-up practices should enable substantial savings for the NHS. We envisage outputs to be readily applicable to the wider NHS, not only hip and knee but also other joint replacements. With the committed support of key national and international organisations already in place, we anticipate that these guidelines will be positively received and that implementation will be widespread.

Author affiliations
[1]Leeds Institute of Health Sciences, University of Leeds, Leeds, UK
[2]Leeds Institute of Rheumatic and Musculoskeletal Medicine, University of Leeds, Leeds, UK
[3]NIHR Leeds Biomedical Research Centre, Leeds, UK
[4]Nuffield Department of Orthopaedics Rheumatology and Musculoskeletal Sciences, University of Oxford, Oxford, UK
[5]Musculoskeletal Research Unit, Bristol Medical School, University of Bristol, Southmead Hospital, Bristol, UK
[6]MRC Lifecourse Epidemiology Unit, Southampton, UK
[7]Manor Park Surgery, Leeds, UK
[8]Trauma and Orthopaedics, Weston Area Health NHS Trust, Weston-super-Mare, UK
[9]Department of Orthopaedic Surgery, Leeds Teaching Hospitals NHS Trust, Leeds, Leeds, UK

Contributors All authors contributed to the planning and design of this study, drafted the article, revised the article and approved the final manuscript.

Funding This article presents independent research funded by the National Institute for Health Research (NIHR) Health Services and Delivery Research Programme (14/70/146) and by the NIHR Leeds Biomedical Research Centre (BRC).

Disclaimer The views expressed are those of the author(s) and not necessarily those of the NIHR or the Department of Health and Social Care.

Competing interests None declared.

Patient consent for publication Not required.

Provenance and peer review Not commissioned; peer reviewed for ethical and funding approval prior to submission.

Open access This is an open access article distributed in accordance with the Creative Commons Attribution 4.0 Unported (CC BY 4.0) license, which permits others to copy, redistribute, remix, transform and build upon this work for any purpose, provided the original work is properly cited, a link to the licence is given, and indication of whether changes were made. See: https://creativecommons.org/licenses/by/4.0/.

REFERENCES

1. National Joint Registry for England, Wales. Northern Ireland and the Isle of Man: 15th Annual report. 2018 http://www.njrreports.org.uk/
2. Vanhegan IS, Malik AK, Jayakumar P, *et al*. A financial analysis of revision hip arthroplasty: the economic burden in relation to the national tariff. *J Bone Joint Surg Br* 2012;94:619–23.
3. Briggs T. *Getting it right first time. Improving the quality of orthopaedic care within the National Health Service in England*, 2012.
4. Singh JA. Epidemiology of knee and hip arthroplasty: a systematic review. *Open Orthop J* 2011;5:80–5.
5. British Orthopaedic Association. *Primary total hip replacement: a guide to good practice*, 2012.
6. Smith LK. A survey of the current state of hip arthroplasty surveillance in the United Kingdom. *Musculoskeletal Care* 2014;12:232–8.
7. Robert G, Harlock J, Williams I. Disentangling rhetoric and reality: an international Delphi study of factors and processes that facilitate the successful implementation of decisions to decommission healthcare services. *Implementation Science* 2014;9:123.
8. Higgins JPT. *Cochrane Handbook for Systematic Reviews of Interventions Version 5.1.0*: The Cochrane Collaboration, 2011. Available from. http://handbook.cochrane.org
9. Liberati A, Altman DG, Tetzlaff J, *et al*. The PRISMA statement for reporting systematic reviews and meta-analyses of studies that evaluate health care interventions: explanation and elaboration. *PLoS Med* 2009;6:e1000100.
10. Higgins JP, Altman DG, Sterne JA. Chapter 8: Assessing risk of bias in included studies in Cochrane handbook for systematic reviews of interventions Version 5.1.0. 2011 http://handbook.cochrane.org
11. Wells G, Shea B, O'Connell D, *et al*. The Newcastle-Ottawa Scale (NOS) for assessing the quality of nonrandomised studies in meta-analyses. 2014 http://www.ohri.ca/programs/clinical_epidemiology/oxford.asp;
12. Dixon-Woods M, Cavers D, Agarwal S, *et al*. Conducting a critical interpretive synthesis of the literature on access to healthcare by vulnerable groups. *BMC Med Res Methodol* 2006;6:35.
13. Drummond MF, Schulpher MJ, Claxton K, *et al*. *Methods for the Economic Evaluation of Health Care Programmes*. 4th edn. Oxford: Oxford University Press, 2015.
14. Clinical Practice Research Datalink. http://www.cprd.com
15. ResearchOne. http://www.researchone.org
16. Hospital Episode Statistics (HES). http://www.hscic.gov.uk/hes
17. Welcome from the National Joint Registry for England, Wales, Northern Ireland and the Isle of Man. http://www.njrcentre.org.uk
18. http://www.hscic.gov.uk/hesproms
19. Pinedo-Villanueva R, Turner D, Raftery JP, *et al*. Outcomes after total hip replacement. *Osteoarthritis Cartilage* 2014;22:S214.
20. Pinedo-Villanueva R, Turner D, Raftery JP, *et al*. Primary care costs attributable to osteoarthritis before total hip replacement. *Osteoarthritis Cartilage* 2014;22:S210–1.
21. Jason PF, Gray RJ. A proportional hazards model for the subdistribution of a competing risk. *Journal of the American Statistical Association* 1999;94:496–509.
22. Prieto-Alhambra D, Pagès-Castellà A, Wallace G, *et al*. Predictors of fracture while on treatment with oral bisphosphonates: a population-based cohort study. *J Bone Miner Res* 2014;29:268–74.
23. Hirsch K, Wienke A. Software for semiparametric shared gamma and log-normal frailty models: An overview. *Comput Methods Programs Biomed* 2012;107:582–97.
24. Peduzzi P, Concato J, Kemper E, *et al*. A simulation study of the number of events per variable in logistic regression analysis. *J Clin Epidemiol* 1996;49:1373–9.
25. Wallace G, Judge A, Prieto-Alhambra D, *et al*. The effect of body mass index on the risk of post-operative complications during the 6 months following total hip replacement or total knee replacement surgery. *Osteoarthritis Cartilage* 2014;22:918–27.
26. Burn E, Liddle AD, Hamilton TW, *et al*. Cost-effectiveness of unicompartmental compared with total knee replacement: a population-based study using data from the National Joint Registry for England and Wales. *BMJ Open* 2018;8:e020977.
27. Fitzpatrick R, Shortall E, Sculpher M, *et al*. Primary total hip replacement surgery: a systematic review of outcomes and modelling of cost-effectiveness associated with different prostheses. *Health Technol Assess* 1998;2:1–64.
28. Guide to the methods of technology appraisal 2013. https://www.nice.org.uk/process/pmg9/chapter/foreword
29. Basu A, Rathouz PJ. Estimating marginal and incremental effects on health outcomes using flexible link and variance function models. *Biostatistics* 2005;6:93–109.
30. Clarke P, Leal J, Kelman C, *et al*. Estimating the cost of complications of diabetes in Australia using administrative health-care data. *Value Health* 2008;11:199–206.
31. Mullahy J. Much ado about two: reconsidering retransformation and the two-part model in health econometrics. *J Health Econ* 1998;17:247–81.
32. NICE technology appraisal guidance. https://www.nice.org.uk/about/what-we-do/our-programmes/nice-guidance/nice-technology-appraisal-guidance

BMJ Open: first published as 10.1136/bmjopen-2019-031351 on 25 June 2019. Downloaded from http://bmjopen.bmj.com/ on 15 July 2019 by guest. Protected by copyright.

For peer review only - http://bmjopen.bmj.com/site/about/guidelines.xhtml

UK poSt Arthroplasty Follow-up rEcommendations (UK SAFE): What does analysis of linked, routinely collected national datasets tell us about mid-late term revision risk after knee replacement?

Journal:	*BMJ Open*
Manuscript ID	bmjopen-2020-046900.R1
Article Type:	Original research
Date Submitted by the Author:	04-Oct-2021
Complete List of Authors:	Smith, Lindsay; University of the West of England, Health & Applied Sciences<br>Garriga, Cesar; University of Oxford Nuffield Department of Orthopaedics Rheumatology and Musculoskeletal Sciences<br>Kingsbury, Sarah; Leeds Biomedical Research Centre; University of Leeds Leeds Institute of Rheumatic and Musculoskeletal Medicine<br>Pinedo-Villanueva, Rafael; University of Oxford Nuffield Department of Orthopaedics Rheumatology and Musculoskeletal Sciences<br>Delmestri, Antonella; University of Oxford Nuffield Department of Orthopaedics Rheumatology and Musculoskeletal Sciences<br>Arden, Nigel; University of Oxford, Nuffield Department of Orthopaedics, Rheumatology and Musculoskeletal Sciences (NDORMS); University of Southampton, MRC Lifecourse Epidemiology Unit<br>Stone, Martin; Leeds Teaching Hospitals NHS Trust<br>Conaghan, Philip; University of Leeds Leeds Institute of Rheumatic and Musculoskeletal Medicine<br>Judge, Andrew; University of Bristol, Translational Health Sciences, Bristol Medical School
**Primary Subject Heading**:	Surgery
Secondary Subject Heading:	Evidence based practice, Epidemiology
Keywords:	SURGERY, Adult orthopaedics < ORTHOPAEDIC & TRAUMA SURGERY, Knee < ORTHOPAEDIC & TRAUMA SURGERY, Musculoskeletal disorders < ORTHOPAEDIC & TRAUMA SURGERY

I, the Submitting Author has the right to grant and does grant on behalf of all authors of the Work (as defined in the below author licence), an exclusive licence and/or a non-exclusive licence for contributions from authors who are: i) UK Crown employees; ii) where BMJ has agreed a CC-BY licence shall apply, and/or iii) in accordance with the terms applicable for US Federal Government officers or employees acting as part of their official duties; on a worldwide, perpetual, irrevocable, royalty-free basis to BMJ Publishing Group Ltd ("BMJ") its licensees and where the relevant Journal is co-owned by BMJ to the co-owners of the Journal, to publish the Work in this journal and any other BMJ products and to exploit all rights, as set out in our licence.

The Submitting Author accepts and understands that any supply made under these terms is made by BMJ to the Submitting Author unless you are acting as an employee on behalf of your employer or a postgraduate student of an affiliated institution which is paying any applicable article publishing charge ("APC") for Open Access articles. Where the Submitting Author wishes to make the Work available on an Open Access basis (and intends to pay the relevant APC), the terms of reuse of such Open Access shall be governed by a Creative Commons licence – details of these licences and which Creative Commons licence will apply to this Work are set out in our licence referred to above.

Other than as permitted in any relevant BMJ Author's Self Archiving Policies, I confirm this Work has not been accepted for publication elsewhere, is not being considered for publication elsewhere and does not duplicate material already published. I confirm all authors consent to publication of this Work and authorise the granting of this licence.

For peer review only

UK poSt Arthroplasty Follow-up rEcommendations (UK SAFE): What does analysis of linked, routinely collected national datasets tell us about mid-late term revision risk after knee replacement?

Authors:

Smith, L.K.; Garriga, C.; Kingsbury, S.R.; Pinedo-Villanueva, R.; Delmestri, A.; Arden, N.; Stone, M.; Conaghan, P.G; Judge, A.;

Corresponding author:

Lindsay K. Smith

Postal address: University of the West of England, Faculty of Health & Applied Sciences, Rm 2G40 Glenside Campus, Blackberry Hill, Bristol BS16 1DD, UK

Email: Lindsay.smith2@nhs.net

Mobile no: 07979 007 625

Fax no: not available.

Author affiliations:

Cesar Garriga: Nuffield Department of Orthopaedics, Rheumatology and Musculoskeletal Sciences, Nuffield Orthopaedic Centre, University of Oxford, UK

Sarah Kingsbury: Leeds Institute of Rheumatic and Musculoskeletal Medicine, University of Leeds, and NIHR Leeds Biomedical Research Centre, Leeds, UK

Rafael Pinedo-Villanueva: Nuffield Department of Orthopaedics, Rheumatology and Musculoskeletal Sciences, Nuffield Orthopaedic Centre, University of Oxford, UK

Antonella Delemestri: Nuffield Department of Orthopaedics, Rheumatology and Musculoskeletal Sciences, Nuffield Orthopaedic Centre, University of Oxford, UK

Nigel Arden: Nuffield Department of Orthopaedics, Rheumatology and Musculoskeletal Sciences, Nuffield Orthopaedic Centre, University of Oxford, UK

Martin Stone: Leeds Teaching Hospitals NHS Trust, Leeds UK and NIHR Leeds Biomedical Research Centre, Leeds, UK

Philip Conaghan: Leeds Institute of Rheumatic and Musculoskeletal Medicine, University of Leeds, and NIHR Leeds Biomedical Research Centre, Leeds, UK

Andrew Judge: Translational Health Sciences, Bristol Medical School, University of Bristol, UK

Word count: 4228

Keywords: knee replacement, mid-late revision, follow-up, Cox regression

For peer review only

Abstract (292 words)

Objective. To identify patients at risk of mid-late term revision of knee replacement (KR) to inform targeted follow-up.

Design: Analysis of linked national datasets from primary and secondary care (Clinical Practice Research Datalink [CPRD-GOLD]; National Joint Registry [NJR]; English Hospital Episode Statistics [HES]; Patient Reported Outcome Measures [PROMs]).

Participants: Primary elective KRs aged ≥18.

Event of interest: Revision surgery ≥5 years (mid-late term) post-primary KR.

Statistical methods: Cox regression modelling to ascertain risk factors of mid-late term revision. Hazard ratios (HR) and 95% confidence intervals (CI) assessed association of sociodemographic factors, comorbidities, medication, surgical variables and PROMs with mid-late term revision.

Results: NJR-HES-PROMs data was available from 2008-2011 on 188,509 KR. CPRD GOLD-HES data covered 1995-2011 on 17,378 KR. Patients had minimum 5-years post primary surgery to end 2016. Age and gender distribution were similar across datasets; mean age 70 years, 57% female. In NJR there were 8,607 (4.6%) revisions, median time-to-revision post-primary surgery 1.8 years (range 0-8.8), with 1,055 (0.6%) mid-late term revisions; in CPRD GOLD, 877 (5.1%) revisions, median time-to-revision 4.2 years (range 0.02-18.3), with 352 (2.0%) mid-late term revisions.

Reduced risk of revision after 5 years was associated with older age (HR:0.95; 95%CI: 0.95-0.96), obesity (0.70; 0.56-0.88), living in deprived areas (0.71; 0.58-0.87), non-white ethnicity (0.58; 0.43-0.78), worse pre-operative pain and functional limitation (0.42; 0.33-0.53), worse 6-month post-operative pain and function (0.33; 0.26-0.41) or moderate anxiety/depression (0.73; 0.63-0.83) at primary surgery.

Increased risk was associated with male gender (1.32; 1.04-1.67); when anticonvulsants (gabapentin, pregabalin) (1.58; 1.01-2.47) or opioids (1.36; 1.08-1.71) were required prior to primary surgery.

No implant factors were identified.

Conclusion: The risk of mid-late term KR revision is very low. Increased risk of revision is associated with patient case-mix factors, and there is evidence of sociodemographic inequality.

Article summary

- This study is part of a wider programme of work to identify potential patient groups for follow-up after hip and knee replacement and used large national routine datasets from primary and secondary care
- The linkage of datasets allowed us to explore the impact of multiple risk factors on the mid-late term risk of revision of knee replacement
- This is one of the first studies to identify predictors of mid-late term revision risk for knee replacement from real-world data and contributes to the discussion on follow-up.

For peer review only

- A limitation of the NJR-HES-PROMs linked data was limited long-term follow-up due to including data from 2009 onwards but only primary operations up to 2011 to allow for revision rates after 5-years.

For peer review only

For peer review only - http://bmjopen.bmj.com/site/about/guidelines.xhtml

INTRODUCTION

Primary knee replacement (KR) surgery is a common elective orthopaedic procedure for the treatment of knee pain due to end stage osteoarthritis (OA). There is good evidence showing that KR is highly clinically effective, reducing symptoms of pain and functional limitations for the vast majority of patients[1-3] and is also cost effective[4,5]. Over 100,000 operations are carried out each year in the UK[6]. The lifetime risk of receiving knee arthroplasty in the UK is estimated to be 10.8% for women and 8.1% for men[7]. These numbers are projected to increase with an ageing and increasingly obese population, placing a growing public health burden on the NHS in respect of funding and capacity[8].

There is significant pressure on hospital trusts to reduce the amount of follow-up appointments due to expanding waiting lists, cancellation of elective surgery and increasing numbers of patients needing primary joint replacement. Although previous British Orthopaedic Association guidelines recommended outpatient follow-up at 1 and 7 years, and every 3 years thereafter, recent guidelines for primary joint replacement in the UK recommend further research on follow-up due to a lack of evidence[9,10]. There is variation across the country in how hospitals organise follow-up services and many units stopped follow-up after an early post-operative check[11]. Evidence is required on the impact that disinvestment in follow-up services may have on patient safety. There is a need to ensure early detection of patients with failing implants and target follow-up accordingly. In March 2014, the James Lind Alliance and NIHR Priority Setting Partnership for Hip and Knee Replacement for Osteoarthritis identified that defining the ideal postoperative follow-up period and the best long-term care model for people with OA and knee replacement was amongst its top ten research priorities, highlighting the importance of appropriate follow-up to ensure the health of patients.

The objective of this study was to use nationally available datasets to identify which groups of patients with KR may require follow-up based on their mid-late term revision risk (5 or more years post primary surgery). This work forms part of a larger programme of work, UK SAFE, that was designed to address the research question: Is it safe to disinvest in mid-late term follow-up of hip and knee replacement?[12]. The UK SAFE programme of work took place between 01/12/2016 and 30/11/2020 (protocol provided in online supplemental file 1).

For peer review only - http://bmjopen.bmj.com/site/about/guidelines.xhtml

METHODS

Study design

This was a nation-wide retrospective cohort study in which national data from primary care (Clinical Practice Research Datalink) and secondary care (National Joint Registry, Hospital Episode Statistics and Patient-Reported Outcome Measures) were linked to identify predictors of mid-late term revision of KR.

Sources of data

(1) CPRD GOLD-HES: The Clinical Practice Research Datalink (CPRD) GOLD comprises the entire computerised medical records of a sample of patients attending general practitioners (GPs) in the UK[13]. It contains information on over 14 million patients registered at over 700 general practices in the UK. With 4.4 million active (alive, currently registered) patients meeting quality criteria, approximately 6.9% of the UK population are included and patients are broadly representative of the UK general population in terms of age, sex and ethnicity[14]. GPs in the UK play a key role in the delivery of healthcare by providing primary care and referral to specialist hospital services, and each GP practice records this medical information for individual patients. The CPRD is administered by the Medicines and Healthcare products Regulatory Agency (MHRA). CPRD GOLD records contain all clinical and referral events in both primary and secondary care in addition to comprehensive demographic information, prescription data, and hospital admissions. Data is stored using Read codes for diseases that are cross-referenced to the International Classification of Diseases (ICD-10). Read codes are used as the standard clinical terminology system within UK primary care. Only practices that pass quality control are used as part of CPRD GOLD. CPRD ensures patient confidentiality by providing anonymised healthcare records.

CPRD GOLD data were linked to data for all-cause mortality, provided by the Office for National Statistics (ONS)[15]. CPRD GOLD data were also linked to the Index of Multiple Deprivation (IMD) and to the Hospital Episode Statistics (HES) database (described later). CPRD already provide access to HES data for England that is held under the CPRD data Linkage Scheme, available for around 60% of patients in the CPRD GOLD database. Previous research by the CPRD team has shown that linked practices/patients are representative of the CPRD GOLD population as a whole[16].

(2) NJR-HES-PROMs: Starting in 2003, the National Joint Registry (NJR) collected information on all hip and knee replacements performed each year in both public and private hospitals in England, Wales, Northern Ireland and the Isle of Man[17]. Data are entered into

For peer review only - http://bmjopen.bmj.com/site/about/guidelines.xhtml

the NJR using forms completed at the time of surgery, and revision operations are linked to primaries using unique patient identifiers. Data recorded in the NJR includes prosthesis and operative information (prosthesis type, approach, thromboprophylaxis); patient information (age, gender, body mass index (BMI), American Society of Anaesthesiologists (ASA) grade); surgeon and unit information (including caseloads, public/private status).

The HES database holds information on all patients admitted to National Health Service (NHS) hospitals in England, including diagnostic ICD codes providing information about a patient's illness or condition and NHS national clinical procedural codes (OPCS4) for surgery. It covers a smaller geographical area than the NJR and does not include privately funded operations. However, HES provides additional information for every patient (including detailed comorbidity information and deprivation indices) and about every procedure (including length of stay and need for blood transfusion or critical care). Additional records contain details of readmissions, reoperations, and revisions not recorded in the NJR database.

Since April 2009, Patient-Reported Outcome Measure data (PROMs) have been collected on all knee replacements performed in public hospitals in England[18]. A health-related quality of life questionnaire (the EuroQol five domain (EQ-5D-3L)[19]) and a joint-specific outcome score (the Oxford Knee Score (OKS)[20]) are collected pre-operatively and at 6 months after surgery, along with patient-reported measures of preoperative disability and postoperative satisfaction.

For this analysis we used NJR records linked to data from the HES and PROMs databases on all KR operations.

Participants

Anonymised records were extracted for all patients over 18 years of age receiving primary knee replacement surgery. For CPRD GOLD-HES data, the time span covered the years 1995 to 2017; for NJR-HES-PROMS data, it covered the years 2009 to 2017. Patients were included if they had primary total knee replacement or uni-compartmental knee replacement. We excluded patients that had revision surgery and total joint replacement of unspecified fixation. The following exclusions were made to remove potential case-mix issues: other injuries due to trauma, such as transport accidents and falls; non-elective admissions; a diagnosis other than primary knee OA. There will be some overlap between patients receiving knee replacement in the two data sources (around 7% of patients between 2009 and 2016); however, these anonymised datasets are analysed independently of each other.

For peer review only - http://bmjopen.bmj.com/site/about/guidelines.xhtml
For peer review only

Primary outcome

Early complications (defined as less than five years) are often symptomatic and include infection and technical errors[21]. Arthroplasty failure in the longer term (defined as after five years), constituting 50% of revision surgery, is usually caused by bearing-surface wear and associated consequences of periprosthetic osteolysis or aseptic loosening, and may be asymptomatic until clinical and radiographic failure have occurred[21,22]. The primary outcome was defined as mid-late term revision (defined as more than 5 years post primary surgery). Revision is defined as the removal, exchange or addition of any of the components of arthroplasty. In the NJR-HES-PROMS linked datasets, operative details are completed using the NJR dataset, rather than the OPCS4 coding used by the HES dataset. The NJR collects operative data using two forms, one for primary operations, and the other for revision operations. In both cases, all component labels from the surgery are attached to the form and it is from these that the component details are collected. Revision operations are linked to primaries using unique patient identifiers and so, two operations on the same knee would be linked using this system. The combination of the separate coding at source and the secondary linkage gives confidence that primary and revision operations are correctly identified. In the CPRD GOLD dataset, subjects with a revision surgery procedure are identified using the Read codes, and for those with HES-linked data OPCS4 codes can be used.

Predictors

Secondary Care Predictors: The patient level characteristics available in NJR and HES include: age, gender, BMI, area deprivation, rurality, ethnicity, Charlson co-morbidity index[23] (calculated from HES using ICD10 codes), ASA grade. Data from the NJR provide additional information on surgical and operative factors: whether or not a minimally invasive technique was used; annual surgeon volume/case load, operative time, grade of operating surgeon, surgical approach, patient position, implant fixation, type of mechanical or chemical thromboprophylaxis, unit type (public, private, independent sector treatment centre). Data from the PROMs database provides additional information on symptoms of pain, function and health related quality of life pre-operatively and at 6-months post-surgery. Pain and function are measured using the OKS. The EQ-5D-3L consists of five questions (assessing mobility, self-care, ability to conduct usual activities, degree of pain/discomfort, and degree of anxiety/depression), ranging from 1 (best state) to 3 (worst state). EQ-5D-3L can be expressed as an overall index (graded from -0.594 to 1), or as ordinal responses for each category

For peer review only - http://bmjopen.bmj.com/site/about/guidelines.xhtml

Primary Care Predictors: The CPRD GOLD database includes information on: age, gender, BMI, joint replaced (hip/knee), year of joint replacement operation, recorded diagnosis of OA (yes/no), fracture pre-surgery (yes/no), calcium and vitamin D supplements, use of bisphosphonates, use of selective oestrogen receptor modulators, oral glucocorticosteroid therapy, smoking status and alcohol intake recorded closest to the date of the primary surgery, region of UK, co-morbid conditions registered by the physician from the following list (asthma, malabsorptive syndromes, inflammatory bowel disease, hypertension, hyperlipidaemia, ischaemic heart disease, stroke, chronic obstructive pulmonary disease (COPD), chronic kidney failure, neoplasms, diabetes), use of drugs which can affect fracture risk (proton pump inhibitors, anti-arrhythmics, anticonvulsants, antidepressants, anti-Parkinson drugs, statins, thiazide diuretics, anxiolytics).

Sample size

We included all patients receiving planned elective primary surgery for knee OA. For the NJR-HES-PROMs data, this covered the years 2009 to 2016 (as our requested linked HES data was from 2008 onwards and earlier years of data were not available to us). For the CPRD GOLD-HES this spanned the years 1995 to 2016. For both datasets we excluded patients receiving a primary knee replacement after 2011 to ensure all patients had at least 5-years follow-up, as we were not interested in revisions occurring in the early period up to 5-years after the primary replacement surgery. The sample was created from all available data that satisfied these criteria.

Statistical analysis methods

Survival analysis was used to model time to revision. To identify patients most likely to require revision, proportional hazards regression modelling was used to identify pre, peri- and post-operative predictors of mid-late term revision. The date of the first incidence of a subject's knee replacement was used as the start time. The event of interest in all time-to-event models was the first recorded revision operation. Linearity of continuous predictors was assessed using fractional polynomial regression modelling. Proportionality assumptions were checked using Schoenfeld residuals. Missing data was handled by using multiple imputation methods using the ICE (Imputation by Chained Equations) procedure[24]. Standard errors were calculated using Rubin's Rules. We include all predictor variables in the multiple imputation process, together with the outcome variable (Nelson Aalen estimate of survival time and whether or not the patient had the outcome) as this carried information about missing values of the predictors.

For the CPRD GOLD-HES primary care we generated 10 imputed datasets for KR. Data

For peer review only - http://bmjopen.bmj.com/site/about/guidelines.xhtml

was imputed for the variables BMI, deprivation index, smoking and drinking risk factors. For secondary care NJR-HES-PROMS dataset we generated a single imputed dataset for KR. Variables imputed were BMI, deprivation index, rurality, ethnicity, OKS baseline scores and EQ-5D-3L item for anxiety and depression. We ran univariate Cox regression models. Risk factors with a P value<0.20 were selected for a multivariable model. Backward selection of variables was used to identify variables to keep in the final model risk factors with at least one category with a P value<0.05. For the CPRD GOLD-HES primary care dataset we present two final models, one with medication use as yes/no variables, and the other model with daily defined doses (DDDs) calculated from one year prior to the primary surgery and divided in tertiles. In addition, we conducted sensitivity analyses using a Fine-Gray competing risk model to account for the competing risk of death.

Patient and Public Involvement

Members of the National Institute for Health Research (NIHR) Leeds Biomedical Research Centre and Bristol patient and public involvement groups (PPI)were involved in developing the UK SAFE research question and work programme based on experiences of arthroplasty and preferences for care. The steering committee includes a PPI co-applicant who has contributed to interpretation of the results and will be involved in production of the final report that is disseminated to the public, patients and NHS staff.

RESULTS

This study has been reported in accordance with the STROBE checklist (online supplemental file 2).

Participants

For the CPRD GOLD-HES dataset, 64,071 sets of data were available, and Table 1 shows the steps toward 17,378 participants. Construction of the NJR-HES-PROMs dataset commenced with 841,212 records in the NJR and 188,509 participants after exclusions (Table 2).

For peer review only

For peer review only - http://bmjopen.bmj.com/site/about/guidelines.xhtml

Table 1. Stages of patient selection for inclusion in study: Primary care data.

**Included**		**Excluded**
Patients with primary knee replacement in CPRD GOLD (64,071)		
	→	Outcome (knee revision) and index event (primary surgery) outside England: 5397 (8.4%); Wales 6,982 (10.9%)
	→	Outcome (knee revision) and index event: 31,395 (42.9%)
Patients with primary knee replacement in CPRD GOLD linked to HES and used in survival analysis (22,836)		
	→	Primary surgery after 2011 (allowing for 5 years of follow up): 5,458 (23.9%)
Patient with primary knee replacement used in the survival analysis (17,378)		

Key: CPRD - Clinical Practice Research Datalink; HES – Hospital Episode Statistics

Table 2. Stages of patient selection for inclusion in study: Hospital data.

**Included**		**Excluded**
Patients with primary knee replacement in National Joint Registry (841,212)		
	→	Primary surgery before 2008 (no data available in HES) (169,776; 20.2%)
	→	Primary surgery after 2011 (allowing for 5 years of follow-up) (414,832; 49.4%)
	→	Without primary surgery date (1,037; 0.1%)
	→	A diagnosis other than primary knee osteoarthritis (2,940; 0.4%)
	→	Non-elective surgeries (535; 0.06%)
	→	Without information on type of admission (63,416; 7.5%)
Patient with primary knee replacement used in the survival analysis (188, 509)		

For peer review only - http://bmjopen.bmj.com/site/about/guidelines.xhtml

Summary statistics for patients in the CPRD GOLD-HES and the NJR-HES-PROMs linked datasets are provided (online supplemental file 3: Tables A & B). The CPRD GOLD-HES linked data covered a longer time period between 1995 to 2011; the NJR-HES-PROMs data were available 2009 to 2011. The characteristics of patients in the full CPRD dataset compared to those in the CPRD-HES linked data were similar with no evidence of any selection bias (online supplemental file 3 : Table C). Both datasets allowed a minimum of 5-years follow-up to end 2016. The age and gender distribution of patients was similar across both datasets, with a mean age of 70 at time of knee replacement and 57% female. An extensive range of patient case-mix, surgical, operative factors and primary care prescribing data was available for analysis.

The CPRD GOLD-HES dataset had a longer time-to-revision. There were 877 (5.1%) revisions, with median time-to-revision of 4.2 years (range 0.02 to 18.3 years) and 352 (2.0%) were mid-late term revisions.

In the NJR-HES-PROMs data, there were 8,607 (4.6%) knee replacement revisions with a median time-to-revision of 1.8 years (range 0 to 8.8 years); this included 1,055 (0.6%) mid-late term revisions.

Predictors of mid-late term revision

Patient demographics

Older age at the time of primary KR was associated with a lower risk of mid-late revision (Tables 3, 4). The effect of age was linear and the association was strong where, for a 1-year increase in age at surgery, the risk of outcome reduced by 5%, and this finding was consistent across the CPRD GOLD-HES and NJR-HES-PROMs datasets. The effect of gender was that males had an increased risk of mid-late revision compared to females. This was only observed in the CPRD GOLD-HES data, where males had a 24% increased risk of revision, while the effect size was weaker and non-significant in the NJR-HES-PROMs dataset.

The effect of obesity on outcome was demonstrated in the NJR-HES-PROMs dataset, where compared to those of normal BMI, underweight patients were at increased risk of revision, and obese patients at reduced risk of mid-late revision. The effect of IMD deprivation in the NJR-HES-PROMs dataset showed that patients in the most deprived areas were less likely to receive mid-late term revision; there was no such association with obesity or deprivation observed in the CPRD GOLD-HES dataset. An association with ethnicity was observed only in the NJR-HES-PROMs dataset, where patients of non-white ethnicity were less likely to be

For peer review only - http://bmjopen.bmj.com/site/about/guidelines.xhtml

revised mid-late term.

Implant factors (NJR-HES-PROMs dataset)

None of the implant related factors were associated with an increased mid-late revision risk.

Pre-operative and 6-month follow up PROMs (Table 4)

There was a clear linear trend with the pre-operative and 6-month post-operative OKS, where patients with the most pain and functional limitations at the time of surgery, and at 6-months after surgery, were substantially more likely to require mid-late term revision. Patients with pre-operative anxiety/depression were found to be less likely to receive a mid-late revision operation.

Primary care co-morbidities and medication use (Table 3)

Through the CPRD GOLD-HES dataset, we were able to investigate co-morbidities recorded prior to surgery and medication use. There was no effect of pre-operative co-morbidity for KR. With medication use, oral glucocorticoid steroid therapy was associated with a lower risk of revision whereas use of antiarrhythmics and anticonvulsants placed patients at a higher risk.

For the pain medication use, an increased revision risk was observed in those patients requiring opiates. When examining effects of medication use in more detail, by looking at DDDs calculated from the 1 year prior to the primary surgery and divided into tertiles, the effect of opioids was only significant in the highest DDD tertile of >600 DDD.

The sensitivity analysis for the competing risk of death is presented (Tables 3&4).

For peer review only

Table 3. Cox regression model identifying risk factors for revision after 5 years of primary total knee and unicompartmental replacement. Primary care data.

	Patients undergoing KR (N=17,378)			Patients undergoing KR with missing dose for bisphosphonates & opioids excluded (N=14,470)	
**Risk factors (reference category)**	**Crude analysis**	**Adjusted analysis**	**Adjusted competing risk analysis**	**Adjusted analysis**	**Adjusted competing risk analysis**
		**(Drug yes/no)**	**(Drug yes/no)**	**(Drug DDD)**	**(Drug DDD)**
	**Hazard ratio [95% CI]; P-value**	**Hazard ratio [95% CI]; P-value**	**Hazard ratio [95% CI]; P-value**	**Hazard ratio [95% CI]; P-value**	**Hazard ratio [95% CI]; P-value**
**Year of primary KR** (2010-2011)					
1995-1999	4.63 [1.98 to 10.81]; **P<0.01**	5.39 [2.28 to 12.75]; **P<0.01**	6.60 [2.82 to 15.44]; P<0.01	8.10 [2.52 to 25.98]; **P<0.01**	10.16 [3.20 to 32.29]; P<0.01
2000-2004	3.24 [1.42 to 7.41]; **P=0.01**	3.65 [1.59 to 8.40]; **P<0.01**	4.33 [1.90 to 9.87]; P<0.01	5.49 [1.73 to 17.37]; **P<0.01**	6.64 [2.12 to 20.83]; P=0.001
2005-2009	2.36 [1.04 to 5.36]; **P=0.04**	2.42 [1.06 to 5.52]; **P=0.04**	2.77 [1.22 to 6.28]; P=0.015	3.45 [1.10 to 10.86]; **P=0.03**	4.04 [1.29 to 12.65]; P=0.017
**Age at primary KR** (continuous variable)	0.93 [0.92 to 0.94]; **P<0.01**	0.93 [0.92 to 0.94]; **P<0.01**	0.93 [0.92 to 0.93] ; P<0.01	0.93 [0.92 to 0.94]; **P<0.01**	0.92 [0.92 to 0.93]; P<0.01
**Sex** (Woman)					
Man	1.26 [1.02 to 1.55]; **P=0.03**	1.24 [1.00 to 1.53]; P=0.06	1.18 [0.95 to 1.46]; P=0.13	1.32 [1.04 to 1.67]; P=**0.02**	1.26 [1.00 to 1.60]; P=0.054
**Body mass index** (normal)					
Underweight					
Overweight	1.02 [0.71 to 1.45]; P=0.93	0.97 [0.67 to 1.42]; P=0.89	1.01 [0.69 to 1.47]; P=0.96	0.98 [0.65 to 1.46]; P=0.91	1.01 [0.68 to 1.51]; P=0.97
Obese Class I (Moderately obese)	1.25 [0.86 to 1.80]; P=0.24	1.06 [0.71 to 1.57]; P=0.79	1.08 [0.73 to 1.60]; P=0.71	1.09 [0.69 to 1.70]; P=0.72	1.11 [0.71 to 1.73]; P=0.66
Obese Class II and higher	1.35 [0.91 to 2.00]; P=0.14	1.03 [0.65 to 1.63]; P=0.90	1.03 [0.65 to 1.64]; P=0.90	0.97 [0.58 to 1.63]; P=0.90	0.97 [0.57 to 1.63]; P=0.89
**Region** (East Midlands)					
East of England	0.83 [0.49 to 1.41]; P=0.49	0.95 [0.56 to 1.61]; P=0.84	0.94 [0.55 to 1.59]; P=0.82		
London	0.81 [0.46 to 1.43]; P=0.47	0.96 [0.54 to 1.71]; P=0.90	0.94 [0.53 to 1.66]; P=0.83		

For peer review only - http://bmjopen.bmj.com/site/about/guidelines.xhtml

North East	0.28 [0.08 to 0.95]; **P=0.04**	0.27 [0.08 to 0.91]; **P=0.04**	0.27 [0.08 to 0.91]; P=0.035		
North West	0.88 [0.53 to 1.47]; P=0.63	0.93 [0.56 to 1.55]; P=0.78	0.91 [0.55 to 1.52]; P=0.73		
South Central	0.81 [0.48 to 1.36]; P=0.42	0.93 [0.55 to 1.57]; P=0.79	0.91 [0.54 to 1.52]; P= 0.71		
South East Coast	1.08 [0.64 to 1.82]; P=0.77	1.37 [0.82 to 2.29]; P=0.23	1.33 [0.80 to 2.23]; P=0.28		
South West	0.86 [0.51 to 1.44]; P=0.56	1.01 [0.60 to 1.70]; P=0.97	0.98 [0.58 to 1.65]; P= 0.95		
West Midlands	0.74 [0.44 to 1.26]; P=0.26	0.79 [0.46 to 1.33]; P=0.37	0.78 [0.46 to 1.31]; P=0.34		
Yorkshire & The Humber	0.87 [0.46 to 1.65]; P=0.68	0.88 [0.47 to 1.66]; P=0.70	0.87 [0.46 to 1.63]; P= 0.67		
**Drugs prior to primary KR**					
Oral glucocorticosteroid therapy	0.75 [0.56 to 1.02]; P=0.07	0.72 [0.53 to 0.99]; **P=0.04**	0.69 [0.50 to 0.94]; P=0.02		
**Drugs which can affect fracture risk prior to primary KR**					
Anti-arrhythmics	1.35 [0.97 to 1.87]; P=0.08	1.41 [1.00 to 1.98]; **P=0.05**	1.36 [0.97 to 1.92]; P=0.078		
Anticonvulsants	1.72 [1.11 to 2.68]; **P=0.02**	1.58 [1.01 to 2.47]; **P=0.04**	1.50 [0.96 to 2.34]; P=0.076		
**Painkillers/anti-inflammatory drugs**					
Total opiates	1.40 [1.13 to 1.73]; **P<0.01**	1.36 [1.08 to 1.71]; **P=0.01**	1.32 [1.05 to 1.65]; P=0.019		
**DDDs 1 year prior to primary KR**					
**Bisphosphonates** (no dose)					
<140 DDD	0.25 [0.03 to 1.79]; P=0.17			0.40 [0.06 to 2.91]; P=0.37	0.36 [0.05 to 2.59]; P=0.31
>=140 to 340 DDD	1.47 [0.73 to 2.96]; P=0.28			2.44 [1.12 to 5.36]; **P=0.03**	2.10 [0.96 to 4.60]; P=0.063

For peer review only - http://bmjopen.bmj.com/site/about/guidelines.xhtml

>340 DDD	0.55 [0.14 to 2.21]; P=0.40		1.08 [0.26 to 4.54]; P=0.92	0.96 [0.23 to 4.06]; P=0.95
Dose missing	1.23 [0.51 to 2.95]; P=0.65			
**Opioids total** (no dose)				
<85 DDD	1.45 [0.95 to 2.21]; P=0.09		1.33 [0.86 to 2.06]; P=0.20	1.30 [0.84 to 2.01]; P=0.25
>=85 to 365 DDD	1.36 [0.97 to 1.90]; P=0.07		1.27 [0.90 to 1.79]; P=0.17	1.22 [0.86 to 1.72]; P=0.26
>365 DDD	1.85 [1.20 to 2.85]; **P=0.01**		1.67 [1.08 to 2.59]; **P=0.02**	1.53 [0.99 to 2.38]; P=0.056
Dose missing	1.28 [0.95 to 1.72]; P=0.10			

KR, total and unicompartmental knee replacement; DDD, daily defined dose; CI, confidence interval.
Hazard ratio represents number of times to have a revision after 5 years compared with the reference group. A value >1 indicates that the group has higher risk for revision.
Variables included in the final regression model are those with at least one category with a P-value<0.05 for the 10 imputed datasets in a backward selection.
Note: Body mass index and sex were force-entered into all models. “Total opiates” includes benzomorphan derivatives, diphenylpropylamine derivatives, morphinan derivatives, natural opium alkaloids, oripavine derivatives, phenylpiperidine derivatives, and other opioids.

For peer review only

For peer review only - http://bmjopen.bmj.com/site/about/guidelines.xhtml

Table 4. Cox regression model identifying risk factors of revision after 5 years of primary total knee and unicompartmental replacement. Hospital data.

Risk factors (reference category)	Patients undergoing KR (N=188,509)		
	**Crude analysis Hazard ratio [95% CI]; P-value**	**Adjusted analysis Hazard ratio [95% CI]; P-value**	**Adjusted analysis competing risks Hazard ratio [95% CI]; P-value**
**Year of primary KR** (2008)			
2009	0.91 [0.78 to 1.06]; P=0.23	0.90 [0.77 to 1.05; P=0.20	0.88 [0.75 to 1.03]; P=0.10
2010	0.82 [0.68 to 0.98]; **P=0.03**	0.82 [0.69 to 0.99] P=0.037	0.77 [0.64 to 0.92]; P=0.004
2011	0.83 [0.64 to 1.07]; P=0.15	0.83 [0.65 to 1.07]; P=0.15	0.69 [0.54 to 0.87]; P=0.002
**Age at primary KR** (continuous variable)	0.94 [0.9-0.9]; **P<0.01**	0.95 [0.95 to 0.96]; P<0.01	0.95 [0.94 to 0.95]; P<0.01
**Sex** (Women)			
Men	1.08 [1.0-1.2]; P=0.23	1.13 [0.99 to 1.28]; P=0.074	1.09 [0.95 to 1.24]; P=0.21
**Body mass index** (normal)			
Underweight	1.96 [0.96 to 4.01]; P=0.07	2.31 [1.13 to 4.73]; P=0.022	2.22 [1.08 to 4.56]; P=0.029
Overweight	1.04 [0.85 to 1.28]; P=0.68	0.91 [0.74 to 1.11]; P=0.35	0.92 [0.75 to 1.13]; P=0.45
Obese Class I (Moderately obese)	1.02 [0.83 to 1.25]; P=0.87	0.74 [0.60 to 0.91]; P=0.004	0.75 [0.61 to 0.92]; P=0.007
Obese Class II and higher	1.20 [0.96 to 1.49]; P=0.10	0.70 [0.56 to 0.88]; P=0.002	0.71 [0.56 to 0.88]; P=0.002
**IMD (quintiles), at primary KR** (less deprived 20%)			
Less deprived 20-40%	0.87 [0.72 to 1.05]; P=0.14	0.84 [0.70 to 1.01]; P=0.06	0.84 [0.70 to 1.01]; P=0.058
Less deprived 40-60%	0.91 [0.75 to 1.10]; P=0.32	0.78 [0.64 to 0.94]; P=0.01	0.77 [0.64 to 0.93]; P=0.008
More deprived 20-40%	0.94 [0.78 to 1.14]; P=0.55	0.79 [0.65 to 0.96]; P=0.016	0.78 [0.64 to 0.94]; P=0.01
Most deprived 20%	0.87 [0.71 to 1.06]; P=0.17	0.71 [0.58 to 0.87]; P=0.001	0.70 [0.58 to 0.86] 0.001
**Ethnicity** (white)			
Non-white	0.68 [0.5-0.9]; **P=0.01**	0.58 [0.43 to 0.78]; P<0.01	0.59 [0.44 to 0.80]; P=0.001
**OKS, baseline score** (0 to 10 points)			
[0=poor, 48=good]			
(11 to 14 points)	0.82 [0.7-1.0]; **P=0.03**	0.85 [0.70 to 1.02]; P=0.073	0.85 [0.71 to 1.02]; P=0.087

For peer review only

For peer review only - http://bmjopen.bmj.com/site/about/guidelines.xhtml

(15 to 19 points)	0.69 [0.6-0.8]; **P<0.01**	0.71 [0.60 to 0.85]; P<0.01	0.73 [0.61 to 0.87]; **P<0.01**
(20 to 24 points)	0.51 [0.4-0.6]; **P<0.01**	0.55 [0.44 to 0.68]; P<0.01	0.56 [0.45 to 0.69]; **P<0.01**
(25 to 48 points)	0.37 [0.3-0.5]; **P<0.01**	0.42 [0.33 to 0.53]; P<0.01	0.43 [0.34 to 0.54]; **P<0.01**
**OKS, 6-month score** (0 to 10 points) [0=poor, 48=good]			
(11 to 14 points)	0.72 [0.61 to 0.86]; P<0.01	0.81 [0.67 to 0.96]; P=0.016	0.81 [0.68 to 0.97]; P=0.019
(15 to 19 points)	0.53 [0.44 to 0.63]; P<0.01	0.59 [0.49 to 0.72]: P<0.01	0.60 [0.50 to 0.72]; P<0.01
(20 to 24 points)	0.43 [0.35 to 0.52]; P<0.01	0.48 [0.39 to 0.59]; P<0.01	0.48 [0.39 to 0.59]; P<0.01
(25 to 48 points)	0.29 [0.23 to 0.36]; P<0.01	0.33 [0.26 to 0.41]; P<0.01	0.33 [0.26 to 0.42]; P<0.01
**EQ-5D-3L Anxiety Depression, 3 months or closer to primary KR** (I am not anxious or depressed)			
I am moderately anxious or depressed	1.02 [0.9-1.2]; P=0.78	0.73 [0.63 to 0.83]; P<0.01	0.72 [0.63 to 0.82]; P<0.01
I am extremely anxious or depressed	1.26 [0.9-1.7]; P=0.14	0.67 [0.49 to 0.91]; P=0.01	0.65 [0.48 to 0.89]; P=0.007

Key: KR, total and unicompartmental knee replacement; IMD, Index of Multiple Deprivation; CI, confidence interval; OKS, Oxford Knee Score.

Hazard ratio represents number of times to have a revision after 5 years compared with the reference group. A value >1 indicates that the group has higher risk for revision.

Variables included in the final regression model are those with at least one category with a P-value<0.05 for a single imputed dataset in a backward selection.

Note: Body mass index and sex were force-entered into all models.

For peer review only

DISCUSSION

The risk of a mid-late revision operation (≥5-years) after primary knee replacement surgery is very low. Within our CPRD GOLD-HES primary care dataset, we had up to 20-years patient follow-up from the start point of 5-years after the primary operation and even then, the mid-late revision rate was only 2.0%. In this study, it was the patient case-mix factors that were associated with mid-late term revision surgery. Patients at increased risk were those who were younger, male gender, not obese, living in affluent areas, of white ethnicity, not anxious or depressed at primary surgery. Those with worse pain and functional scores at primary surgery were at higher risk for mid-late revision than those with better scores.

Strengths of this study include the use of large national routine datasets where the NJR data are mandatory and have near complete coverage, and the CPRD GOLD data is nationally representative in respect of UK population demographic characteristics. Large sample sizes afforded us the ability to identify predictors of a rare long-term outcome such as revision surgery. A limitation of the NJR-HES-PROMs linked data was limited long-term follow-up due to including data from 2009 onwards but only primary operations up to 2011 to allow for revision rates after 5-years. This was to allow us to explore the impact of pre-operative PROMs data which has only been collected since 2009. Strengths of NJR data are detailed surgical and hospital factors available in the data. A limitation is that there have been changes in anaesthesia and surgical techniques over time that may no longer reflect current orthopaedic practice. A strength of our CPRD GOLD dataset was over 20-years of follow-up, and the ability to capture a wide range of primary and hospital factors. There were missing data for some of the variables in our data and this required us to use imputation to account for this in our analyses.

One of the aims of our study was to provide an evidence base for any group of patients in need of routine follow-up after KR. Our findings were consistent with a previous study using data from the CPRD GOLD in which the authors demonstrated an instantaneous risk of revision (risk of revision following a given period of implant survival) by age and gender subgroups[25]. The smoothed hazard plots consistently showed higher revision risks for men and younger patients at all timepoints. These graphs also showed that the trends in time to revision surgery were similar across all age bands, except for the most elderly patient groups in whom follow-up is limited by life expectancy. Males and younger patients were at a consistently higher revision risk over the whole follow-up, and these factors did not influence timing of when revision occurred. In our previous work, we have shown that younger age, males and obesity are risk factors for revision hip and knee replacement[26]. Our finding in respect of age is consistent with this existing literature, as is the effect of males, showing

For peer review only

that these effects are also seen in mid-late term, and the results were unchanged by the competing risk of death. For obesity, the opposite effect was seen in the present study where this now had a protective effect on risk of mid-late revision, although the cause of this effect was not clear. With regard to deprivation, it has previously been shown that those in the most deprived areas are less likely to receive revision knee replacement surgery,[27] and this is disappointingly consistent with what we observed and may reflect inequalities in access to revision surgery. Alternatively, it could be that obese patients or those of non-white ethnicity are more likely to be having revision surgery in the early term at less than 5-years, and hence these groups are under-represented for mid-late term revisions .However, the effect of deprivation and that of obesity were only present in the secondary care dataset, which requires further investigation.

There have been previous studies looking at the effects of medication use on revision risk, particularly for medications associated with bone and fracture risk. It has been suggested that post-operative statin use reduces revision risk for hip replacement[28]. The effects seen here in our study showed that, in crude unadjusted analyses, statins reduced the risk for knee replacement revision, but this was attenuated in the full regression model, which may be explained by the association of statin use with obesity. Bisphosphonate use has also been suggested to reduce revision risk,[29] but we saw an opposite effect for high DDD users. They had increased revision risk, which may be associated with the reason for revision as Danish studies have shown that, although bisphosphonates reduced overall all-cause revision, the risk of revision for infection was increased[30].

The findings of this study suggest that patients receiving a mid-late revision surgery are a healthier, affluent group of patients of white ethnicity. It is unclear to what extent this represents need for revision surgery as this group may be more active, healthier, with lifestyle effects; or, is this a reflection of the known measurement error in using revision surgery as an outcome measure for the success of surgery? This patient group may simply be better able to navigate the care pathway (as for the primary operation), or reflect biases in patient-surgeon decision making, and may not be representative of those requiring revision surgery. There will always be patients in pain and functional difficulty that do not seek help from their GP or surgeon. It is of major interest to better understand why patient demographic characteristics seem to play a role in knee revision surgery.

The findings in respect of pain were interesting: in the secondary care data, although pain and function at or 6-months after primary surgery were associated with reduced risk of revision, those with the poorest scores were more likely to undergo revision. In primary care

For peer review only - http://bmjopen.bmj.com/site/about/guidelines.xhtml

data, pre-operative pain medication was the only risk factor of interest other than healthy patient case-mix selection effects that are unlikely to be informative for extended follow-up. Use of oral glucocorticoid steroid therapy may be a surrogate marker for chronic health conditions and was associated with a lower risk of revision in our data. This may reflect reduced functional goals or expectations in this patient group, with less likelihood of proceeding to revision surgery, or a reluctance to proceed with surgery due to an increased risk of infection. Anticonvulsants (gabapentin, pregabalin) and opioid use pre-operatively were associated with an increased mid-late revision risk. Although opioids may be recommended for controlling pain due to osteoarthritis before primary surgery,[31] they may also be indicative of chronic pain and/or opioid related comorbidities, and 2/3rds of patients have been shown to continue to use opioids post-surgery[3229]. This group of patients often experience a mixed picture of pain and may have high levels of dissatisfaction after surgery, leading them to seek further surgical solutions for persistent pain[33,34]. Use of anticonvulsants prior to primary surgery is suggestive of existing neuropathic pain or multi-site joint pain. Post-surgery, this group of patients may experience sensitisation subsequent to chronic pain and/or additional neuropathic components, leading to more severe symptoms which places them at greater risk of revision. Further work would be required to investigate whether patients with neuropathic or chronic pain after primary KR would benefit from closer monitoring and follow-up, particularly if they are then at further increased risk of mid-late revision.

In this study, there was an opportunity to examine unique datasets for predictors of mid-late term revision risk for KR surgery. We have reported the results for KR in this study, but it is of interest that in the wider programme of work (UK SAFE[12]), the predictors of revision were different for hips and knees with age being the main consistent finding. The patient factors we identified as predictive of mid-late term revision risk after KR may reflect inequalities in access to revision surgery, or there may be other factors not captured within this study; this requires further investigation. In addition, further work is needed to determine if targeted follow-up is required for those patients with worse pain and function pre-and/or post-primary surgery, or higher levels of pre-operative pain medication (opioids and anticonvulsants) due to their increased risk of mid-late term revision. The findings from this study have implications for future provision of follow-up services for patients with a KR.

For peer review only - http://bmjopen.bmj.com/site/about/guidelines.xhtml

Acknowledgements

We gratefully acknowledge interpretation offered by Mr. Nicholas Howells (North Bristol NHS Trust). We would like to thank the patients and staff of all the hospitals in England and Wales who have contributed data to the National Joint Registry (NJR); and the Healthcare Quality Improvement Partnership (HQIP), the NJR Steering Committee and staff at the NJR Centre for facilitating this work. The authors have conformed to the NJR's standard protocol for data access and publication.

Competing interests statement

LKS reports grants from National Institute for Health Research during the conduct of the study. AJ reports grants from National Institute for Health Research and has received consultancy fees from Freshfields Bruckhaus Derringer and Anthera Pharmaceuticals LTD unrelated to this work. AJ was supported by the NIHR Biomedical Research Centre at University Hospitals Bristol and Weston NHS Foundation Trust and the University of Bristol. NA reports grants from Merck, personal fees from Pfizer/Lilly, unrelated to the submitted work. SRK, MS and PGC were supported in part by the NIHR Leeds Biomedical Research Centre.

Funding statement

This article presents independent research funded by the National Institute for Health Research (NIHR) Health Services and Delivery Research Programme (14/70/146) and by the NIHR Leeds Biomedical Research Centre (BRC). SRK, MS and PGC were supported in part by the NIHR Leeds Biomedical Research Centre. AJ was supported by the NIHR Biomedical Research Centre at University Hospitals Bristol and Weston NHS Foundation Trust and the University of Bristol.

Disclaimer

The views expressed represent those of the authors and do not necessarily reflect those of the NIHR, the NHS, the Department of Health and Social Care, the National Joint Registry Steering Committee or the Healthcare Quality Improvement Partnership (HQIP) who do not vouch for how the information is presented. HES data copyright © 2016, re-used with the permission of the Health & Social Care Information Centre. All rights reserved.

Ethical approval

The CPRD Group has obtained ethical approval from a National Research Ethics Service Committee (NRES) for all purely observational research using anonymised CPRD data;

For peer review only

namely, studies which do not include patient involvement. The study has been approved by ISAC (Independent Scientific Advisory Committee) for MHRA Database Research (protocol number 11_050AMnA2RA2).

For the NJR, before Personal Data and Sensitive Personal Data is recorded, express written patient consent is provided. The NJR records patient consent as either 'Yes', 'No', or 'Not Recorded'. With support under Section 251 of the NHS Act 2006, the Ethics and Confidentiality Committee (ECC), (now the Health Research Authority Confidentiality Advisory Group) allows the NJR to collect patient data where consent is indicated as 'Not Recorded'. Section 251 support (17/CAG/0030) and NHS Digital approval (DARS-NIC-172121-G0Z1H-v0.11) were obtained for the NJR-HES-PROMS dataset analysis.

Data sharing statement

Access to data is available from the National Joint Registry for England and Wales, Northern Ireland and the Isle of Man, but restrictions apply to the availability of these data, which were used under license for the current study, and so are not publicly available. Data access applications can be made to the National Joint Registry Research Committee. Access to linked HES and PROMs data is available through data applications to NHS Digital.

Original protocol

Czoski Murray CJ, *et al*. *BMJ Open* 2019;9:e031351. doi:10.1136/bmjopen-2019-031351(provided as online supplemental file 1).

Author's contributions

LKS, SRK, NA, MS, PGC, AJ in conception of work, approval of final version and accountability.

CG, AD, AJ analysis of data

AJ, SRK, MS, LKS, R P-V, PGC in interpretation of results

AJ, LKS drafted and revised final manuscript

For peer review only - http://bmjopen.bmj.com/site/about/guidelines.xhtml

References

1. Ethgen O, Bruyere O, Richy F, et al. Health-Related Quality of Life in Total Hip and Total Knee Arthroplasty. A Qualitative and Systematic Review of the Literature. *Journal of Bone and Joint Surgery* 2004;86(5):963-74.
2. Harris WH, Sledge CB. Total Hip and Total Knee Replacement (1). *New England Journal of Medicine* 1990;323(11):725-31. doi: 10.1056/nejm199009133231106
3. Harris WH, Sledge CB. Total Hip and Total Knee Replacement (2). *New England Journal of Medicine* 1990;323(12):801-07. doi: 10.1056/nejm199009203231206
4. Dakin H, Gray A, Fitzpatrick R, et al. Rationing of total knee replacement: a cost-effectiveness analysis on a large trial data set. *BMJ Open* 2012;2(1):e000332. doi: 10.1136/bmjopen-2011-000332 [published Online First: 2012/02/01]
5. Daigle ME, Weinstein AM, Katz JN, et al. The cost-effectiveness of total joint arthroplasty: a systematic review of published literature. *Best Pract Res Clin Rheumatol* 2012;26(5):649-58. doi: 10.1016/j.berh.2012.07.013
6. National Joint Registry. National Joint Registry for England, Wales, Northern Ireland and the Isle of Man. 17th Annual Report., 2020. Available at: https://reports.njrcentre.org.uk/. [Last accessed 23 October 2020].
7. Culliford DJ, Maskell J, Kiran A, et al. The lifetime risk of total hip and knee arthroplasty: results from the UK general practice research database. *Osteoarthritis Cartilage* 2012;20(6):519-24. doi: 10.1016/j.joca.2012.02.636 [published Online First: 2012/03/08]
8. Culliford D, Maskell J, Judge A, et al. Future projections of total hip and knee arthroplasty in the UK: results from the UK Clinical Practice Research Datalink. *Osteoarthritis Cartilage* 2015;23(4):594-600. doi: 10.1016/j.joca.2014.12.022 [published Online First: 2015/01/13]
9. British Orthopaedic Association. 2017 Commissioning guide: Painful osteoarthritis of the knee. Available at: https://www.boa.ac.uk/standards-guidance/commissioning-guides.html. [Last accessed 26 August 2020].
10. National Institute for Health and Care Excellence (NICE). Joint replacement (primary): hip, knee and shoulder. NG157. 04 June 2020. Available at: https://www.nice.org.uk/guidance/ng157/chapter/Recommendations#long-term-care. [Last accessed 26 August 2020].
11. Smith, L.K. Assessment of the current state of hip arthroplasty surveillance in the UK. *Musculoskeletal Care,* 2014; 12 (4) 232-238. https://doi.org/10.1002/msc.1077
12. National Institute for Health Research (NIHR). Towards UK poSt Arthroplasty Follow-up rEcommendations: UK SAFE. HS&DR-14/70/146. 20 June 2017. Available at: https://www.journalslibrary.nihr.ac.uk/programmes/hsdr/1470146/#/ [Last accessed 30 September 2021].
13. Medicines and Healthcare Products Regulatory Agency. Release Notes – CPRD GOLD February 2017 . Available at: https://cprdcw.cprd.com/_docs/Release_Notes_February2017.pdf. [Last accessed 18 October 2020].
14. Herrett E, Gallagher A, Bhaskaran K, et al. Data resource profile: Clinical Practice Research Datalink (CPRD). *International Journal of Epidmiology* 2015;44(3):827-836. doi:10.1093/ije/dyv098.15. Delmestri A, Prieto-Alhambra D. CPRD GOLD and linked ONS mortality records: reconciling guidelines, *International Journal of Medical*

For peer review only - http://bmjopen.bmj.com/site/about/guidelines.xhtml

Informatics 2020; 136:104038:1-6. Available at: https://doi.org/10.1016/j.ijmedinf.2019.104038. [Last accessed 23 October 2020].

16. Padmanabhan S, Carty L, Cameron E, et al. Approach to record linkage of primary care data from Clinical Practice Research Datalink to other health-related patient data: overview and implications. *European Journal of Epidemiology* 2019;34(1):91-99. doi: 10.1007/s10654-018-0442-4
17. National Joint Registry for England, Wales, Northern Ireland and the Isle of Man. Available at: http://www.njrcentre.org.uk/ [Last accessed 26 August 2020].
18. NHS Digital. Patient Reported Outcome Measures (PROMs). Available at: https://digital.nhs.uk/data-and-information/data-tools-and-services/data-services/patient-reported-outcome-measures-proms [Last accessed 26 August 2020].
19. EuroQol Research Group. EQ-5D. Available at : https://euroqol.org/. [Last accessed 18 October 2020].
20. Dawson J, Fitzpatrick R, Murray D, et al. Questionnaire on the perceptions of patients about total knee replacement. *J Bone Joint Surg Br* 1998;80(1):63-69.
21. Clohisy J, Calvert G, Tull F, et al. Reasons for revision hip surgery: a retospective review. *Clin Orthop Relat Res* 2004; 429(12):188-92.
22. Engh C, Jr., Hopper R, Engh C, et al. Wear-through of a modular polyethylene liner: four case reports. *Clin Orthop Relat Res 2001;383(2):175-82.*
23. Charlson ME, Pompei P, Ales KL, et al. A new method of classifying prognostic comorbidity in longitudinal studies: development and validation. *Journal of chronic diseases* 1987;40(5):373-83. [published Online First: 1987/01/01]
24. Sterne JA, White IR, Carlin JB, et al. Multiple imputation for missing data in epidemiological and clinical research: potential and pitfalls. *Bmj* 2009;338:b2393. doi: 10.1136/bmj.b2393 [published Online First: 2009/07/01]
25. Bayliss LE, Culliford D, Monk AP, et al. The effect of patient age at intervention on risk of implant revision after total replacement of the hip or knee: a population-based cohort study. *Lancet* 2017;389(10077):1424-30. doi: 10.1016/s0140-6736(17)30059-4 [published Online First: 2017/02/18]
26. Culliford D, Maskell J, Judge A, et al. A population-based survival analysis describing the association of body mass index on time to revision for total hip and knee replacements: results from the UK general practice research database. *BMJ Open* 2013;3(11):e003614. doi: 10.1136/bmjopen-2013-003614
27. Dixon T, Shaw M, Ebrahim S, et al. Trends in hip and knee joint replacement: socioeconomic inequalities and projections of need. *Annals of the Rheumatic Diseases* 2004;63(7):825. doi: 10.1136/ard.2003.012724
28. Thillemann TM, Pedersen AB, Mehnert F, et al. The risk of revision after primary total hip arthroplasty among statin users: a nationwide population-based nested case-control study. *J Bone Joint Surg Am* 2010;92(5):1063-72. doi: 10.2106/jbjs.H.01805 [published Online First: 2010/05/05]
29. Prieto-Alhambra D, Javaid MK, Judge A, et al. Association between bisphosphonate use and implant survival after primary total arthroplasty of the knee or hip: population based retrospective cohort study. *BMJ* 2011;343:d7222. doi: 10.1136/bmj.d7222 [published Online First: 2011/12/08]
30. Thillemann TM, Pedersen AB, Mehnert F, Johnsen SP, Soballe K. Postoperative use of bisphosphonates and risk of revision after primary total hip arthroplasty: A nationwide

For peer review only

population-based study. *Bone* 2010; 46(4): 946-951. doi.org/10.1016/j.bone.2010.01.377.

31. National Institute for Health and Clinical Excellence. Osteoarthritis: care and management (CG177). 12 February 2014. Available at: https://www.nice.org.uk/guidance/cg177. {Last accessed 20 October 2020].
32. Inacio MCS, Hansen C, Pratt NL, et al. Risk factors for persistent and new chronic opioid use in patients undergoing total hip arthroplasty: a retrospective cohort study. *BMJ Open* 2016;6(4):e010664. doi: 10.1136/bmjopen-2015-010664.
33. Wylde V, Beswick A, Bruce J, Blom A, Howells N, Gooberman-Hill R. Chronic pain after total knee arthroplasty. *EFORT Open Reviews*, 2018. doi.org/10.1302/2058-5241.3.180004.
34. Murray JR, Howells N, Mathews JA, Kalson NS, Porteous AJ. Investigation of the problematic knee replacement. *The Knee*, 2020. https://doi.org/10.1016/j.knee.2020.07.083

For peer review only

Abbreviations

ASA	American Society of Anaesthesiologists
BMI	Body Mass Index
COPD	Chronic obstructive pulmonary disease
CPRD	Clinical Practice Research Datalink
DDD	Daily defined dose
EQ-5D-3L	EuroQol five domain
GP	General Practitioner
HES	Hospital Episode Statistics
ICD	International Classification of Diseases
ICE	Imputation by Chained Equations
IMD	Index of Multiple Deprivation
KR	Knee replacement
MHRA	Medicines and Healthcare products Regulatory Agency
NHS	National Health Service
NIHR	National Institute for Health Research
NJR	National Joint Registry of England, Wales, Northern Ireland and the Isle of Man
OA	Osteoarthritis
OKS	Oxford Knee Score
ONS	Office for National Statistics
OPCS4	NHS national clinical procedural codes
PPI	Patient and public involvement
PROMs	Patient-reported outcome measure data
UK	United Kingdom

For peer review only

For peer review only - http://bmjopen.bmj.com/site/about/guidelines.xhtml

Supplementary files

Supplemental file I: UK SAFE protocol

Supplemental file II: STROBE checklist

Supplemental file III: Additional results

Table A. Descriptive statistics for the CPRD GOLD-HES linked datasets

Table B. Descriptive statistics for the NJR-HES-PROMs linked dataset

Table C. Descriptive statistics comparing patients in the full CPRD dataset to those with linked HES data for the CPRD-HES linked datasets

For peer review only

Open access Protocol

BMJ Open

Towards UK poSt Arthroplasty Follow-up rEcommendations (UK SAFE): protocol for an evaluation of the requirements for arthroplasty follow-up, and the production of consensus-based recommendations

Carolyn J Czoski Murray,[1] Sarah R Kingsbury,[2,3] Nigel K Arden,[4] Jenny Hewison,[1] Andrew Judge,[5,6] Jamie Matu,[1,2] Jamie O'Shea,[7] Rafael Pinedo-Villanueva,[4] Lindsay K Smith,[8] Chris Smith,[1] Christine M Thomas,[2,3] Robert M West,[1] Judy M Wright,[1] Philip G Conaghan,[2,3] Martin H Stone[3,9]

To cite: Czoski Murray CJ, Kingsbury SR, Arden NK, *et al*. Towards UK poSt Arthroplasty Follow-up rEcommendations (UK SAFE): protocol for an evaluation of the requirements for arthroplasty follow-up, and the production of consensus-based recommendations. *BMJ Open* 2019;**9**:e031351. doi:10.1136/bmjopen-2019-031351

► Prepublication history and additional material for this paper are available online. To view these files, please visit the journal online (http://dx.doi.org/10.1136/bmjopen-2019-031351).

PGC and MHS contributed equally.
CJCM and SRK contributed equally.

Received 29 April 2019
Revised 21 May 2019
Accepted 22 May 2019

Check for updates

© Author(s) (or their employer(s)) 2019. Re-use permitted under CC BY. Published by BMJ.

For numbered affiliations see end of article.

Correspondence to
Professor Philip G Conaghan; p.conaghan@leeds.ac.uk

ABSTRACT

Introduction Hip and knee arthroplasties have revolutionised the management of degenerative joint diseases and, due to an ageing population, are becoming increasingly common. Follow-up of joint prostheses is to identify problems in symptomatic or asymptomatic patients due to infection, osteolysis, bone loss or potential periprosthetic fracture, enabling timely intervention to prevent catastrophic failure at a later date. Early revision is usually more straight-forward surgically and less traumatic for the patient. However, routine long-term follow-up is costly and requires considerable clinical time. Therefore, some centres in the UK have curtailed this aspect of primary hip and knee arthroplasty services, doing so without an evidence base that such disinvestment is clinically or cost-effective.

Methods Given the timeline from joint replacement to revision, conducting a randomised controlled trial (RCT) to determine potential consequences of disinvestment in hip and knee arthroplasty follow-up is not feasible. Furthermore, the low revision rates of modern prostheses, less than 10% at 10 years, would necessitate thousands of patients to adequately power such a study. The huge variation in follow-up practice across the UK also limits the generalisability of an RCT. This study will therefore use a mixed-methods approach to examine the requirements for arthroplasty follow-up and produce evidence-based and consensus-based recommendations as to how, when and on whom follow-up should be conducted. Four interconnected work packages will be completed: (1) a systematic literature review; (2a) analysis of routinely collected National Health Service data from five national data sets to understand when and which patients present for revision surgery; (2b) prospective data regarding how patients currently present for revision surgery; (3) economic modelling to simulate long-term costs and quality-adjusted life years associated with different follow-up care models and (4) a Delphi-consensus process, involving all stakeholders, to develop a policy document which includes a stratification algorithm to determine appropriate follow-up care for an individual patient.

Ethics and Dissemination Favourable ethical opinion has been obtained for WP2a (RO-HES) (220520) and WP2B (220316) from the National Research Ethics Committee. Following advice from the Confidentiality Advisory Group (17/CAG/0122), data controllers for the data sets used in WP2a (RO-HES) – NHS Digital and The Phoenix Partnership – confirmed that Section 251 support was not required as no identifiable data was flowing into or out of these parties. Application for approval of WP2a (RO-HES) from the Independent Group Advising on the Release of Data (IGARD) at NHS Digital is in progress (DARS-NIC-147997). Section 251 support (17/CAG/0030) and NHS Digital approval (DARS-NIC-172121-G0Z1H-v0.11) have been

Strengths and limitations of this study

- ► Our mixed-methods approach allows us to address a question that would not be feasible to answer with a randomised controlled trial.
- ► Our study will capture data from a mixture of teaching hospitals, district general hospitals and hospitals with a special interest in joint replacement and with a geographical spread, increasing the generalisability of our results.
- ► Our economic model will be populated with routinely collected National Health Service (NHS) data of patients attending primary and hospital care in the UK, ensuring that our analysis is based on actual patient use of services, outcomes such as health-related quality of life and costs to the NHS.
- ► While our analysis is based on data sources that reflect clinical practice in England only, we believe key cost-effectiveness findings are likely to be informative for decision-making in the whole of the UK.

For peer review only - http://bmjopen.bmj.com/site/about/guidelines.xhtml

BMJ Open: first published as 10.1136/bmjopen-2019-031351 on 25 June 2019. Downloaded from http://bmjopen.bmj.com/ on 15 July 2019 by guest. Protected by copyright.

obtained for WP2a (NJR-HES-PROMS). ISAC (11_050MnA2R2) approval has been obtained for WP2a (CPRD-HES).

INTRODUCTION

Arguably, total hip arthroplasty (THA) and total knee arthroplasty (TKA) are the most successful surgical interventions performed in modern times. Due to an ageing population, and an obesity epidemic, hip and knee replacement procedures increase annually, rising from less than 20 000/year in the UK in 1978 to around 200 000/year in 2017.[1] The current follow-up requirements are estimated at 500 000–1 000 000 annual outpatient attendances. With limitless resources, every patient undergoing a joint arthroplasty would incur routine lifetime follow-up. The rationale for follow-up is to ensure timely detection of complications or arthroplasty failure, such as aseptic loosening, osteolysis and potential periprosthetic fracture. The cost of revision for aseptic loosening is 35% lower than that for periprosthetic fractures and has a lower incidence of complications which impact recovery.[2] However, while routine long-term follow-up of joint prostheses may support timely revision for patients with asymptomatic complications, improving long-term health outcomes, it is also costly both clinically and financially.

Orthopaedic services are already one of the poorest performers across the National Health Service (NHS) by failing to meet waiting list targets, with an estimated 8000 orthopaedic NHS breaches each month.[3] With a rapidly ageing population and medical advances that mean less stringent criteria for surgery eligibility,[4] there is no sign that demand will recede in coming years and orthopaedic services will soon reach breaking point. To reduce the burden on orthopaedic services, evidence-based consensus guidelines are required to establish how, when and on whom follow-up should be conducted.

British Hip Society (BHS) and British Orthopaedic Association (BOA) guidelines recommend outpatient follow-up at 1 and 7 years, and every 3 years thereafter for Orthopaedic Data Evaluation Panel 10A (ODEP-10A) implants, with more frequent follow-up for novel implants.[5] However, recent work revealed considerable diversity across the UK in arthroplasty follow-up pathways, in timing, how follow-up is conducted and which health professionals are involved.[6] While some centres followed-up patients beyond 10 years, others did not have an established follow-up policy and in some centres follow-up services have been curtailed or stopped entirely after an early postoperative check.[6] Notably, we do not know whether long-term follow-up is cost-effective or whether disinvestment is safe for patients.

This project aims to determine the consequences of disinvestment in hip and knee arthroplasty follow-up. Given the timeline from joint replacement to revision, with a 7% revision rate for THA and 4% revision rate for TKA at 14 years, conducting a randomised controlled trial to address this question is not feasible. Moreover, the huge variation in follow-up practice across the UK limits the generalisability of the results of an RCT. We will therefore use a mixed-methods approach to comprehensively evaluate the requirements for arthroplasty follow-up and will use this evidence to inform the development of consensus-based recommendations and a policy document which includes a stratification algorithm to determine appropriate follow-up for individual patients. Disinvestment is a complex and often contentious issue. We plan to make use of published recommendations[7] to ensure that the results of this work are understood and considered as a genuine attempt to use the best evidence available to ensure that the NHS gets value for money and that patients remain safe.

METHODS AND ANALYSIS

Study objectives

A. Identify who needs follow-up and when this should occur for primary THA, TKA and unicompartmental knee arthroplasty (UKA) surgery by making use of routinely collected NHS data.
B. Understand the patient journey (in primary and secondary care) to revision surgery by recruiting patients admitted for elective and emergency hip and knee revision surgery.
C. Establish how and when patients are identified for revision, why some patients are missed from regular follow-up and present acutely with fracture around the implant (periprosthetic fracture), by using prospective and retrospective data.
D. Identify the most appropriate and cost-effective follow-up pathway to minimise potential harm to patients by undertaking cost-effectiveness modelling.
E. Provide evidence-based and consensus-based recommendations on how follow-up of primary THA and TKA should be conducted.

Design

This is a mixed-methods study using a variety of data sources consisting of four interconnected work packages (WP): (1) a systematic literature review; (2a) analysis of routinely-collected NHS data to understand when and which patient present for revision surgery; (2b) prospective data regarding how patients currently present for revision surgery collected on around 455 patients prior to elective or emergency revision surgery; (3) economic modelling to simulate long-term costs and quality adjusted life years associated with different follow-up models; (4) a Delphi-consensus process, incorporating all previous WPs and involving all stakeholders, to develop a policy document which includes a stratification algorithm to determine appropriate follow-up for an individual patient.

WP1: systematic review

The aim of the review is to evaluate different models of routine long-term follow-up care after TKA/THA/UKA. This systematic review will establish a robust evidence base

BMJ Open: first published as 10.1136/bmjopen-2019-031351 on 25 June 2019. Downloaded from http://bmjopen.bmj.com/ on 15 July 2019 by guest. Protected by copyright.

For peer review only

for the cost-effectiveness modelling (WP3) and consensus guideline development (WP4).

Registration
This systematic review will be undertaken following Cochrane Collaboration methods[8] and reported in accordance with Preferred Reporting Items for Systematic Review and Meta-analyses guidelines.[9] It has been prospectively registered with PROSPERO (CRD42017053017).

Searches
A comprehensive literature search will be undertaken with the aim of retrieving all relevant literature, published or unpublished, which evaluated the effectiveness of long-term follow-up after primary TKA/THA/UKA. A range of information sources will be searched: BIOSIS, CINAHL, ClinicalTrials.gov, The Cochrane Library, Embase, Health Management Information Consortium, IDEAS (RePEC), Ovid Medline(R), ProQuest Dissertations and Theses, PsycINFO, PubMed and Web of Science. Reference lists of included studies will be reviewed for potentially relevant articles. A sample search strategy is detailed in the online supplementary appendix A. No date or language restrictions will be applied.

Criteria for selection of studies
All study designs will be included which (1) consider the clinical and/or cost effectiveness of routine long-term (>5 years) follow-up care after primary THA, TKA or UKA; (2) describe patient safety issues associated with routine follow-up or (3) consider the acceptability of new care pathways from the perspective of the patient and/or practitioner. Studies will be excluded if they do not report specific patient-related outcome measures or appropriate health utility measures.

Selection of studies
Titles/abstracts of identified studies will be screened for eligibility by one experienced reviewer with a random selection (25%) independently screened by a second. Potential studies will be retrieved in full text and reviewed against the inclusion/exclusion criteria independently by the same two reviewers, with a third reviewer used to settle any disputes.

Data extraction
Data will be extracted by a single reviewer using a standardised proforma capturing (1) purpose and design; (2) methodological characteristics; (3) information relating to quality assessment and (4) outcome data relating to the clinical and cost-effectiveness of routine long-term follow-up care.

Quality assessment
The Cochrane Risk of Bias assessment tool will be used for experimental studies,[10] and the Newcastle-Ottawa scales for cohort and case–control studies.[11] Qualitative literature will be assessed using critical interpretive synthesis.[12] Economic evaluations will be assessed using the Drummond checklist.[13] Studies will be evaluated independently by two reviewers, with a third to settle any disputes. Studies at high risk of bias will not be excluded and conclusions will incorporate observed biases.

Evidence synthesis
The design, methodological characteristics, study quality and main findings will be summarised in narrative and tabular form. We anticipate substantial heterogeneity among included studies precluding the use of meta-analysis techniques.

WP2a: Analysis of routinely collected NHS data
This WP will use routinely collected NHS data to determine when revision happens and to identify patients most likely to require revision in order to target when and on whom follow-up should occur.

Data sources
Data from five national datasets will be used: (1) Clinical Practice Research Database (CPRD),[14] (2) ResearchOne (RO),[15] (3) Hospital Episode Statistics (HES),[16] (4) National Joint Registry (NJR)[17] and (5) patient reported outcome measures (PROMs).[18]

Three linked data sets will be constructed for analysis: (a) CPRD–HES–PROMS, which preexists at the University of Oxford, (b) RO–HES will be constructed and analysed at the University of Leeds. Linkage will be undertaken by NHS Digital on the basis of pseudonyms generated from NHS numbers by the data providers. (c) NJR–HES–PROMS will be constructed and analysed at the University of Oxford. Linkages will be undertaken by NHS Digital, using an agreed set of common patient identifiers, including NHS number. Data sets (a) and (b) provide a primary care view (eg, prior diagnoses, prescribing) and include different, representative patient populations for cross-validation; data set (c) provides a secondary care view (eg, surgeon, procedure details).

Data analysis
The primary outcome of the analysis will be mid-late term revision (>5 years post-primary surgery), defined as the removal, exchange or addition of any of the components of arthroplasty. Exposures will include secondary care predictors, including patient level characteristics recorded in NJR and HES (eg, age, body mass index (BMI)), surgical and operative factors and symptoms of pain, function and health-related quality of life preoperatively and 6 months post-surgery from PROMS, and primary care predictors, including patient demographics, comorbidities and use of drugs which can affect fracture risk. Survival analysis will be used to model time to revision.[19 20] The smoothed Nelson-Aalen cumulative hazard rate will be examined to identify any peak in the mid-long term risk of revision. Cox proportional hazards regression modelling will be used to identify preoperative, perioperative and postoperative predictors of mid-late term revision, for example, age, BMI, comorbidities, implant type, surgeon skill and postoperative problems. Competing risk

For peer review only

BMJ Open: first published as 10.1136/bmjopen-2019-031351 on 25 June 2019. Downloaded from http://bmjopen.bmj.com/ on 15 July 2019 by guest. Protected by copyright.

regression will be used, since mortality can be regarded as a competing risk for revision surgery.[21 22] To account for clustering within the data (such as patients nested within hospitals), a multilevel survival model will be fitted by extending the survival regression model to include a frailty term with a Gaussian distribution.[23]

WP2b part 1: multicentre observational prospective cohort

Prospective data collection from patients undergoing revision surgery.

Objectives:

- Identify all recent (previous 12 months) medical appointments and advice sessions related to the index joint in primary and secondary care.
- Establish if the patient has been seen by orthopaedic health professionals from 12 months after primary surgery until this hospital admission, that is, was the revision directed by routine follow-up.

Design

A multicentre, observational, single visit, prospective cohort study of patients admitted for revision hip or knee surgery.

Population

Patients presenting for elective and emergency revision surgery of a primary THA, TKA or UKA and who are able and willing to provide written informed consent will be included in the study. Patients will be excluded if they have had previous revision surgery; metal-on-metal primary joint replacement or hip hemiarthroplasty. Participants will be recruited from a sample of hospitals selected to provide geographical spread and representation of teaching hospitals, district general hospitals and hospitals with a special interest in joint replacement

Data collection

A participant case report form (CRF) will capture details of follow-up after primary surgery and pathway to current revision surgery, including symptom state. An investigator CRF will extract data from medical notes including demographics (age, gender, diagnosis leading to primary surgery, medical history), general practitioner and hospital appointments, details of primary and revision surgery (including implant type, complications, length of stay). The participant CRF will be piloted with the Leeds Biomedical Research Centre Patient and Public Involvement (PPI) group and the investigator CRF with two research nurses to ascertain the comprehension, usability and completeness of data subsequently extracted.

Sample size

We will use stratified sampling to recruit centres of varying size and anticipate that the average number of patients per centre will be 45 (based on NJR records and information from prospective centres). We initially anticipated the recruitment of 25 centres. With a recruitment rate of 60%, this gave 27 recruited patients from 25 centres (n=675). We do not know the intraclass correlation coefficient (ICC) for our primary outcome ('Was the revision a result of routine follow-up?'), but we anticipate it to be in the region of 0.01–0.05. To be conservative, we use ICC=0.05. This gives a design factor of 2.3 and hence an effective sample size of 293 after accounting for clustering within centre. The enrolment of 35 centres reduced the design factor to 1.6 and the total sample size required to 455. From previous research,[6] we estimate that the rate of our primary outcome is 20% so that the effective number of events will be 58. Hence, we will have sufficient power for our logistic regression to robustly estimate the coefficients of up to five potential risk factors derived from our brief patient survey.[24]

Analysis

The primary outcome will be 'revision identified through routine follow-up', and this will be modelled through a multilevel logistic regression model, with a centre-level random intercept of particular interest. The size of the centre-level effect will be assessed as the proportion of variance explained and will also be assessed through a likelihood ratio test. Up to five factors from the patient questionnaire will be explored as fixed effects at the patient level. This will adjust for case mix. Factors that are found to be both clinically and statistically significant could potentially contribute to a stratified approach to follow-up.

WP2b part 2: qualitative study

Building on previous work highlighting the changes in follow-up practice,[6] this WP aims to explore the rationale and motivating factors behind these changes, the facilitators and the evidence considered when implementing new pathways, including no follow-up.

Sampling

A sample of n=20–30 orthopaedic practitioners and/or unit managers will be recruited. Purposive sampling via sampling matrix will recruit participants with different experiences of a range of follow-up pathways while reflecting NHS trust type, geographical area (urban, rural); socioeconomic area (low/high socioeconomic status) and diverse ethnicity. Some selection criteria are likely to be nested (eg, hospital type, geographical area) and care will be taken to ensure that all viewpoints are represented.

Data collection

Semistructured, telephone interviews following a topic guide refined from the literature review and expert opinion (clinician coapplicants/advisors and PPI members). The researcher will probe pertinent initial responses and expand on issues raised. Interviews will be recorded and transcribed verbatim.

Data analysis

The guiding approach will be framework analysis.[25] Data analysis will comprise five stages: (1) data familiarisation;

For peer review only

BMJ Open: first published as 10.1136/bmjopen-2019-031351 on 25 June 2019. Downloaded from http://bmjopen.bmj.com/ on 15 July 2019 by guest. Protected by copyright.

For peer review only - http://bmjopen.bmj.com/site/about/guidelines.xhtml

(2) identifying the thematic framework; (3) indexing; (4) charting and (5) mapping and interpreting. The process of familiarisation enables the researcher to identify emerging themes or issues in the data. Little is known about why NHS trusts have chosen to either withdraw follow-up care or change the way it is delivered. The evidence generated from the literature review and input from our clinical coapplicants will be used to help identify and refine the thematic framework. Themes are flexible and can be modified in the light of new data, and a process of constant comparison will be undertaken across themes and cases.

WP3

As previous work conducted by members of our team has identified considerable heterogeneity in current follow-up pathways,[6] our cost-effectiveness analysis will compare the relative costs and quality-adjusted life years associated with having follow-up compared with not having follow-up. A third hypothetical scenario of a virtual follow-up will be considered.

Comparators

Both the findings from our systematic review and the prospective cohort will inform the criteria to be used to identify patients as having or not having follow-up. The 7-year reference point for a follow-up currently suggested by BHS and BOA guidelines is likely to be incorporated. Patients having an orthopaedic outpatient appointment around the reference point(s) following a primary arthroplasty will be used to group patients in the CPRD–HES–PROMS data set into the follow-up and no follow-up groups. Joint-specific revision procedures will be identified by OPCS-4 codes as reported in the Admitted Patient Care data set within HES, with corresponding linked records to primary care and PROMS.

Model structure

To identify the most appropriate modelling approach for the question and data at hand, we will conduct a series of preliminary analysis to determine if a cohort-level or patient-level decision analytic model should be employed. Previous models examining the long-term cost-effectiveness of hip and knee replacements have used cohort Markov models.[26 27] Analyses will include associations between patients' characteristics and revision rates, health utilities and costs and whether the risk for revision depends on the time patients stay unrevised after their primary. Regardless of the chosen model type, the key health state or event will be revision arthroplasty, with death and complications also considered. The model will be designed to cover patients' lifetime and analysed from an NHS and Personal Social Services perspective, with discounting of costs and outcomes as per current guide to the methods of technology appraisal.[28]

Model inputs

WP2 data sets will be used to quantify primary and hospital healthcare resource use for comparator groups of follow-up care models through estimation of NHS costs and health-related quality of life (HRQoL). The economic model will simulate long-term costs and quality adjusted life years (QALYs) associated with each care model. Primary care costs will include consultations, and hospital costs will be derived by grouping hospital episodes into Health Resource Groups, a set of casemix groupings utilising similar levels of healthcare resources. Panel data regression analysis[29–31] will be used to estimate hospital costs conditional on patient characteristics and comorbidities. QALYs and transition probabilities will be derived from the linked data sets and published literature as needed. The hypothetical costs of virtual follow-up will be based on similar virtual clinic alternatives previously studied and NHS X-ray-associated costs.

Analysis

Cost-effectiveness analyses will be performed separately for relevant patient subgroups based on gender, age and other potential covariates for which data may be available. As with all economic models, a number of assumptions will be made, and their plausibility and potential impact discussed, relating to model structure and input parameters for transition probabilities, health utilities and costs, including the cost of periprosthetic fractures if no reference is found for these in the literature. Sensitivity analyses will be conducted to explore the uncertainty associated with key assumptions and model parameters and the implications of using different estimates discussed.

WP4: Delphi-consensus process

This WP will use the collective evidence from WP1–3 to inform a consensus process to determine appropriate follow-up care pathways for hip and knee arthroplasty.

Evidence gathered from WP1–3 will feed into a consensus panel workshop. We intend to use methods employed by the National Institute for Health and Care Excellence (NICE) in both the technology assessment committees and Guideline Development Groups. The expert stakeholders invited to attend will have a special interest in patient follow-up after hip or knee replacement surgery. Participants will include patients, orthopaedic surgeons, arthroplasty practitioners, NHS managers and commissioners, manufacturers and representatives of the major orthopaedic bodies (including BOA, BHS and BASK). The purpose of this exercise is to consider the evidence and obtain agreement for future care pathways, supported by the evidence of their effectiveness and cost-effectiveness, to be recommended and adopted across the NHS. Following the NICE consensus model all participants will receive summaries of the main research findings in advance. There will be presentations from the work-stream leaders to outline the evidence for consideration.

Robert *et al*[7] demonstrate that decommissioning is often about more than the 'evidence' and that withdrawal of previously available services is often seen as being driven by the wrong kind of evidence, based on cost data and

BMJ Open: first published as 10.1136/bmjopen-2019-031351 on 25 June 2019. Downloaded from http://bmjopen.bmj.com/ on 15 July 2019 by guest. Protected by copyright.

For peer review only - http://bmjopen.bmj.com/site/about/guidelines.xhtml

political priorities and not on what patients and service users value.[7] It is a complex issue, perhaps as contentious as NICE decisions when they do not fund an effective intervention because it exceeds the threshold. However, NICE investment decisions are made with the explicit understanding that, with no increase in the budget, there must be some displacement of other healthcare technologies.[32] We plan to make use of the recommendations for engagement and the use of evidence outlined in Robert *et al* to ensure the results of this work are understood and considered as a genuine attempt to use the best evidence available to ensure that the NHS gets value for money and that patients remain safe.

Patient and public involvement

Members of the NIHR Leeds BRC, Oxford and Bristol PPI groups are involved in UK SAFE. The PPI co-applicant is a member of the study steering committee and contributes across all WPs. Two independent PPI advisors sit on the Independent Advisory Group. Specific areas where lay involvement will be pivotal include the interpretation of results of the systematic review, the expert panel discussion and consensus process, study oversight (steering group), preparation of patient material and study results and contribution to reports and newsletters for patients and NHS staff.

ETHICS AND DISSEMINATION

All studies will be conducted in accordance with the principles of Good Clinical Practice, and the UK Policy Framework for Health and Social Care Research, 2018. Favourable ethical opinion has been obtained for WP2a (RO-HES) (220520) and WP2B (220316) from the National Research Ethics Committee. Following advice from the Confidentiality Advisory Group (17/CAG/0122), data controllers for the data sets used in WP2a (RO–HES)—NHS Digital and The Phoenix Partnership—confirmed that Section 251 support was not required as no identifiable data was flowing into or out of these parties. Application for approval of WP2a (RO–HES) from the Independent Group Advising on the Release of Data (IGARD) at NHS Digital is in progress (DARS-NIC-147997). Section 251 support (17/CAG/0030) and NHS Digital approval (DARS-NIC-172121-G0Z1H-v0.11) have been obtained for WP2a (NJR-HES-PROMS). ISAC (11_050MnA2R2) approval has been obtained for WP2a (CPRD–HES).

At the end of the project, outputs will be disseminated nationally in the form of an executive summary statement of the agreed pathway/s through appropriate NHS Networks, NICE, the NHS England Elective Orthopaedics Sub-committee, the NHS Institute for Innovation and Improvement and professional societies, including BHS, BOA, BASK, Arthroplasty Care Practitioners Association and the NJR. Dissemination will be key to developing a culture of 'finding the best way of doing something and doing it everywhere' to significantly reduce wastage of clinical resources and optimise NHS spend. We will put forward the consensus statement to each society's AGM for adoption as a resolution. Internationally, dissemination platforms are in place through the International Society of Arthroplasty Registers (ISAR) and the European Federation of National Associations of Orthopaedics and Traumatology. A lay summary of the project will be produced for study participants. Findings will also be presented at relevant orthopaedic and methodological conferences, such as the BOA and the Exploiting Existing Data for Health Research conference. The chief investigator and co-applicants will be named as authors on main publications, and an appropriate first author agreed through discussion. Other key individuals will be included as authors or contributors as appropriate, at the discretion of the Senior Management Group. Any disputes relating to authorship will be resolved by the Steering Committee.

The Chair and Independent members of the Steering Committee will be acknowledged, but will not qualify for full authorship, in order to maintain their independence. Individual collaborators must not publish data concerning their participants' which are directly relevant to the questions posed in the study until the main results of the study have been published.

CONCLUSION

This research will deliver the first research-supported, best-for-patient, joint-specific, cost-effective recommendations for follow-up pathways, providing a gold standard for clinical excellence and follow-up advice for patients, surgeons, purchasers and the NHS as a whole. Value is not limited to the UK, but has substantial global impact potential.

The impact of this work will be to reduce the burden on patients and the NHS in terms of outpatient visits and clinical tests that do not add benefit, while optimising detection of potential problems. From an NHS perspective, this work will provide managers with economic and clinical information on arthroplasty follow-up to inform service planning and delivery, and the role of arthroplasty practitioners in this service, with the potential to reduce geographical disparity through NHS trusts modelling their service provision on a national evidence-based guideline; provide orthopaedic surgeons with guidance on follow-up, including patient and economic considerations of factors involved; produce arthroplasty follow-up guidelines for adoption by the relevant specialist societies and information for their members. From a patient perspective, this work will help to inform patients about follow-up practice, empower them to make choices about future healthcare relating to their joint arthroplasty and provide reassurance that their follow-up pathway is appropriate

The outputs of this project, in terms of evidence-based support for timing of follow-up and identification of the most cost-effective follow-up model, fit directly within the

BMJ Open: first published as 10.1136/bmjopen-2019-031351 on 25 June 2019. Downloaded from http://bmjopen.bmj.com/ on 15 July 2019 by guest. Protected by copyright.

For peer review only - http://bmjopen.bmj.com/site/about/guidelines.xhtml

NHS framework for improving outcomes from elective procedures. Rationalising current diversity of follow-up practices should enable substantial savings for the NHS. We envisage outputs to be readily applicable to the wider NHS, not only hip and knee but also other joint replacements. With the committed support of key national and international organisations already in place, we anticipate that these guidelines will be positively received and that implementation will be widespread.

Author affiliations
[1]Leeds Institute of Health Sciences, University of Leeds, Leeds, UK
[2]Leeds Institute of Rheumatic and Musculoskeletal Medicine, University of Leeds, Leeds, UK
[3]NIHR Leeds Biomedical Research Centre, Leeds, UK
[4]Nuffield Department of Orthopaedics Rheumatology and Musculoskeletal Sciences, University of Oxford, Oxford, UK
[5]Musculoskeletal Research Unit, Bristol Medical School, University of Bristol, Southmead Hospital, Bristol, UK
[6]MRC Lifecourse Epidemiology Unit, Southampton, UK
[7]Manor Park Surgery, Leeds, UK
[8]Trauma and Orthopaedics, Weston Area Health NHS Trust, Weston-super-Mare, UK
[9]Department of Orthopaedic Surgery, Leeds Teaching Hospitals NHS Trust, Leeds, Leeds, UK

Contributors All authors contributed to the planning and design of this study, drafted the article, revised the article and approved the final manuscript.

Funding This article presents independent research funded by the National Institute for Health Research (NIHR) Health Services and Delivery Research Programme (14/70/146) and by the NIHR Leeds Biomedical Research Centre (BRC).

Disclaimer The views expressed are those of the author(s) and not necessarily those of the NIHR or the Department of Health and Social Care.

Competing interests None declared.

Patient consent for publication Not required.

Provenance and peer review Not commissioned; peer reviewed for ethical and funding approval prior to submission.

Open access This is an open access article distributed in accordance with the Creative Commons Attribution 4.0 Unported (CC BY 4.0) license, which permits others to copy, redistribute, remix, transform and build upon this work for any purpose, provided the original work is properly cited, a link to the licence is given, and indication of whether changes were made. See: https://creativecommons.org/licenses/by/4.0/.

REFERENCES

1. National Joint Registry for England, Wales. Northern Ireland and the Isle of Man: 15th Annual report. 2018 http://www.njrreports.org.uk/
2. Vanhegan IS, Malik AK, Jayakumar P, *et al*. A financial analysis of revision hip arthroplasty: the economic burden in relation to the national tariff. *J Bone Joint Surg Br* 2012;94:619–23.
3. Briggs T. *Getting it right first time. Improving the quality of orthopaedic care within the National Health Service in England*, 2012.
4. Singh JA. Epidemiology of knee and hip arthroplasty: a systematic review. *Open Orthop J* 2011;5:80–5.
5. British Orthopaedic Association. *Primary total hip replacement: a guide to good practice*, 2012.
6. Smith LK. A survey of the current state of hip arthroplasty surveillance in the United Kingdom. *Musculoskeletal Care* 2014;12:232–8.
7. Robert G, Harlock J, Williams I. Disentangling rhetoric and reality: an international Delphi study of factors and processes that facilitate the successful implementation of decisions to decommission healthcare services. *Implementation Science* 2014;9:123.
8. Higgins JPT. *Cochrane Handbook for Systematic Reviews of Interventions Version 5.1.0*: The Cochrane Collaboration, 2011. Available from. http://handbook.cochrane.org
9. Liberati A, Altman DG, Tetzlaff J, *et al*. The PRISMA statement for reporting systematic reviews and meta-analyses of studies that evaluate health care interventions: explanation and elaboration. *PLoS Med* 2009;6:e1000100.
10. Higgins JP, Altman DG, Sterne JA. Chapter 8: Assessing risk of bias in included studies in Cochrane handbook for systematic reviews of interventions Version 5.1.0. 2011 http://handbook.cochrane.org
11. Wells G, Shea B, O'Connell D, *et al*. The Newcastle-Ottawa Scale (NOS) for assessing the quality of nonrandomised studies in meta-analyses. 2014 http://www.ohri.ca/programs/clinical_epidemiology/oxford.asp;
12. Dixon-Woods M, Cavers D, Agarwal S, *et al*. Conducting a critical interpretive synthesis of the literature on access to healthcare by vulnerable groups. *BMC Med Res Methodol* 2006;6:35.
13. Drummond MF, Schulpher MJ, Claxton K, *et al*. *Methods for the Economic Evaluation of Health Care Programmes*. 4th edn. Oxford: Oxford University Press, 2015.
14. Clinical Practice Research Datalink. http://www.cprd.com
15. ResearchOne. http://www.researchone.org
16. Hospital Episode Statistics (HES). http://www.hscic.gov.uk/hes
17. Welcome from the National Joint Registry for England, Wales, Northern Ireland and the Isle of Man. http://www.njrcentre.org.uk
18. http://www.hscic.gov.uk/hesproms
19. Pinedo-Villanueva R, Turner D, Raftery JP, *et al*. Outcomes after total hip replacement. *Osteoarthritis Cartilage* 2014;22:S214.
20. Pinedo-Villanueva R, Turner D, Raftery JP, *et al*. Primary care costs attributable to osteoarthritis before total hip replacement. *Osteoarthritis Cartilage* 2014;22:S210–1.
21. Jason PF, Gray RJ. A proportional hazards model for the subdistribution of a competing risk. *Journal of the American Statistical Association* 1999;94:496–509.
22. Prieto-Alhambra D, Pagès-Castellà A, Wallace G, *et al*. Predictors of fracture while on treatment with oral bisphosphonates: a population-based cohort study. *J Bone Miner Res* 2014;29:268–74.
23. Hirsch K, Wienke A. Software for semiparametric shared gamma and log-normal frailty models: An overview. *Comput Methods Programs Biomed* 2012;107:582–97.
24. Peduzzi P, Concato J, Kemper E, *et al*. A simulation study of the number of events per variable in logistic regression analysis. *J Clin Epidemiol* 1996;49:1373–9.
25. Wallace G, Judge A, Prieto-Alhambra D, *et al*. The effect of body mass index on the risk of post-operative complications during the 6 months following total hip replacement or total knee replacement surgery. *Osteoarthritis Cartilage* 2014;22:918–27.
26. Burn E, Liddle AD, Hamilton TW, *et al*. Cost-effectiveness of unicompartmental compared with total knee replacement: a population-based study using data from the National Joint Registry for England and Wales. *BMJ Open* 2018;8:e020977.
27. Fitzpatrick R, Shortall E, Sculpher M, *et al*. Primary total hip replacement surgery: a systematic review of outcomes and modelling of cost-effectiveness associated with different prostheses. *Health Technol Assess* 1998;2:1–64.
28. Guide to the methods of technology appraisal 2013. https://www.nice.org.uk/process/pmg9/chapter/foreword
29. Basu A, Rathouz PJ. Estimating marginal and incremental effects on health outcomes using flexible link and variance function models. *Biostatistics* 2005;6:93–109.
30. Clarke P, Leal J, Kelman C, *et al*. Estimating the cost of complications of diabetes in Australia using administrative health-care data. *Value Health* 2008;11:199–206.
31. Mullahy J. Much ado about two: reconsidering retransformation and the two-part model in health econometrics. *J Health Econ* 1998;17:247–81.
32. NICE technology appraisal guidance. https://www.nice.org.uk/about/what-we-do/our-programmes/nice-guidance/nice-technology-appraisal-guidance

For peer review only

BMJ Open: first published as 10.1136/bmjopen-2019-031351 on 25 June 2019. Downloaded from http://bmjopen.bmj.com/ on 15 July 2019 by guest. Protected by copyright.

For peer review only - http://bmjopen.bmj.com/site/about/guidelines.xhtml

Supplementary file II: STROBE checklist

STROBE. Strengthening the reporting of observational studies in epidemiology. Available at: https://www.strobe-statement.org/. [Last accessed 29 September 2021].

	Item No	Recommendation	Page No
**Title and abstract**	1	(*a*) Indicate the study's design with a commonly used term in the title or the abstract	1
		(*b*) Provide in the abstract an informative and balanced summary of what was done and what was found	2
		**Introduction**	
Background/rationale	2	Explain the scientific background and rationale for the investigation being reported	4
Objectives	3	State specific objectives, including any prespecified hypotheses	4
		**Methods**	
Study design	4	Present key elements of study design early in the paper	5
Setting	5	Describe the setting, locations, and relevant dates, including periods of recruitment, exposure, follow-up, and data collection	5,6,8
Participants	6	(*a*) Give the eligibility criteria, and the sources and methods of selection of participants. Describe methods of follow-up	6,8
		(*b*) For matched studies, give matching criteria and number of exposed and unexposed	n/a
Variables	7	Clearly define all outcomes, exposures, predictors, potential confounders, and effect modifiers. Give diagnostic criteria, if applicable	7,8
Data sources/ measurement	8*	For each variable of interest, give sources of data and details of methods of assessment (measurement). Describe comparability of assessment methods if there is more than one group	5,6,8,9
Bias	9	Describe any efforts to address potential sources of bias	n/a
Study size	10	Explain how the study size was arrived at	8
Quantitative variables	11	Explain how quantitative variables were handled in the analyses. If applicable, describe which groupings were chosen and why	8,9
Statistical methods	12	(*a*) Describe all statistical methods, including those used to control for confounding	8,9
		(*b*) Describe any methods used to examine subgroups and interactions	n/a
		(*c*) Explain how missing data were addressed	8
		(*d*) If applicable, explain how loss to follow-up was addressed	n/a
		(*e*) Describe any sensitivity analyses	9
**Results**			
Participants	13*	(a) Report numbers of individuals at each stage of study—eg numbers potentially eligible, examined for eligibility, confirmed eligible, included in the study, completing follow-up, and analysed	10
		(b) Give reasons for non-participation at each stage	10
		(c) Consider use of a flow diagram	

For peer review only - http://bmjopen.bmj.com/site/about/guidelines.xhtml

Descriptive data	14*	(a) Give characteristics of study participants (eg demographic, clinical, social) and information on exposures and potential confounders	Supp File 3: Tables A&B
		(b) Indicate number of participants with missing data for each variable of interest	n/a
		(c) Summarise follow-up time (eg, average and total amount)	n/a
Outcome data	15*	Report numbers of outcome events or summary measures over time	11

Main results	16	(*a*) Give unadjusted estimates and, if applicable, confounder-adjusted estimates and their precision (eg, 95% confidence interval). Make clear which confounders were adjusted for and why they were included	13,14,15,16,17
		(*b*) Report category boundaries when continuous variables were categorized	n/a
		(*c*) If relevant, consider translating estimates of relative risk into absolute risk for a meaningful time period	11
Other analyses	17	Report other analyses done—eg analyses of subgroups and interactions, and sensitivity analyses	13,14,15,16,17
**Discussion**			
Key results	18	Summarise key results with reference to study objectives	18
Limitations	19	Discuss limitations of the study, taking into account sources of potential bias or imprecision. Discuss both direction and magnitude of any potential bias	18
Interpretation	20	Give a cautious overall interpretation of results considering objectives, limitations, multiplicity of analyses, results from similar studies, and other relevant evidence	19, 20
Generalisability	21	Discuss the generalisability (external validity) of the study results	20
**Other information**			
Funding	22	Give the source of funding and the role of the funders for the present study and, if applicable, for the original study on which the present article is based	21

*Give information separately for exposed and unexposed groups.

For peer review only

Supplementary file III: Additional results

Table A. Descriptive statistics for the CPRD-HES linked datasets

	Knee Replacement
Year of primary	
1995-1999	995 (5.7%)
2000-2004	4486 (25.8%)
2005-2009	8415 (48.4%)
2010-2011	3482 (20.0%)
Age at primary	69.4 (SD 9.2)
Sex	
Female	9963 (57.3%)
Male	7415 (42.7%)
Body mass index	
Underweight	48 (0.4%)
Normal	2204 (16.2%)
Overweight	5239 (38.6%)
Obese Class I (Moderately obese)	3777 (27.8%)
Obese Class II and higher	2306 (17.0%)
Index of Multiple Deprivation (IMD) quintiles	
Least deprived	3890 (22.4%)
2	4145 (23.9%)
3	3918 (22.6%)
4	3078 (17.7%)
Most deprived	2328 (13.4%)
Region	
East Midlands	706 (4.1%)
East of England	2003 (11.5%)
London	1464 (8.4%)
North East	434 (2.5%)
North West	2551 (14.7%)
South Central	2605 (15.0%)
South East Coast	2144 (12.3%)
South West	2386 (13.7%)
West Midlands	2320 (13.4%)
Yorkshire & The Humber	765 (4.4%)
Smoker	
Ex-smoker	5122 (34.6%)
Non-smoker	8310 (56.2%)
Current	1368 (9.2%)
Alcohol	

For peer review only

Ex-smoker	322 (2.7%)
No	2242 (18.8%)
Yes	9371 (78.5%)
Recorded diagnosis of hip OA	6841 (39.4%)
Hip Fracture prior primary surgery	119 (0.7%)
Fracture in pelvis, proximal/humerus, wrist/forearm, spine or rib	537 (3.1%)
Comorbidities	
asthma	1713 (9.9%)
malabsorption	42 (0.2%)
inflammatory bowel disease	128 (0.7%)
hypertension	6106 (35.1%)
hyperlipidaemia	2223 (12.8%)
ischaemic heart disease	1685 (9.7%)
myocardial infarction	345 (2.0%)
stroke/cerebrovascular disease	585 (3.4%)
chronic pulmonary disease	498 (2.9%)
chronic kidney failure	1277 (7.4%)
cancer	1446 (8.3%)
diabetes	1774 (10.2%)
Drugs which can affect fracture risk prior primary surge	
Calcium and vitamin D supplements	1377 (7.9%)
Bisphosphonates	1161 (6.7%)
Selective oestrogen receptor modulators	33 (0.2%)
Oral glucocorticosteroid therapy	3521 (20.3%)
Drugs prior primary surgery	
Proton pump inhibitors	7586 (43.7%)
Anti-arrhythmics	1700 (9.8%)
Anticonvulsants	865 (5.0%)
Antidepressants	5875 (33.8%)
Anti-Parkinson drugs	305 (1.8%)
Statins	5697 (32.8%)
Thiazide diuretics	8498 (48.9%)
Anxiolitics	3406 (19.6%)
Painkillers/anti-inflammatory drugs	
NSAIDs	15406 (88.7%)
NSAID cox	3155 (18.2%)
Paracetamol	14438 (83.1%)
Partial Opiates	13334 (76.7%)
Total Opiates	6459 (37.2%)
Steroid iFuse Implant System® Minimally Invasive Arthrodesis (iMIA)	5401 (31.1%)
DDDs (daily defined dose) 1-year prior surgery	

For peer review only

Calcium and vitamin D supplements	
No dose	16001 (92.1%)
<120 DDD	281 (1.6%)
>=120 to 340 DDD	503 (2.9%)
>340 DDD	222 (1.3%)
Dose missing	371 (2.1%)
Bisphosphonates	
No dose	16217 (93.3%)
<140 DDD	229 (1.3%)
>=140 to 340 DDD	374 (2.2%)
>340 DDD	260 (1.5%)
Dose missing	298 (1.7%)
Selective oestrogen receptor modulators	
No dose	17345 (99.8%)
<280 DDD	8 (0.1%)
>=280 to 390 DDD	8 (0.1%)
>390 DDD	0 (0%)
Dose missing	17 (0.1%)
Oral glucocorticosteroid therapy	
No dose	13857 (79.7%)
<30 DDD	493 (2.8%)
>=30 to 280 DDD	458 (2.6%)
>280 DDD	316 (1.8%)
Dose missing	2254 (13.0%)
Proton pump inhibitors (no dose)	
No dose	9792 (56.4%)
<85 DDD	1376 (7.9%)
>=85 to 365 DDD	2847 (16.4%)
>365 DDD	995 (5.7%)
Dose missing	2368 (13.6%)
Anti-arrhythmics (no dose)	
No dose	15678 (90.2%)
<170 DDD	159 (0.9%)
>=170 to 365 DDD	241 (1.4%)
>365 DDD	158 (0.9%)
Dose missing	1142 (6.6%)
Anticonvulsants	
No dose	16513 (95.0%)
<85 DDD	132 (0.8%)
>=85 to 365 DDD	212 (1.2%)
>365 DDD	111 (0.6%)
Dose missing	410 (2.4%)
Antidepressants	

For peer review only

No dose	11503 (66.2%)
<85 DDD	786 (4.5%)
>=85 to 365 DDD	1418 (8.2%)
>365 DDD	565 (3.3%)
Dose missing	3106 (17.9%)
Anti-Parkinson drugs	
No dose	17073 (98.2%)
<200 DDD	36 (0.2%)
>=200 to 600 DDD	90 (0.5%)
>600 DDD	41 (0.2%)
Dose missing	138 (0.8%)
Statins	
No dose	11681 (67.2%)
<280 DDD	1248 (7.2%)
>=280 to 370 DDD	2522 (14.5%)
>370 DDD	1383 (8.0%)
Dose missing	544 (3.1%)
Thiazide diuretics	
No dose	8880 (51.1%)
<225 DDD	1678 (9.7%)
>=225 to 390 DDD	2826 (16.3%)
>390 DDD	1565 (9.0%)
Dose missing	2429 (14.0%)
Anxiolitics	
No dose	13972 (80.4%)
<30 DDD	367 (2.1%)
>=30 to 350 DDD	531 (3.1%)
>350 DDD	344 (2.0%)
Dose missing	2164 (12.5%)
NSAIDs	
No dose	1972 (11.4%)
<60 DDD	2428 (14.0%)
>=60 to 300 DDD	4602 (26.5%)
>300 DDD	2352 (13.5%)
Dose missing	6024 (34.7%)
NSAID cox	
No dose	14223 (81.8%)
<60 DDD	355 (2.0%)
>=60 to 280 DDD	553 (3.2%)
>280 DDD	267 (1.5%)
Dose missing	1980 (11.4%)
Paracetamol	
No dose	2940 (16.9%)

For peer review only

<40 DDD	2796 (16.1%)
>=40 to 200 DDD	5521 (31.8%)
>200 DDD	2425 (14.0%)
Dose missing	3696 (21.3%)
Opioids mix	
No dose	4044 (23.3%)
<30 DDD	2074 (11.9%)
>=30 to 180 DDD	4002 (23.0%)
>180 DDD	1976 (11.4%)
Dose missing	5282 (30.4%)
Opioids total	
No dose	10919 (62.8%)
<200 DDD	995 (5.7%)
>=200 to 600 DDD	1916 (11.0%)
>600 DDD	871 (5.0%)
Dose missing	2677 (15.4%)
Steroid IMIA	
No dose	11977 (68.9%)
<55 DDD	1292 (7.4%)
>=55 DDD	597 (3.4%)
Dose missing	3512 (20.2%)

For peer review only

Table B. Descriptive statistics for the NJR-HES-PROMs linked dataset

		Knee Replacement
Year of primary		
	2008	33504 (17.8%)
	2009	45928 (42.1%)
	2010	52460 (70.0%)
	2011	56617 (100.0%)
Age at primary knee replacement		69.9 (SD 9.3)
Sex		
Female		106812 (56.7%)
Male		81697 (43.3%)
Body mass index		30.9 (SD 5.5)
Index of Multiple Deprivation (IMD) quintiles		
Least deprived		40247 (21.6%)
2		42721 (22.9%)
3		35839 (19.2%)
4		34691 (18.6%)
Most deprived		33119 (17.8%)
Rurality, at primary		
urban >=10,000		140202 (74.5%)
town and fringe		22366 (11.9%)
village/isolated		25618 (13.6%)
ethnicity		
white		161079 (92.9%)
non-white		12237 (7.1%)
Number comorbidities at primary (none)		
None		141570 (75.1%)
Mild		38083 (20.2%)
Moderate		6875 (3.7%)
Severe		1981 (1.1%)
ASA grade		
P1 - Fit and healthy		20087 (10.7%)
P2 - Mild disease not incapacitating		138997 (73.7%)
P3 - P5		29425 (15.6%)
Minimally invasive (no)		
No		176683 (93.7%)
Yes		11826 (6.3%)
Surgical volume per consultant		
<=10 operations		5312 (2.8%)
11-50		58102 (30.8%)
51-75		42101 (22.3%)
76-100		33558 (17.8%)

For peer review only

101-150	32381 (17.2%)
>150	17055 (9.1%)
Surgeon experience	
<8 training years	44650 (23.7%)
Consultant (≥8 training years)	143859 (76.3%)
Surgical approach (knee)	
Lateral parapatellar	1756 (0.9%)
Medial parapatellar	175112 (92.9%)
Mid-Vastus	5710 (3.0%)
Sub-Vastus	2300 (1.2%)
Other	3631 (1.9%)
Primary graft femur	
No	186951 (99.2%)
Yes	1558 (0.8%)
Implant fixation	
Cementless	9429 (5.0%)
cemented	177031 (94.0%)
Hybrid	1947 (1.0%)
Primary graft tibia	
No	187679 (99.6%)
Yes	830 (0.4%)
Type of primary implant	
UKR	13266 (7.0%)
TKR	175243 (93.0%)
Type of mechanical thromboprophylaxis	
None	16746 (8.9%)
Any	171763 (91.1%)
Type of chemical thromboprophylaxis	
None	13239 (7.0%)
Aspirin only	16356 (8.7%)
LMWH (+/-Other)	134648 (71.4%)
Other (no LMWH)	24266 (12.9%)
Unit type	
Public hospital	153780 (81.6%)
Independent sector - hospital	24716 (13.1%)
Independent sector - treatment centre	10013 (5.3%)
OKS, baseline score	18.0 (SD 7.8)
EQ-5D Anxiety Depression	
I am not anxious or depressed	65123 (62.4%)
I am moderately anxious or depressed	32676 (31.3%)
I am extremely anxious or depressed	3710 (3.6%)

For peer review only

Table C. Descriptive statistics comparing patients in the full CPRD dataset to those with linked HES data for the CPRD-HES linked datasets

**Year of primary (2010-2011)**	CPRD (n=37,906)	CPRD-HES (n=17,378)
	3118 (8.2%)	995 (5.7%)
	10011 (26.4%)	4486 (25.8%)
	17920 (47.3%)	8415 (48.4%)
	6857 (18.1%)	3482 (20.0%)
**Age at primary (continuous variable)**		
	69.6 (SD 9.7)	69.4 (SD 9.2)
**Sex (Woman)**		
	16328 (43.1%)	7415 (42.7%)
**Body mass index**		
	120 (0.4%)	48 (0.4%)
	5201 (18.0%)	2204 (16.2%)
	11427 (39.6%)	5239 (38.6%)
	7739 (26.8%)	3777 (27.8%)
	4364 (15.1%)	2306 (17.0%)
**Index of Multiple Deprivation (IMD) quintiles, at primary**		
	7254 (24.5%)	3890 (22.4%)
	7111 (24.0%)	4145 (23.9%)
	6548 (22.1%)	3918 (22.6%)
	5031 (17.0%)	3078 (17.7%)
	3725 (12.6%)	2328 (13.4%)
**Region**		
	2244 (5.9%)	706 (4.1%)
	4547 (12.0%)	2003 (11.5%)
	3436 (9.1%)	1464 (8.4%)
	1033 (2.7%)	434 (2.5%)
	5066 (13.4%)	2551 (14.7%)
	5537 (14.6%)	2605 (15.0%)
	4894 (12.9%)	2144 (12.3%)
	4738 (12.5%)	2386 (13.7%)
	4589 (12.1%)	2320 (13.4%)
	1822 (4.8%)	765 (4.4%)
**Smoker**		
	10576 (33.4%)	5122 (34.6%)
	18106 (57.2%)	8310 (56.2%)
	2971 (9.4%)	1368 (9.2%)
**Drink alcohol**		
	634 (2.5%)	322 (2.7%)
	4657 (18.2%)	2242 (18.8%)
	20367 (79.4%)	9371 (78.5%)

For peer review only
